# Bioactive metabolites identified from *Aspergillus terreus* derived from soil

**DOI:** 10.1186/s13568-023-01612-0

**Published:** 2023-10-03

**Authors:** Menna Fayek, Hassan Y. Ebrahim, Mohamed S. Abdel-Aziz, Heba Taha, Fatma A. Moharram

**Affiliations:** 1 Pharmacognosy Department, Faculty of Pharmacy, Helwan University, Cairo, 11795 Egypt; 2 Department of Microbial Chemistry Department, Genetic Engineering and Biotechnology Division, National Research Centre, Giza, 12622 Egypt; 3 Biochemistry and Molecular Biology Department, Faculty of Pharmacy, Helwan University, Cairo, 11795 Egypt

**Keywords:** *Aspergillus terreus*, ACE2 inhibition, Anti-inflammatory, Antimicrobial, HPLC/MS, Secondary metabolites

## Abstract

*Aspergillus terreus* has been reported to produce many bioactive metabolites that possess potential activities including anti-inflammatory, cytotoxic, and antimicrobial activities. In the present study, we report the isolation and identification of *A. terreus* from a collected soil sample. The metabolites existing in the microbial ethyl acetate extract were tentatively identified by HPLC/MS and chemically categorized into alkaloids, terpenoids, polyketides, γ-butyrolactones, quinones, and peptides. In addition, a new triglyceride (**1**) and a diketopiperazine derivative namely asterrine (**4**), together with two known butyrolactone (**2–3**) were purified from the extract. The chemical skeleton of the purified compounds was established by comprehensive analysis of their ESI/MS, 1 and 2D-NMR data. The extract and compounds **3**,**4** exhibited a strong inhibitory activity for the binding of ACE2 to SARS-CoV-2 spike-protein receptor with IC_50_ 7.4, 9.5, and 8.5 µg/mL, respectively. In addition, the extract, **1** and **2** displayed a potent anti-inflammatory effect with IC_50_ 51.31 and 37.25 pg/mL (Il-6) and 87.97, 68.22 pg/mL (TNF-α), respectively, in comparison to LPS control. In addition, the extract and compound **4** displayed an antimicrobial effect towards *S. aureus* by MIC 62.5 and 125 μg/mL, while the extract exhibited a potent effect against *C. albicans* (MIC of 125 μg/mL). Collectively, our data introduce novel bioactivities for the secondary metabolites produced by the terrestrial fungus *Aspergillus terreus*.

**Supplementary Information:**

The online version contains supplementary material available at 10.1186/s13568-023-01612-0.

## Key points

- Identification of *Aspergillus terreus* metabolites using HPLC/MS analysis. 
- Structure elucidation of isolated metabolites from *Aspergillus terreus* extract using different spectroscopic techniques.
- The microbial extract and purified biomolecules exhibited significant anti-inflammatory, antimicrobial, and ACE2 inhibitory activities..

**Supplementary Information:**

The online version contains supplementary material available at 10.1186/s13568-023-01612-0.

## Introduction

Angiotensin-converting enzyme II (ACE2) is an essential membrane protein found in many cells of the human body (Zhou et al. 2020) and it is considered the main part of the renin-angiotensin system (RAS) (Sharma et al. 2020). It catalyzes the conversion of angiotensin II (Ang II), a strong vasoconstrictor and pro-inflammatory molecule, to Ang 1–7, consequently, it regulates the RAS via lowering Ang II (Datta et al. 2020). ACE2 receptor is the main target for severe acute respiratory syndrome coronavirus (SARS-CoV-2) as it plays a fetal role in the virus′s spread to alveolar cells (Bahbah et al. 2020). ACE2 not only allows the attack and quick replication of SARS-CoV-2 but also leads to the elevation of Ang II which in turn activates NF-κB and pro-inflammatory cytokines release (Dandona et al. 2007; Benigni et al. 2010), as well as, stimulation of TNF-α/IL-6 pathways (Hirano and Murakami 2020), resulting in acute destruction for the tissues of the lung (Chen et al. 2020a). SARS-CoV-2 attaches to human ACE2 via binding the protein of spike (S), which contains S1 and S2 subunits (Othman et al. 2020). Also, it was reported that ACE2 expression is negatively linked with COVID-19 mortality (Chen et al. 2020a, b). Therefore, ACE2 blockade may inhibit the SARS-CoV-2-S protein from fusing and entering the cells of the host. In the way to discover new COVID-19 therapies, metabolites from natural sources were tested for ACE2 receptor inhibition (Islam et al. 2020). Moreover, COVID-19 syndrome pathology was noticed to be related to the hyper-inflammatory response referred to as cytokine storm, characterized by an extreme elevation in the circulating levels of pro-inflammatory IL-1, -2, and -6, IFN-γ, and TNF-α. Moreover, it was reported that bioactive metabolites may become a promising tool for the treatment of SARS-CoV-2 infection by reducing inflammation in COVID-19 patients, in link with classical anti-inflammatory agents (Giovinazzo et al. 2020).

The escalation of antimicrobial resistance and the simultaneous growth in multi-drug resistant microorganisms have exposed critical threats to public health and the healthcare system. Every year, millions of deaths are firmly linked to microbial infections as a consequence of lacking proper treatments, (O′Neill 2014). Moreover, the increase in antimicrobial resistance have reduced the effectiveness of antimicrobial therapies (Laws et al. 2019). Consequently, the search for alternative antimicrobial drug candidates is highly warranted. Over the past time, natural compounds have been greatly trusted upon therapeutic agent sources, including antimicrobial agents. Particularly, they represent more than two-thirds of a newly accepted pharmaceutical preparations (Newman and Cragg 2012). Unlike antibiotics that originated from microbes, antimicrobials obtained from natural products have been widely explored for diverse medicinal applications. Terrestrial fungi are remarkable producers of novel secondary metabolites with different biological activites as antimicrobial, anticancer, and anti-inflammatory bioactivity (Elissawy et al. 2021; El-Demerdash et al. 2018; Karpinski and Marine 2019). *Aspergillus* spp. yields a wide variety of structurally heterogeneous metabolites, that are of significant attention to the scientific research community. They produce industrial (Yoon et al. 2013; Singh et al. 2016) and medicinally important secondary metabolites as antibiotics and the cholesterol lowering statins (Fu et al. 2015). *A. terreus* is one of the most common *Aspergillus* spp. isolated from plants and soil, and it is widely used in chemical and pharmaceutical industries. As well as, it considered a creative producer of biologically active secondary metabolites such as butyrolactone I (Ghfar et al. 2021; Hamed et al. 2020), alkaloids (El‐Hawary et al. 2021; Qi et al. 2020), polyketides (El‐Hawary et al. 2021; Deng et al. 2020), and terpenoids (Uras et al. 2021; Girich et al. 2020). In the course of our research on fungi collected from the soil as sources of new biologically active metabolites, we report a tentative identification of diverse metabolites from the fungus *A. terreus* purified from the soil using HPLC/MS analysis. In addition, we describe the identification of two new natural compounds along with two known ones. Moreover, the inhibitory activity of ACE2 protein, anti-inflammatory and antimicrobial activities for the extract and pure metabolites were evaluated.

## Material and methods

### General

Silica gel 60-120 mesh and F_254_ were used as stationary phases for column (CC) and thin layer chromatography (TLC), respectively (Merck, Darmstadt, Germany). ^1^H and ^13^C NMR spectra were recorded on a Bruker Avance spectrometer (Bruker, Rheinstetten, Germany) and JEOL (Tokyo, Japan) at 400 and 500 MHz for ^1^HNMR, as well as, 100 and 125 MHz for ^13^CNMR. The results were expressed as δ ppm relative to the internal reference (TMS). HPLC/ESI-MS were recorded in negative and positive ionization modes on an XEVO TQD triple quadruple LC/MS/MS (Waters Corporation, Milford, MA01757 U.S.A). Chromatographic conditions: ACQUITY UPLC—BEH C18 column (1.7µm, 2.1 mm × 50 mm), flow rate (0.2 mL\min); mobile phase (water and acetonitrile each containing 0.1% formic acid).

### Material used for fungal isolation

The sample of soil was collected in December 2019 from an agriculture field (10 cm depth, Dekernis, Egypt). For the preparation of soil suspension, 20 g was mixed with sterilized distilled water (200 mL), shaken by a rotary shaker for 3h, and left to be settled for ½ h. For the fungal strain isolation, the supernatant (500 µL) was transferred to a sterile slant containing sterilized water (5 mL) which was further used for serial dilution preparation (10^–1^ to 10^–5)^, followed by inoculation of each dilution in potato dextrose agar plates (PDA, Merck, Darmstadt, Germany) (Idris et al. 2013) having neomycin (125 mg/L) to minimize the growth of bacteria. After that, incubation of the plates for 6–8 days at 30 ± 2 °C, and the fungal growth was mentioned after 48 h, Moreover, the growing colonies with unalike morphological character were designated and relocated to new PDA media at 40°C, subsequently, repeated and sequential sub-culturing was carried out for isolation of pure strains.

### Identification of the pure strains

Identification of the fungal strains was achieved as mentioned before in our previous work (Fayek et al. 2022). ITS-rDNA sequence was used for matching examination by the Blast N algorithm alongside the database for the National Centre of Biotechnology Information (NCBI; http://www.ncbi.nlm.nih.gov). The collective results and the phylogenetic tree were made using Molecular Evolutionary Genetics Analysis (MEGA) version 10.0.5 (https://www.megasoftware.net/).

### *Aspergillus terreus* cultivation

It was carried out by taking fungal biomass from PDA to fresh solid rice media present in Erlenmeyer flasks (10 × 1L) followed by incubation for 14 days at 30°C under static conditions.

### Tentative identification of *A. terreus* metabolites using HPLC / ESI–MS

Positive and negative modes ESI–MS were applied to detect the metabolites in the fungal ethyl acetate extract. Additional file 1: Figure S1-S3 and Table S1.

### Isolation of compounds from *A. terreus* extract

The rice culture media of the purified *A. terreus* was extracted by ethyl acetate till exhaustion (3 × 250 mL), filtered then evaporated the solvent under vacuum to yield 9.0 g dry residue. About 100 g of silica gel was mixed with 8.0 g of the extract dissolved in a mixture of dichloromethane (DCM) /MeOH (90:10 v/v, 50 mL) and then dried at 60 °C under *vacuum*. The dried mixture was subjected to fractionation using silica gel 60 vacuum liquid chromatography (VLC) (600 g, 13 × 25 cm), and eluted with DCM: MeOH (100:0:100%). Ten fractions (1.0 L) were eluted and further grouped into five main fractions depending on their behavior towards UV-light and *p*-anisaldehyde/sulfuric acid reagent on TLC. The first fraction (2.0 g) eluted with DCM (100%) was subjected to silica gel CC using *n*-hexane: DCM (30:70 v/v) for elution to yield a pure sample of **1** (200 mg). Fractionation of the second fraction II (DCM: MeOH, 95:5 v/v, 1.5 g) using silica gel CC and *n*-hexane: ethyl acetate (60:40 v/v) as eluent to yield three subfractions. Two of which (i) and (ii) contain a crude sample of compounds **2** and **3** respectively. A pure sample of **2** (12 mg) was obtained through fractionation on silica gel CC and *n*-hexane: ethyl acetate (70:30 v/v) as eluent, while pure sample **3** (9 mg) was obtained by silica gel CC and *n*-hexane: ethyl acetate (60:40 v/v) as eluent. The fourth fraction (59.0 mg) was subjected to successive silica gel CC and DCM: MeOH (94:06 v/v) for elution yielding a pure sample of **4 (**15 mg**)**. The isolated samples′ purity was checked using TLC and HPLC techniques.

*Compound 1:* Colorless oil, its ^1^H and ^13^CNMR data (CDCl_3_, 400, and 100 MHz) is represented in Table 1. Negative ESI/MS: *m/z* 655.5457 [M-H] ^−^. Spectral data of compound **1** are represented in Additional file 1: Figures S4-S10.

**Table 1:** ^1^H and ^13^C-NMR data of compound 1 (CDCl_3_, 400 MHz and 100 MHz)

C No.	δ C (ppm)	δ H (ppm)	^1^H-^1^H COSY	HMBC
	1	1		
**1**_**a**_** 1**_**b**_	61.9 CH_2_	4.24 (dd, 4.4, 12.0); 4.07 (dd, 6.0, 12.0)	H-2; H-2	C-2, C-3, C-1′; C-2, C-3, C-1′
**1′**	172.9 C			
**2′**	33.9 CH_2_	2.25 (td, 7.6, 0.2)	H-3′	C-1′, C-3′
**3′**	24.7 CH_2_	1.53 (m)	H-2′, H-4′	C-1′, C-4′
**4′**	29.6 CH_2_	1.19–1.24 (m)	H-3′, H-5′	
**5′**	27.1 CH_2_	1.96 (m)	H-4′, H-6′	C-4′, C-7′
**6′**	129.8 CH	5.26–5.28 (m)	H-5′	C-5′
**7′**	129.7 CH	5.26–5.28 (m)	H-8′	C-5′
**8′**	25.5 CH_2_	2.69 (t like, 6.4)	H-7′, H-9′	C-10′, C-7′
**9′**	127.8 CH	5.26–5.28 (m)	H-8′	C-8′
**10′**	129.5 CH	5.26–5.28 (m)		C-8′
**11′**	31.8 CH_2_	1.19–1.24 (m)	H-12′	
**12′**	22.6 CH_2_	1.19–1.24 (m)	H-13′, H-11′	
**13′**	14.0 CH_3_	0.80 (m)	H-12′	C-12′, C-11′
**2**	68.9 CH	5.19 (m)	H-1_a_, 1_b_, 3_a_, 3_b_	C-1, C-3, C-1″
**1″**	172.5 C			
**2″**	34.0 CH_2_	2.23 (td, 7.6, 0.2)	H-3″	C-1″, C-3″
**3″**	24.7 CH_2_	1.53 (m)	H-2″, H-4″	C-1″, C-4″
**4″**	29.0 CH_2_	1.19–1.24 (m)	H-3″, H-5″	
**5″**	27.1 CH_2_	1.96 (m)	H-4″, H-6″	C-4″, C-7″
**6″**	129.9 CH	5.26–5.28 (m)	H-5″	C-5″, C-8″
**7″**	129.8 CH	5.26–5.28 (m)	H-8″	C-5″, C-8″
**8″**	27.1 CH_2_	1.96 (m)	H-7″, H-9″	C-7″
**9″**	29.3 CH_2_	1.19–1.24 (m)	H-8″	
**10″**	31.5 CH_2_	1.19–1.24 (m)	H-11″	
**11″**	22.5 CH_2_	1.19–1.24 (m)	H-12″, H-10″	
**12″**	13.9 CH_3_	0.83 (m)	H-11″	C-10″, C-11″
**3**_**a**_** 3**_**b**_	61.9 CH_2_	4.24 (dd, 4.0, 11.6); 4.07 (dd, 6.0, 12.0)	H-2 H-2	C-1, C-2, C-1‴ C-1, C-2, C-1‴
**1‴**	172.9 C			
**2‴**	33.9 CH_2_	2.25 (td, 7.6, 0.2)	H-3‴	C-1‴, C-3‴
**3‴**	24.7 CH_2_	1.53 (m)	H-2‴, H-4‴	C-1‴, C-4‴
**4‴**	29.6 CH_2_	1.19–1.24 (m)	H-3‴, H-5‴	
**5‴**	27.1 CH_2_	1.96 (m)	H-4‴, H-6‴	C-4‴, C-7‴
**6‴**	129.8 CH	5.26–5.28 (m)	H-5‴	C-5‴
**7‴**	129.7 CH	5.26–5.28 (m)	H-8‴	C-5‴
**8‴**	25.5 CH_2_	2.69 (t like, 6.4)	H-7‴, H-9‴	C-7‴, C-10‴
**9‴**	127.9 CH	5.26–5.28 (m)	H-8‴	C-8‴
**10‴**	129.5 CH	5.26–5.28 (m)		C-8‴
**11‴**	31.8 CH_2_	1.19–1.24 (m)	H-12‴	
**12‴**	22.6 CH_2_	1.19–1.24 (m)	H-13‴, H-11‴	
**13‴**	14.0 CH_3_	0.80 (m)	H-12‴	C-11‴, C-12‴

Values between parentheses represent *J*- values in Hz

*Compounds 2 and 3:* Isolated as a faint yellow amorphous powder. Negative ESI/MS of **2** at *m/z* 423.2239 [M-H] ˉand 847.4105 for [2M-H**]**^**−**^, while that of **3** at 439.2230 and 879.4200 for [M-H**]**^**−**^ and [2M-H**]**^**−**^, respectively. Their ^1^HNMR, ^13^CNMR (500, 125 MHz; CD_3_OD) and ESI–MS spectra are represented in Additional file 1: Figure S11-S24 and Table S2-S3.

*Compound 4:* White amorphous powder; + ve ESI/MS at *m/z*. 563.6636 [M + H]^+^. 1 and 2-D NMR data (500 MHz; CD_3_OD) were represented in Table 2. Moreover, and ESI–MS spectra and ^1^HNMR, ^13^CNMR (500 MHz; CD_3_OD) Additional file 1: Figure S25—S31.

**Table 2:** ^1^H and ^13^C-NMR data for compound 4 (CD_3_OD*,* 500 MHz and 125 MHz)

C No	δ C (ppm)	δ H (ppm)	^1^H-^1^H COSY	HMBC
	4	4		
**2**	171.3 C			
**3**_**a**_** 3**_**b**_	44.3 CH_2_	3.94 (d, 15.0) 3.63 (d, 15.0)	H-3_a_ H-3_b_	C-5 C-5
**5**	172.7 C			
**6**	54.2 CH	4.93 (m)	H-7	C-5, C-8
**7**_**a**_ **7**_**b**_	26.2 CH_2_	3.28 (m) 3.49 (dd, 15.0, 4.0)	H-6, H-7_b_ H-6, H-7_a_	C-5, C-6, C-8, C-8_a_, C-14, C-1″ C-8, C-8_a_, C-6
**8**	109.7 C			
**8**_**a**_	127.4 C			
**9**	118.2 CH	7.61 (dd, 8.5, br.s)	H-10	C-8, C-8_a_, C-11, C-12_a_
**10**	118.7 CH	7.03 (m)	H-9	C-8_a_, C-12
**11**	121.4 CH	7.09 (m)	H-12	C-9, C-12_a_
**12**	111.2 CH	7.34 (dd, 8.5, br.s)	H-11	C-8_a_, C-10
**12**_**a**_	136.9 C			
**14**	123.4 CH	7.16 (s)		C-7, C-8, C-8_a_, C-12_a_
**2′**	171.5 C			
**3′**_**a**_** 3′**_**b**_	43.2 CH_2_	4.38 (d, 17.0) 3.44 (d, 17.0)	H-3′_b_ H-3′_a_	C-2′, C-4′ C-2′, C-4′
**4′**	167.9 C			
**5′**	170.7 C			
**5′**_**a**_	122.9 C			
**6′**	114.2 CH	6.99 (d, 3.0)		C-5′, C-7′, C-8′, C-9′_a_
**7′**	153.5 C			
**8′**	118.6 CH	6.90 (dd, 9.0, 2.5)	H-9′	C-6′, C-7′, C-9′_a_
**9′**	122.0 CH	8.19 (d, 9.0)	H-8′	C-5′_a_, C-7′
**9′**_**a**_	129.3 C			
**10′**_**c**_** 10′**_**d**_	27.4 CH_2_	1.88 (m), 2.24 (m)	H-10′_d_,10′_a_ H-10′_c_	C-2′, C-10′_a_, C-1′′ C-10′_a_, C-6′, C-9′_a_, C-1′′
**10′**_**a**_	51.4 CH	4.81 (dd, 8.5, 3.5)	H-10′_c_	C-2′
**1′′**	119.7 C			
**2′′**	12.8 CH_3_	2.13 (s)		C-1′′

Values between parentheses represent *J*- values in Hz

## Biological evaluation

### In vitro assessment of ACE2: Spike RBD (SARS-CoV-2) inhibiton

For assessment of the inhibitory action of *A. terreus* extract and the pure compounds (**1–4**) against the ACE2 receptor, the ACE2: SARS-CoV-2 spike inhibitor screening colorimetric assay kit (BPS Bioscience, San Diego, CA 92121) was used (Hoffmann et al. 2020) and following the manufacturer′s instructions. In brief, SARS-CoV-2 Spike-Fc was incubated with ACE2 protein which was attached to a clear nickel-coated 96-well plate along with ethyl acetate extract and compounds (**1–4**) (5–50 µg/mL), then HRP-labeled anti-Fc and substrate were added. The result color was measured at λ450 nm, and all tests were repeated in triplicates. Quercetin was used as a positive control. Calculation of IC_50_ was done using a dose–response inhibitory curve using Windows Graph Pad Prism version 5 (GraphPad Inc., USA).

### In vitro cell-based anti-inflammatory activity

Murine macrophage cells (RAW 264.7), supplied by American Type Culture Collection (ATCC), were grown on Dulbecco′s Modified Eagle Medium (DMEM), accompanied with 10%, 100U/100 mL and 100 μg/mL from bovine serum, penicillin, and streptomycin respectively. After that, cells were incubated in a CO_2_ atmosphere (5%, 37 °C) then they sub-cultured every 2 days and the cells of the exponential phase were used all over the procedure.

### MTT cell viability

In vitro cytotoxicity assay kit MTT-based was purchased from Sigma- Aldrich, MTT cat No: M-5655, MTT solubilization solution: M-8910). RAW 264.7 cells (10,000/well) were plated in a 96-well plate then the test compounds (**1–4**) and the extract were diluted by a serial dilution technique (1000–4 μg/mL) were introduced and the plate was placed in a CO_2_ incubator (37 °C for 24 h). The cells were washed by DMEM and incubated with MTT solution (40 μL, 4 h). DMSO (180 μL) was used to solubilize the crystals of formazan and the produced color was measured at λ570 nm. The results were normalized against the control and the cell viability percentage was estimated as follows:

$$\%\boldsymbol{ }\text{Cell viability }= (\text{Absorbance of sample }/\text{Absorbance of control})\mathrm{ x }100.$$

A quarter of this concentration (¼ IC_50_) was used to investigate the inflammatory marker in RAW 264.7 macrophages.

### Lipopolysaccharide-induced inflammatory model

RAW 264.7 cells (1.8 × 10^5^ cells/mL) were seeded for 18 h in a 24-well plate having DMEM medium (1 mL). When the cells reached confluence, they were treated with concentrations equivalent to ¼ IC_50_ of the selected samples (**1–4** and extract) for 2 h. Then, LPS (Sigma-Aldrich Cat No: L2630) (500 ng/mL, LPS control) was added to all treated and untreated wells to induce the inflammatory process. After incubation for 24 h, the media were taken and frozen till analysis. The untreated wells represented the LPS control group.

### Determination of IL-6 and TNF- α levels using ELISA

TNF- α quantitative assay ELISA kit (Abcam, ab181421 Human TNF alpha Simple Step) and Il-6 quantitative assay ELISA kit (Abcam, Ab178013 Human IL-6 Simple Step) were used to quantify IL-6 and TNF- α levels in the cell culture medium following the manufacturer′s instructions (Afshari et al. 2005).

### RT-PCR test

RAW 264.7 cells were washed two times and then harvested in PBS (1 mL). Total RNA from the stimulated RAW 264.7 cells was extracted utilizing Qiagen RNA extraction (Qiagen, MD, USA) following the instructions of the procedure. cDNA was synthesized by iScript cDNA synthesis kit (Bio-Rad Laboratories Pty. Ltd, Gladesville, Australia). Rotorgene RT-PCR system (QIAGEN, Germany) was used for PCR amplification using BioRad cyber green PCR MMX (Bio-Rad Laboratories Pty. Ltd, Gladesville, Australia). The primers used for the amplification of IL-6, TNF-α, and *β*-actin genes are represented in Additional file 1: Table S4.

The Formula 2^−(ΔΔCt)^ was used to calculate the relative fold change between samples and control.

### Antimicrobial evaluation

*Staphylococcus aureus* ATCC 6538**,** *Escherichia coli* ATCC 25922, *Candida albicans* ATCC 10231and *Aspergillus niger* NRRL-A326 were supplied from Culture Collection Center. The antimicrobial activity of *A. terreus* extract and **1–4** was investigated by the agar disc diffusion method **(**Abdel-Wareth et al. 2019**)**. Nutrient agar (bacteria and yeast) and PDA (fungi) plates were seeded with 0.1 mL of 10^5^–10^6^ cells/mL then the filter paper discs (0.5 cm) were loaded with about 0.01mg from the extract and compounds, and put on the inoculated agar or PDA plates surface and stayed for 2–4 h at 4°C to reach maximum diffusion. After that, incubation of the plate at 30°C for 24 and 48 h for bacteria and fungi, respectively, to permit maximum growth then the inhibition zone diameter was measured in millimeters (mm). Experiments were carried out in triplicate.

### Evaluation of minimum inhibitory concentration (MICs) and minimum bactericidal (MBCs) evaluation

The tested strains were cultivated in 100 mL bottles at 35 °C for 24 h on Mueller Hinton medium (Sigma-Aldrich, Germany). Bacteria and fungi cells (pellet) were collected by centrifugation at 5000 rpm, 4 °C, and under aseptic conditions, then washed with sterile saline till the supernatant became clear. Cell suspension has been performed to achieve an optical density of 0.5 to 1 (at 550 nm) yielding actual colony-forming units of 5 × 10^6^ cfu/ml. 50µL of broth medium was added to all wells of 96 well sterile microplates, then 50 µL of the tested samples (2 mg/mL MeOH for compounds and 10 mg/mL MeOH for extract as stock concentration) was pipetted into the first well and then two-fold serial dilutions were done. 10µL of resazurin indicator (Sigma-Aldrich, Germany; 270 mg/40 mL sterile distilled water) was added to each well followed by 10µL of bacterial and fungi suspension. Duplicate plates were prepared and incubated (37 °C, 18–24 h). The lowest concentration at which the color is changed is the MIC data, while MBC has been calculated by streaking two concentrations higher than MIC showing no growth (Sarker et al. 2007).

### Statistical analysis

The data were obtained from three individual experiments and the statistical significance was established by GraphPad Prism version 5 for Windows (Graph Pad Inc., USA) by one-way variance analysis (ANOVA). P < 0.05 is considered as statistically significant.

## Results

### Molecular identification of the *Aspergillus terrus*

**Fig. 1:**
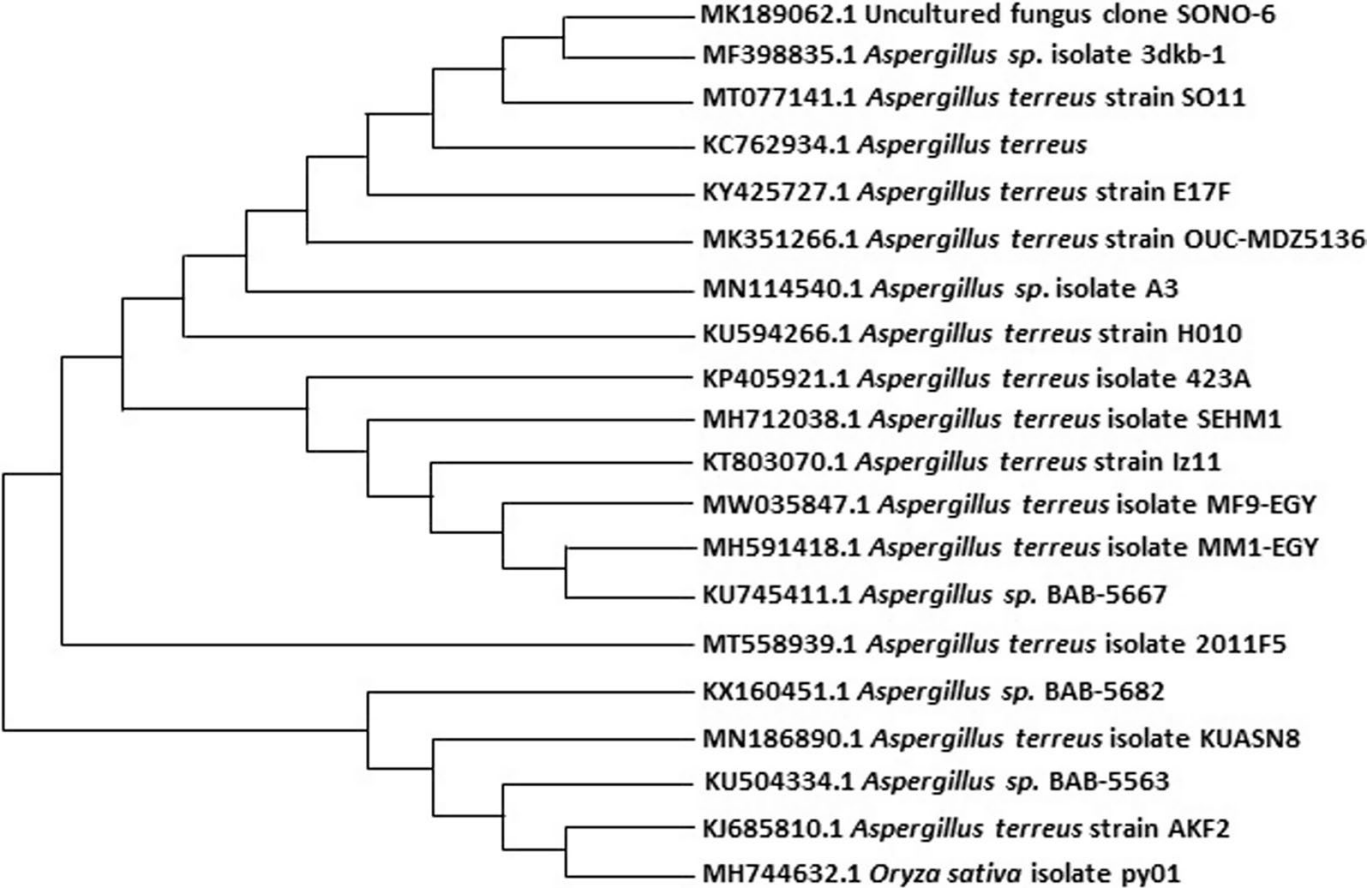
Phylogenetic tree showing relationship of strain *A. terreus* with other related fungal species retrieved from GenBank based on their sequence homologies of 18srDNA

In the current study, a filamentous fungus with long, colorless conidiophores was obtained from a soil sample. Moreover, after a comparison search of the fungus 18SrRNA nucleotide sequence by the NCBI database, the results revealed that there is a 100% similarity between the sequence and *Aspergillus terreus*. Data were represented in GenBank under the accession number MW035847 and its phylogenic tree is represented in Fig. 1. As well as it preserved in the Egypt Microbiological Culture Collection (EMCC) with an EMCC number 28559 on 29-3-2023 (Additional file 1: Figure S32).

### HPLC/ESI–MS tentative identification for *A. terreus* metabolites

HPLC/ESI—MS for *A. terreus* ethyl acetate extract revealed the tentative identification of **forty-four** metabolites depending on the comparison of their molecular ion peaks with the previously reported data for different *Aspergillus* species (Additional file 1: Table S1 and Figure S3). The identified compounds were related to different natural products classes viz alkaloids (**5, 12–16, 39 and 44**), sesteterrpenoids **(1, 37),** meroterpenoids **(2, 17–19, 25, 31–36, 38, 40–43),** polyketides **(3),** γ-butyrolactones **(4, 7–10, 20–24, 26–27),** peptides **(28–29),** prenylated phenol derivative **(11),** phenolic compound **(6).**

### Compounds identification from* A. terreus* extract

**Fig. 2:**
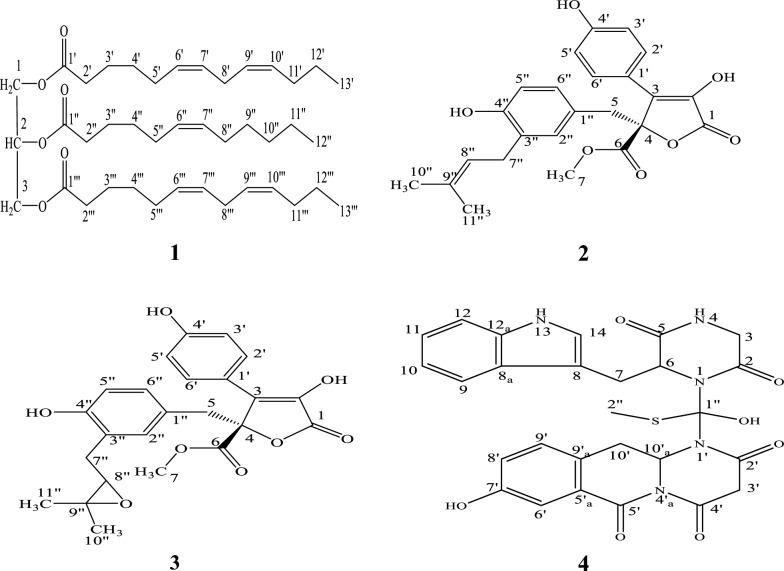
Chemical structure of compounds 1–4 isolated from *A. terreus*

The main compounds were isolated by VLC followed by successive silica gel columns eluted with different solvent systems to give four pure compounds **1–4** (Fig. 2). Their structures were established using different spectroscopic techniques.

#### Compound 1

**Fig. 3:**
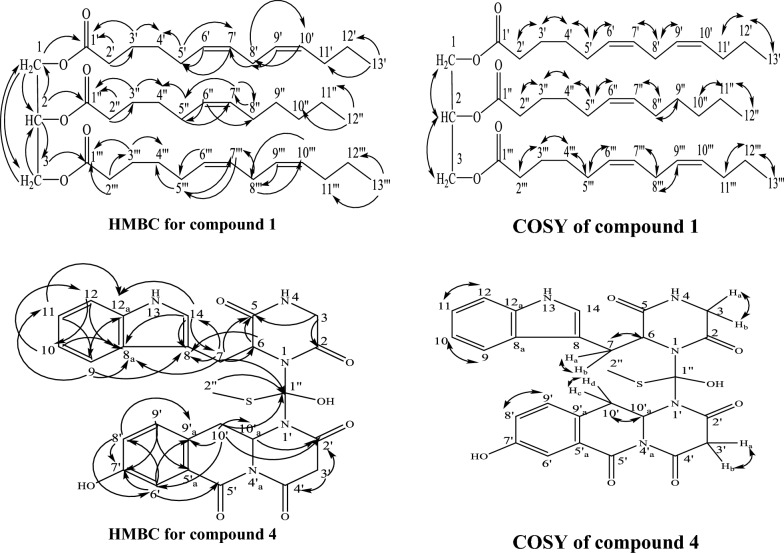
HMBC and COSY correlations in compounds 1 and 4

Negative ESI/MS [M-H]^−^ at *m/z* 655.5457 of **1** together with ^13^CNMR, APT, and HSQC data proposed that its molecular formula is C_41_H_68_O_6_, with eight degrees of unsaturation. ^13^CNMR data showed three carbon signals at δ_C_ 172.9 (C-1′, C-1‴) and 172. 5 (C-1′′) indicating the presence of three ester carbonyl carbons (Swaroop et al. 2005; Vlahov 1999). Besides, the occurrence of the proton signals in ^1^HNMR spectrum at δ_H_ 4.24 (2H, dd, H-1_a_, and H-3_a_), 4.07 (2H, dd, H-1_b_, and H-3_b_) and 5.19 (1H, m, H-2) corresponding to carbon signals at δ_C_ 61.9 (C-1, C-3) and 68.9 (C-2) which established that **1** is a related to triglyceride with C_13_ and C_12_ fatty acid esters (Ramsewak et al. 2001), which further established by ^1^H-^1^H-COSY (Fig. 3) that displayed the correlation between H-1_a_, H-1_b_ and H-3_a_, H-3_b_ (δ_H_ 4.24, 4.07) with H-2 (δ_H_ 5.19). In addition, HMBC spectrum (Fig. 3) display correlation from H-2′′ (δ_H_ 2.23) with carbonyl carbon C-1′′ (δ_C_ 172.5) and that between H-2′, H-2′″ (δ_H_ 2.25) with carbonyl carbon C-1′, C-1‴ (δ_C_ 172.9), also that between H-3′, H-3″ and H-3″′ (δ_H_ 1.53) and C-1′, C-1′′ (δ_C_ 172.9), C-1′′ (δ_C_ 172.5), along with the HMBC correlation between H-1_a_, H-1_b_ (δ_H_ 4.24, 4.07) with C-2 (δ_C_ 68.9), C-3 (δ_C_ 61.9) and C-1′ (δ_C_ 172.9), H-3_a_, H-3_b_ (δ_H_ 4.24, 4.07) with C-2 (δ_C_ 68.9), C-1 (δ_C_ 61.9) C-1′″ (δ_C_ 172.9) and that from H-2 (δ_H_ 5.19) with C-1, C-3 (δ_C_ 61.9) and C-1″ (δ_C_ 172.5), which also established that **1** is a long chain fatty acid ester triglyceride. Furthermore, the ^1^HNMR spectrum displayed multiplet proton signals at δ_H_ 5.26 -5.28 for H-6′, H-7′, H-9′, H-10′, H- 6″, H- 7″, H- 6‴, H-7″′, H-9″′ and H-10″′ which correlated in the HSQC data with olefinic carbon signals at δ_C_ 127.8–129.9, which indicated the presence of five olefinic double bonds in the structure of **1**. As well as, in the HMBC the correlation from H-6′, H- 6″′, H-7′ and H-7′″ (δ_H_ 5.26–5.28) with C-5′, H-5″′ (δ_C_ 27.1), also that between H-9′, H-10′ and H-9″′ and H-10″′ with C-8′ and C-8″′ (δ_C_ 31.8). Moreover, the correlation between H-8′ and H-8′″ (δ_H_ 2.69) with C-10′ and C-10′″ (δ_C_ 129.5) and C-7′ and C-7″′ (δ_C_ 129.7). In addition, there is a correlation from H-6″ and H-7″ to C-5″ and C-8″ (δ_C_ 27.1) which support the presence of five olefinic double bonds in the structure. Moreover, the ^1^HNMR spectrum showed characteristic two signals at δ_H_ 0.83 and 0.80 for the terminal methyl groups H-12′′ and H-13′, H-13‴ respectively. This suggestion was confirmed by the correlation between H-12′′ (δ_H_ 0.83) with H-11′′ (δ_H_1.19–1.24) in ^1^H-^1^H-COSY and that between H-12″ and C-10′′ (δ_C_ 31.5) and C-11′′ (δ_C_ 22.5) in HMBC and from H-13′, H-13‴ (δ_H_ 0.80) with H-12′, H-12‴ (δ_H_1.19–1.24) and C-12′, C-12‴ (δ_C_ 22.5) and C-11′, C-11‴ (δ_C_ 31.8) in ^1^H-^1^H-COSY and HMBC respectively. Furthermore, two methylene triplet doublets at δ_H_ 2.23 (H-2″) and 2.25 (H-2′, 2″′) and multiplet methylene signals at δ_H_ 1.53 for H-3′, H-3″ and H-3′″, 1.96 for H-5′, H-5″ and H-5′″, 1.19 for H-4′, H-4″ and H-4″′, in addition to characteristic triplet methylene signal at δ_H_ 2.69 (H-8′/H-8‴) were detected in the ^1^HNMR. This suggestion was confirmed from ^1^H-^1^H-COSY connection between H-2′′ and H-2′, H-2‴ with H-3′′ and H-3′, H-3‴, which further correlated with H-4′′ and H-4′, H-4‴ respectively. Moreover, H-5′, H-5′′ and H-5‴ is correlated to H-4′, H-4′′ and H-4‴ and H-6′, H-6′′ and H-6‴ respectively as well as the correlation from H-8′/8‴ (δ_H_ 2.69) to H-7′/7‴ and H-9′/9‴. In addition, the HMBC spectrum displayed a correlation from H-3′, and H-3‴ (δ_H_ 1.53) to C-1′, C-1‴ (δ_C_ 172.9), and C-4′, C-4″′ (δ_C_ 29.6) also that from H-3″ (δ_H_ 1.53) to C-1″ (δ_C_ 172.5) and C-4″ (δ_C_ 29.0). Moreover, H-5′, H-5′″ is correlated to C-4′, C-4′″ (δ_C_ 29.6) and C-7′, C-7″′ (δ_C_ 129.7) and H-5″ is correlated to C-4″, (δ_C_ 29.0) and C-7″ (δ_C_ 129.8) as well as H-8′/8″′ (δ_H_ 2.69) is correlated to C-10′/10″′ (δ_C_ 129.5) and C-7′/7″′ (δ_C_ 129.7). The remaining ^1^H-^1^H-COSY and HMBC correlations were deposited in Table 1 as well as, ^13^C NMR, HSQC, and APT spectra confirmed the direct correlation of the proton signals with the corresponding carbon signals. The presence of only two carbon signal characteristics for three ester carbonyls at δ_C_ 172.5 (C-1″), 172.9 (C-1′, C-1′″) supported the evidence that the structure of **1** is a symmetrical triglyceride (Swaroop et al. 2005; Vlahov 1999; Shiao and Shiao 1989). Therefore, compound **1** was identified as 1,3-di-(6*Z*,9*Z*)- trideca-6,9-dienoyl-2-(6*Z*) dodec-6-enoyl glycerol. To our knowledge, it was identified for the first time from nature.

#### Compounds 2–3

They were identified as butyrolactone I (**2**) and butyrolactone III (**3**) after comparison with the previously reported data (Uras et al. 2021).

#### Compound 4

The molecular formula of **4** was expected to be C_27_H_24_O_7_N_5_S from its positive ESI/MS which displays a molecular ion peak at *m/z* 563.6636 [M + H]^+^. The ^13^CNMR, APT, and HSQC spectra of **4** showed 27 carbons atoms including five carbonyls carbons at δ_C_ 167.9, 170.7, 171.3,171.5 and 172.7 for C-4′, C-5′, C-2, C-2′ -and C-5 respectively; four methylene *sp*^*3*^ at δ_C_ 44.3 (C-3), 43.2 (C-3′), 27.4 (C-10′) and 26.2 (C-7), six *sp*^*2*^ quaternary carbons at δ_C_ 153.5 (C-7′), 136.9 (C-12_a_), 129.3 (C-9′_a_), 127.4 (C-8_a_), 122.9 (C-5′_a_) and 109.7 (C-8) in addition to one *sp*^*3*^ quaternary at δ_C_ 119.7 (C-1′′). Eight methine *sp*^*2*^ at δ_C_ 123.4 (C-14), 122.0(C-9′), 121.4 (C-11), 118.7 (C-10), 118.6 (C-8′), 118.2 (C-9), 114.2 (C-6′), and 111.2 (C-12), and two methine *sp*^*3*^ at δ_C_ 54.2 and 51.4 for C-6 and C-10′_a_ respectively in addition to one thiomethyl carbon at δ_C_ 12.8 (C-2′′). ^1^HNMR data together with HSQC revealed that compound **4** has an indole moiety through the presence of four doublet protons in the aromatic region at δ_H_ 7.61, 7.34, 7.09, and 7.03 for H-9, H-12, H-11, and H-10, respectively, together one singlet signal at δ_H_ 7.16 for H-14. Moreover, this suggestion was confirmed by ^1^H-^1^H-COSY and HMBC data (Fig. 3), especially the correlation between H-14 (δ_H_ 7.16) and C-8 (δ_C_ 109.7), C-8_a_ (δ_C_ 127.4) and C-12_a_ (δ_C_ 136.9). Moreover, the HMBC correlation from H-3_a/b_ (δ_H_ 3.94 and 3.63) to C-5 (δ_C_ 172.7) and that from H-6 (δ_H_ 4.93) to C-5 (δ_C_ 172.7) gave the evidence for the presence of diketopiperazine skeleton. Moreover, the presence of correlation between CH_2_-7_a/b_ (δ_H_ 3.28, 3.49) to C-5 (δ_C_ 172.7) and C-6 (δ_C_ 54.2), C-8(δ_C_ 109.7), C-8_a_ (δ_C_ 127.4), C-14 (δ_C_ 123.4) support that the indole moiety is connected to the diketopiperazine moiety through methylene bridge CH_2_-7_a/b_. In addition, the ^1^HNMR and HSQC (Table 2) showed in the aromatic region another two doublet proton at δ_H_ 6.99 (δ_C_ 114.2) and δ_H_ 8.19 (δ_C_ 122.0) together with doublet doublets signal at δ_H_ 6.90 (δ_C_ 118.6) for H-6′, H-9′ and H-8′, respectively, which suggested the presence of trisubstituted benzene ring, which further supported from the correlations displayed in ^1^H-^1^H-COSY and HMBC (Table 2). In addition, there is another diketopiperazine moiety in the structure of **4** due to the occurrence of methylene group at δ_H_ 4.38 and 3.44 for H-3′_a/b_ and methine proton at δ_H_ 4.81 (H-10′_a_) as well as the correlation between H-3′_a/b_ and C-2′ (δ_C_ 171.5) and C-4′ (δ_C_ 167.9) and that from H-10′_a_ to C-2′ (δ_C_ 171.5) in the HMBC data supported the presence of the diketopiperazine moiety. Moreover, the HMBC correlations between H-10′_c/d_ (δ_H_ 1.88 and 2.24) with C-2′ (δ_C_ 171.5) and C-10′_a_ (δ_C_ 51.4) C-9′_a_ (δ_C_ 129.3) and C-6′ (δ_C_ 114.2) gave evidence for the connection of the trisubstituted benzene ring to the diketopiperazine moiety. Furthermore, the connection of the indole diketopiperazine part with the trisubstituted benzene ring- diketopiperazine part with each other was done by carbon atom bridge between N-1 and N-1′ which supported from HMBC correlation between H-10′_c/d_ (δ_H_ 1.88, 2.24) and H-7_a/b_ (3.28, 3.49) with C-1′′ (δ_C_ 119.7). Moreover, the thiomethyl group was established by the presence of a singlet signal at δ_H_ 2.13 (δ_C_ 12.8) as well as attached to C-1′′ (δ_C_ 119.7) through the correlation between them in the HMBC spectrum. So, **4** was identified as asterrine, which was purified based on our knowledge for the first time from nature.

## Biological activity

### Inhibitory effect of extract and compounds on ACE2 inhibition

Compounds (**1–4**) and the extract were tested for their ability to inhibit ACE2 to SARS-CoV-2 spike-protein receptor binding domains. The results revealed that all compounds have inhibitory activity with different potencies, but compounds **3** and **4** exhibit the most potent activity with IC_50_, 9.5, and 8.5 µg/mL, respectively (Table 3). Moreover, ethyl acetate extract showed a promising inhibitory activity with IC_50_, 7.4 µg/mL. Therefore, it would be expected that the extract and pure compounds could protect from viral infection by blocking this entry path for SARS-CoV-2.

**Table 3:** Inhibitory effect of *A. terreus* ethyl acetate extract and compounds (1–4) on ACE2/Spike (RBD) protein interaction

Compounds	IC_50_ ± SD (µg/mL)
Extract	7.4 ± 0.1
**1**	10.5 ± 0.1 (15.75 µM)
**2**	12.5 ± 0.1 (29.47 µM)
**3**	9.5 ± 0.2 (21.58 µM)
**4**	8.5 ± 0.2 (15.09 µM)
Quercetin	16.53 ± 0.44 µM

### In vitro cell-based anti-inflammatory activity

**Fig. 4:**
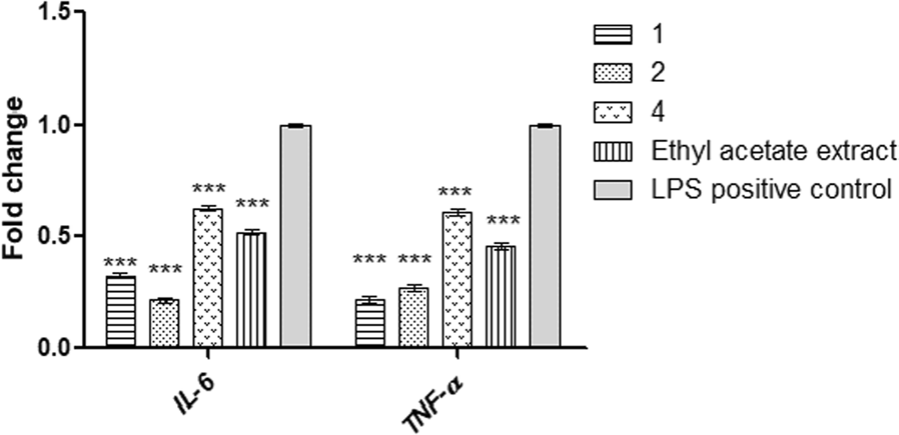
Effect of compounds (1, 2, 4) and ethyl acetate extract of *A. terreus* on the expression of IL-6 and TNF-α mRNA in RAW 264.7 cells. ***Significant from LPS control at p < 0.001

RAW 264.7 cell line induce lipopolysaccharides (LPS) was used for the evaluation the anti-inflammatory activity of the extract and pure compounds, since LPS encouraged cell imitator in vivo condition (Chao et al. 2010; Murakami et al. 2000). Initially, tested samples cytotoxicity on RAW 264.7 viability was assessed by MTT and the results revealed that ethyl acetate extract and compounds **1**–**4** exhibited IC_50_ equals to 1215, 858, 718, 246, and 449 μg/mL, respectively (Table 4). Therefore, the extract and the compounds **1**, **2**, and **4** are tested in triplicates at a dose equal to ¼ IC_50_ to evaluate the inflammation-related immune responses in LPS-induced RAW 264.7 macrophages and the anti-inflammatory activity against IL-6 or TNF-α in cell culture media was assessed by ELISA assay. LPS significantly encouraged IL-6 or TNF-α release in comparison to those of untreated cells. Interestingly, the finding results showed that the extract and tested compounds exhibit a significant inhibitory effect on both IL-6 and TNF-α (Table 4). Moreover, compounds **1** and **2** showed potent inhibitory activity with IC_50_ being 51.31 and 37.25 pg/mL (Il-6) and 87.97, 68.22 pg/mL (TNF-α) respectively in comparison to LPS control (Fig. 4).

**Table 4:** Cytotoxic effect of the ethyl acetate extract of *A. terreus* and pure compounds (1–4) on RAW 264.7 cells and anti-inflammatory activity of the ethyl acetate extract of *A. terreus* and pure compounds (1–2, 4)

Tested samples	IC_50_ (µg/mL) mean ± SD	IL-6 (pg/mL), mean ± SD	TNF-α (pg/mL) mean ± SD
Ethyl acetate extract	1215 ± 68.2	88.35 ± 0.92***	122.50 ± 3.98***
1	858 .0 ± 48.2 (1287.3 µM)	51.63 ± 2.79***	68.220 ± 3.09***
2	718.7 ± 40.4 (1694.4 µM)	37.25 ± 0.87***	87.97 ± 6.7***
3	246.8 ± 13.9 (560.7 µM)	–	–
4	449.0 ± 25.2 (797.3 µM)	129.00 ± 1.77***	268.30 ± 3.43***
LPS control		227.50 ± 29.2	452.5 0 ± 46.1

***Significant from LPS control at *p* < 0.001

### Effect of the extract and compounds 1, 2, 4 on gene expression of the inflammatory mediators

Furthermore, the anti-inflammatory effect of the investigated samples via IL-6 and TNF-α inhibition on the expression of mRNAs and their protein in the LPS-induced inflammatory model was assessed. The results revealed that the expression levels of IL-6 and TNF-α and their proteins in the cells treated with the extract and tested compounds were significantly decreased compared to the LPS control. Compound **2** showed the best inhibitory activity against IL-6, while compound** 1** exhibited the most activity against TNF-α (Fig. 3).

## Antimicrobial activity

Table 5 shows the susceptibility of *S. aureus* and *C. albicans* to the ethyl acetate extract, compounds **2** and **4** with the zone of inhibition 19–25 and 11–22 mm for *S. aureus* and *C. albicans* respectively. Moreover, It was found that ethyl acetate extract and compound **4** displayed antimicrobial activity against *S. aureus* (MIC = 62.5 and MBC = 125 μg/mL) and *C. albicans* (MIC = 125 μg/mL), while compound** 4** displayed a moderate activity toward *S. aureus* **(**MIC = 125 and MBC = 250 μg/mL) and *C. albicans* (MIC of 250 μg/mL) (Table 6).

**Table 5:** Antimicrobial activity for ethyl acetate extract of *A. terreus* and compounds (1–4)

Tested samples	Zone of inhibition (mm)			
	*S. aureus*	*E. coli*	*C. albicans*	*A. niger*
Ethyl acetate extract	24	0	22	0
1	0	0	0	0
2	19	0	11	0
3	0	0	0	0
4	25	0	15	0
Neomycin	21	8	23	0
Cyclohexamide	0	0	0	29

**Table 6:** The minimum inhibitory concentrations (MICs), and minimum bactericidal concentrations (MBCs)

Tested samples	*S. aureus*		*C. albicans*	
	MIC (µg/mL)	MBC (µg/mL)	MIC (µg/mL)	MBC (µg/mL)
Ethyl acetate extract	62.5	125	125	500
2	250	250	250	250
4	125	250	250	500

## Discussion

The current study aimed to tentatively identify the secondary metabolites of *A. terreus* purified and identified from the soil using HPLC/MS, as well as, purify four compounds from its ethyl acetate aiming to find ACE2 inhibition, anti-inflammatory and antimicrobial agents from natural sources. *A. terreus* is rich in a wide variety of biologically active compounds including butyrolactone I (Ghfar et al. 2021; Hamed et al. 2020), alkaloids (El‐Hawary et al. 2021; Qi et al. 2020) polyketides (El‐Hawary et al. 2021; Deng et al. 2020.) and terpenoids (Uras et al. 2021; Girich et al. 2020). The presence of these compounds rationalize the significant importance of the *Aspergillus* genus both in the scientific and pharmaceutical industries levels (Zhang et al. 2018). SARS-CoV-2 is an extremely pathogenic virus and it has produced a pandemic of acute respiratory disease, coronavirus disease 2019 (COVID-19), which affects the human health and safety (Hu et al. 2020). The serious consequences of the COVID-19 pandemic, urge researchers to discover an appropriate means for inhibition of the viral virulence and invasion (Dalan et al. 2020). As a part of RAS, ACE2 produces vasodilator peptides Ang 1–7, which in turn counterbalance the pro-inflammatory, pro-coagulant, and vasoconstrictive outcomes of Ang II. (Abassi et al. 2020). ACE 2 is considered the main SARS-CoV-2 functional receptor which helps its attachment to human cells and consequently enhances its replication (Walls et al. 2020; Zhou et al. 2020). Coronaviruses use the spike glycoprotein (comprising spike monomer′s S1 and S2 subunit) on the membrane for binding to their cellular receptors. This binding activates a cascade of actions that result in fusion between cells and membranes of the virus for entry to the host cell. So, binding to the ACE2 receptor is a serious early step for entering SARS-Co-V into goal cells. Our result revealed that all compounds can inhibit the binding of ACE2 to SARS-CoV-2 spike-protein receptor but compound **3** (butyrolactone III) and the new compound **4,** which are related to alkaloids, exert the most inhibitory activity. It was reported that some alkaloids isolated from *Aspergillus* can prohibit SARS-CoV-2 entry inside the host cells via ACE2 as well as it was reported that butenolides can exhibit SARS-CoV-2 (Uras et al. 2021; Qia et al. 2018). In addition, since the pathology of COVID-19 disease was related to the hyperinflammatory response mentioned as a cytokine storm, which is an extreme increase in pro-inflammatory cytokines levels as IL-6, and TNF-α as well as, the nearby correlation between anti-TNF-α agents and ACE2 inhibition. Therefore, cell-based assays were adopted to assess the biological significance of pro-inflammatory cytokines enzyme inhibitors as they are more prognostic since the enzymes act in their vital physiological environment and any change at the expression levels could be simply noticed. However, it is critical to select the most acceptable cell line, which is notable by the expression of the targeted enzymes. Our result revealed that the extract and the compounds exert a significant anti-inflammatory effect and the most potent one is compound **2** as previously reported that butenolides can exhibit anti-inflammatory activity (Qia et al. 2018). Moreover, it was reported that the extract obtained from *A. terreus* showed an antimicrobial activity against different strains of microorganisms (Pinheiro et al. 2018). The biological activity of the extract may be due to the synergistic activity of the different compounds present in the extract. In conclusion, the extract and the compounds exhibited significant ACE2 inhibitory effects, anti-inflammatory and antimicrobial activities. Conversely, more research is recommended to establish the mechanism of action for ant-inflammatory and ACE2 inhibitory activities of the active compounds as well as, investigation of the compounds on a large number of microorganisms should be to find natural antimicrobial agents.

### Supplementary Information

**Additional file 1.** **Supporting data for chemical analysis of isolated metabolites**. Data enclose HPLC/MS spectra of the fungal extract, ^1^H and ^13^C NMR data of isolated compounds, chemical structures of tentatively identified metabolites, list of ESI data for tentatively identified metabolites, discussion of compound **2** and **3**, and list of supporting references.

## Data Availability

The original contributions presented in the study are included in the manuscript, and additional queries can be asked of the corresponding authors.
