# Supplementary material for: Bioactive metabolites identified from Aspergillus terreus derived from soil
Source: AMB Express. 2023 Oct 3;13:107. doi: 10.1186/s13568-023-01612-0 (PMC10547674; doi:10.1186/s13568-023-01612-0)
Supplement: Supplementary file 1 — Additional file 1. Supporting data for chemical analysis of isolated metabolites. Data enclose HPLC/MS spectra of the fungal extract, 1H and 13C NMR data of isolated compounds, chemical structures of tentatively identified metabolites, list of ESI data for tentatively identified metabolites, discussion of compound 2 and 3, and list of supporting references. [file 13568_2023_1612_MOESM1_ESM.pdf]

Supplementary materials

Bioactive metabolites identified from *Aspergillus terreus* derived from soil

Menna Fayek[1#], Hassan Y. Ebrahim[1#], Mohamed S. Abdel-Aziz[2], Heba T. Sharafeldin[3,] Fatma A. Moharram[1*]

[1] Pharmacognosy Department, Faculty of Pharmacy, Helwan University, Cairo, 11795, Egypt.
[2] Department of Microbial Chemistry Department, Genetic Engineering and Biotechnology Division, National Research Centre, Cairo 12622, Egypt.
[3] Biochemistry and Molecular Biology Department, Faculty of Pharmacy, Helwan University, Cairo, Egypt, 11795.

# These authors share first authorship

Corresponding author

Fatma A. Moharram

Email: Famoharram1@hotmail.com

Department of Pharmacognosy, Faculty of Pharmacy, Helwan University, Ein Helwan, Cairo, 11795, Egypt, Work Phone & Fax: +202-2554-160 cell: +202-01064747056, ORCID ID: 0000-0003-3680-7271

Center for DRUG DISCOVERY RESEARCH and DEVELOPMENT Page 2
Openlynx Report -
Sample: 517 Vial:1:A,7 ID:
File:D21 65 Date:05-Sep-2021 Time:16:45:47
Description:MFq

Printed: Wed Sep 08 16:31:35 2021

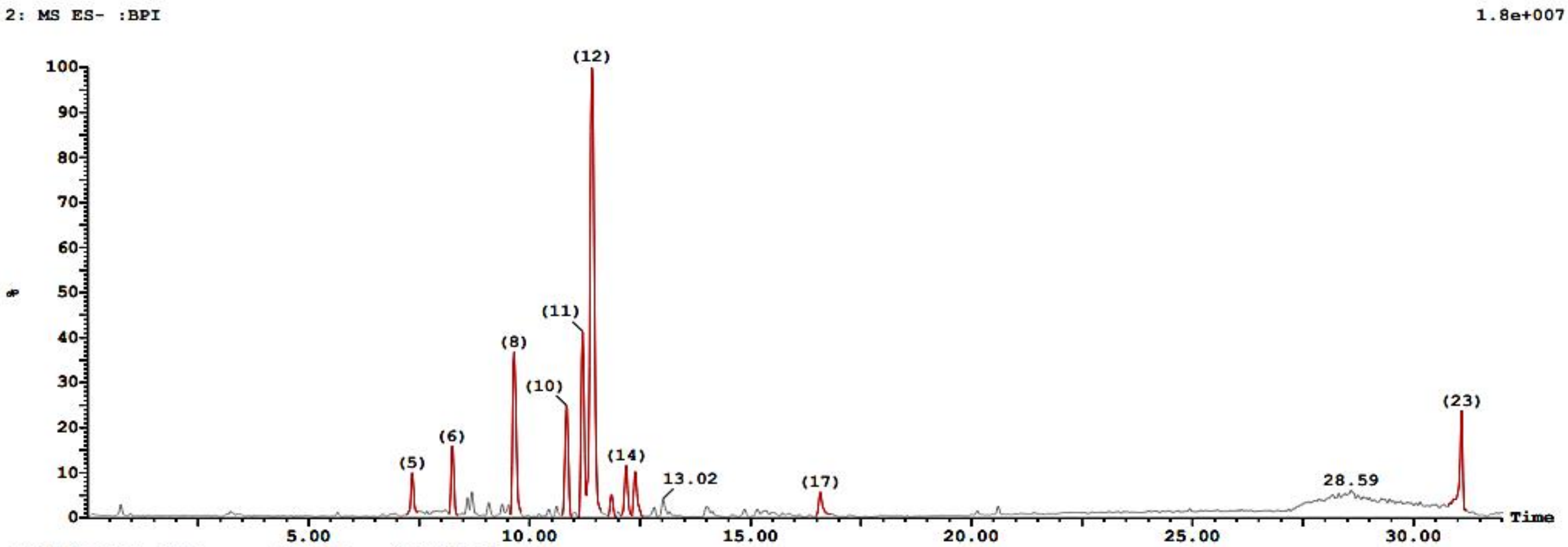

Peak Number	Time	AreaAbs	Area %Total
5	7.34	116549.3047	2.53
6	8.25	211362.0313	4.58
8	9.65	606791.0000	13.15
10	10.83	400409.3125	8.68
11	11.20	542228.3125	11.76
12	1.40	1899913.0000	41.19
13	11.86	66813.6875	1.45
14	12.19	173563.3750	3.76
15	12.39	161713.0625	3.51
17	16.58	92847.9609	2.01
23	31.09	340518.0938	7.38

Figure S1. Negative ESI-LC/MS chromatogram of the ethyl acetate extract

Center for DRUG DISCOVERY RESEARCH and DEVELOPMENT Page 1
Openlynx Report -
Sample: 517 Vial:1:A,7 ID:
File:D21 65 Date:05-Sep-2021 Time:16:45:47
Description:MFq

Printed: Wed Sep 08 16:31:35 2021

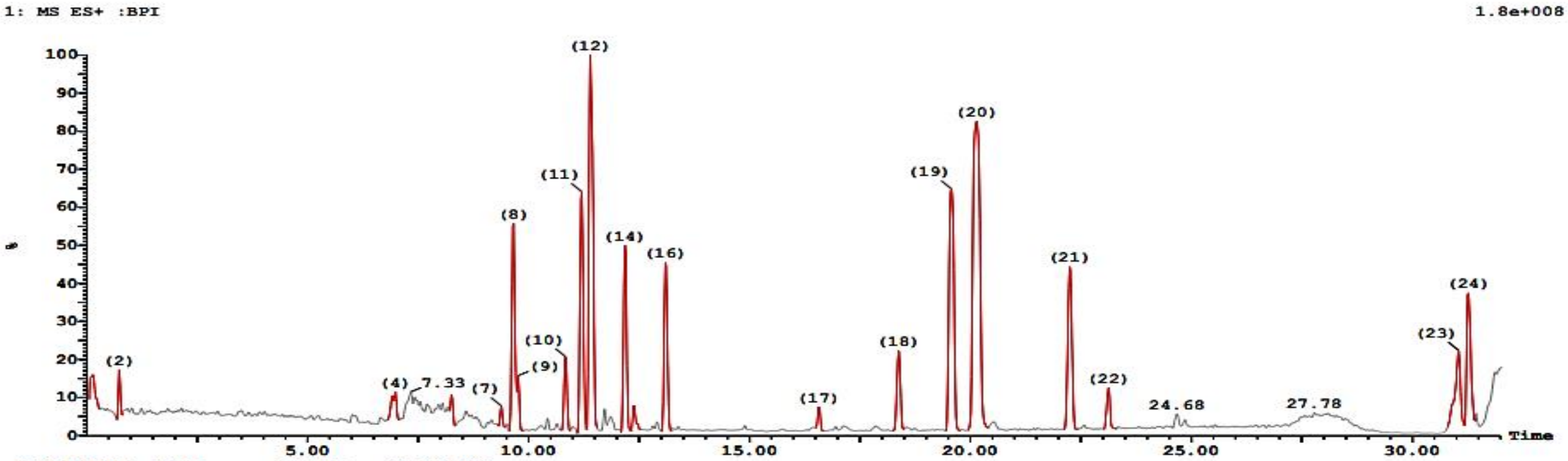

Peak Number	Time	AreaAbs	Area %Total
1	0.13	1525305.3750	1.23
2	0.74	1087715.5000	0.88
3	6.92	856225.6875	0.69
4	6.99	756703.5000	0.61
6	8.25	619458.7500	0.50
7	9.38	552532.8125	0.45
8	9.66	7841253.5000	6.33
9	9.76	1546404.2500	1.25
10	10.84	2630879.2500	2.12
11	11.21	9487039.0000	7.65
12	11.40	17809578.0000	14.37
14	12.18	6801319.5000	5.49
15	12.39	897998.6250	0.72
16	13.10	6499182.5000	5.24
17	16.57	714099.5000	0.58
18	18.37	3321332.7500	2.68
19	19.57	14198779.0000	11.46
20	20.15	25913782.0000	20.91
21	22.25	7995362.5000	6.45
22	23.13	1632843.5000	1.32
23	31.05	4981458.5000	4.02
24	31.27	6265681.0000	5.06

Figure S2. Positive ESI-LC/MS chromatogram of the ethyl acetate extract of *A. terreus*

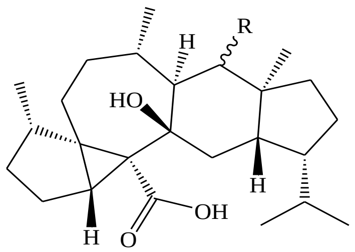

(1) R = OH; 1*R*
(40) R = $OCOCH_3$; 1*S*

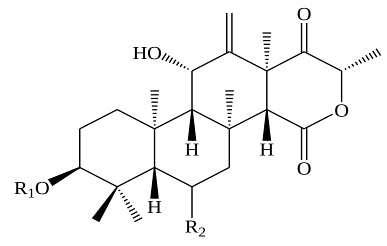

(2) $R_1 = R_2$ = H

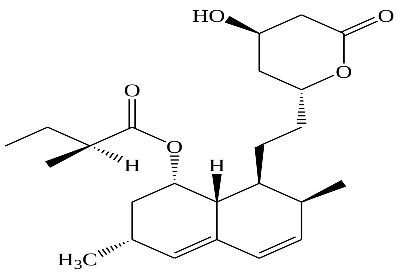

(3)

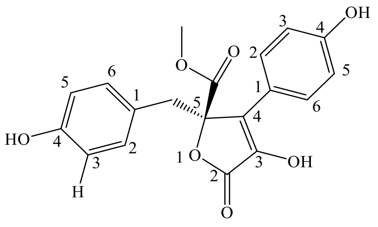

(4)

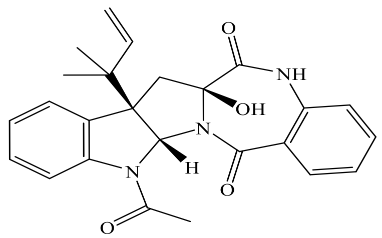

(5)

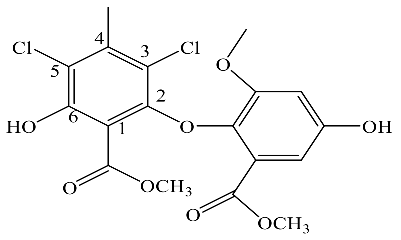

(6)

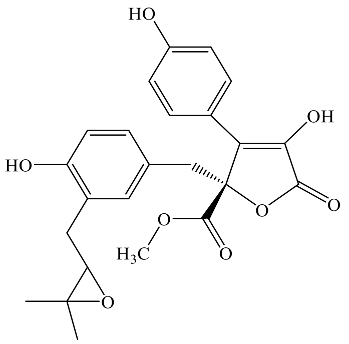

(7)

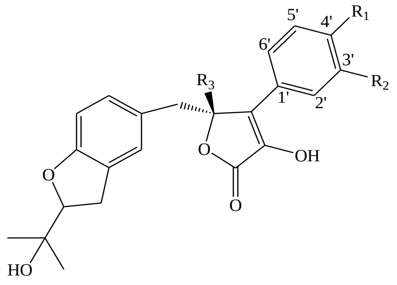

(8) R_1 = OH; R_2 = H; R_3 = $COOCH_3$
(23) $R_1 = R_2$ = H; R_3 = $COOCH_3$

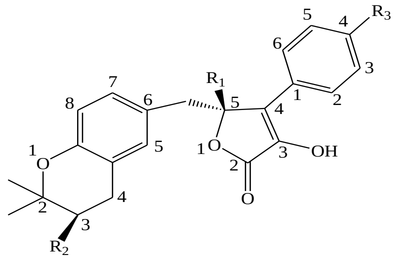

(9) R_1 = $COOCH_3$; $R_2 = R_3$ = OH
(24) R_1 = $COOCH_3$; R_2 = H; R_3 = OH
(25) R_1 = $COOCH_3$; R_2 = OH; R_3 = H

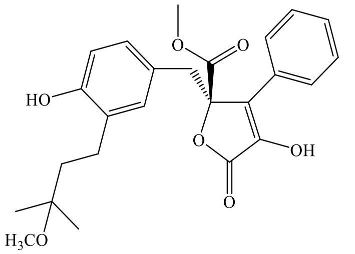

(10)

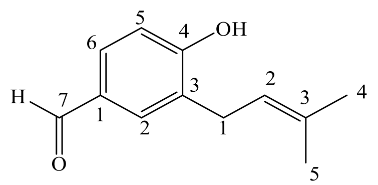

(11)

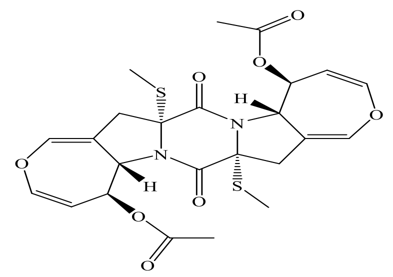

(12)

Figure S3. Structure for tentatively identified compounds in the ethyl acetate extract of *A. terreus* by HPLC/ESI/MS analysis

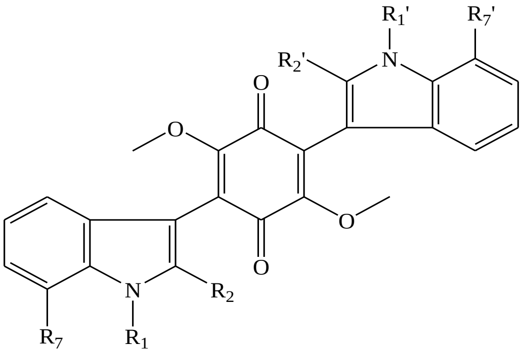

(13) $R_1 = R_1' =$ 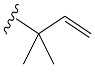 ; $R_2 = R_2' = R_7 = R_7' = H$

(14) $R_2 =$ 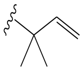 ; $R_7' =$ 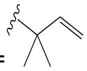 ; $R_1 = R_1' = R_2' = R_7 = H$

(15) $R_2 =$ 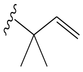 ; $R_7 =$ 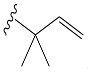 ; $R_1 = R_1' = R_2' = R_7' = H$

(16) $R_1 = R_2' =$ 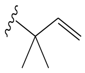 ; $R_1' = R_2 = R_7 = R_7' = H$

(17) $R_2 = R_2' =$ 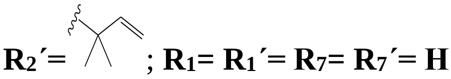 ; $R_1 = R_1' = R_7 = R_7' = H$

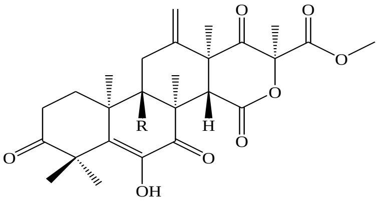

(18) R = OH
(39) R = H

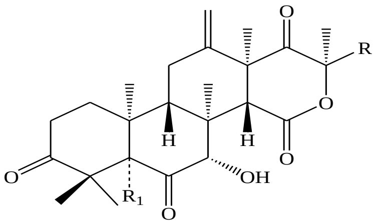

(19) R = $COOCH_2CH_3$; R_1 = *β*H
(37)R = $COOCH_3$; R_1 = *β*H

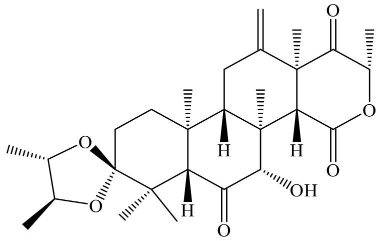

(20)

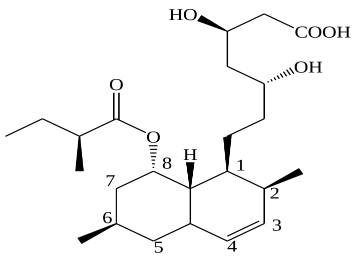

(21)

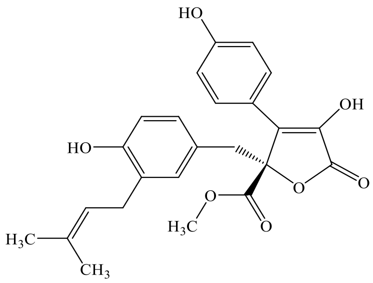

(22)

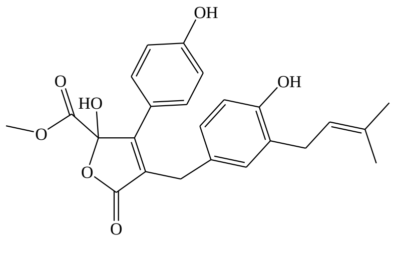

(26)

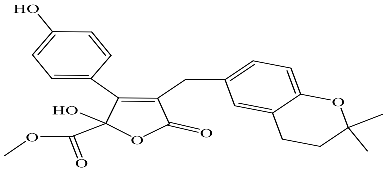

(27)

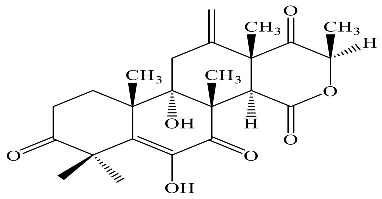

(28)

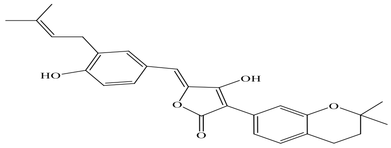

(29)

Figure S3 continue: Structure for tentatively identified compounds in the ethyl acetate extract of *A. terreus* by HPLC/ESI/MS analysis.

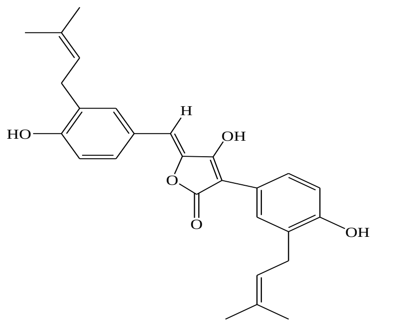

(30)

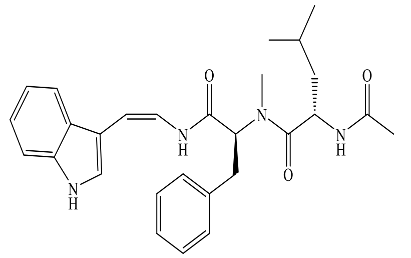

(31)

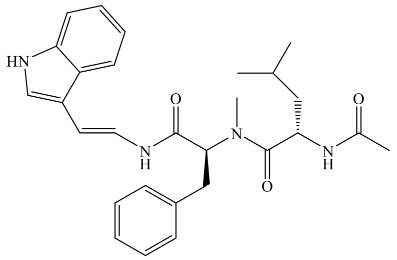

(32)

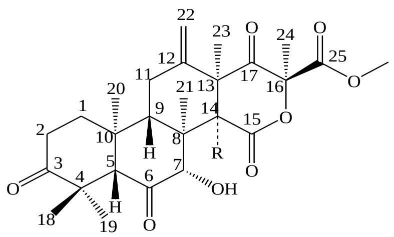

(33) R = *β*H-14
(34) R = *α*H-14

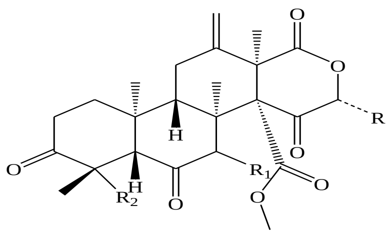

(35) R = R_2 = *α*CH_3; R_1 = OH
(36) R = *β*CH_3; R_1 = OH; R_2 = *α*CH_3

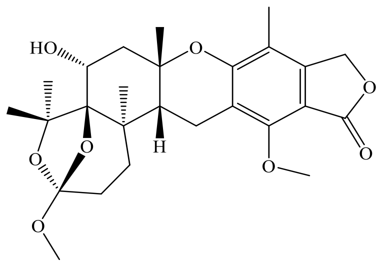

(38)

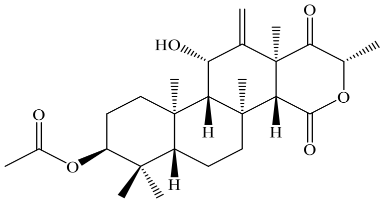

(41)

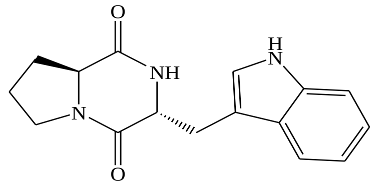

(42)

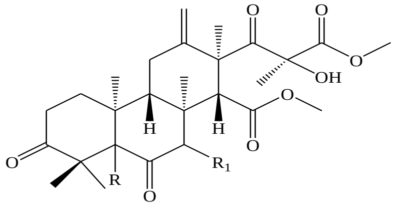

(43) R = *β*H; R_1 = *α*OH
(45) R = *α*H; R_1 = *α*OH

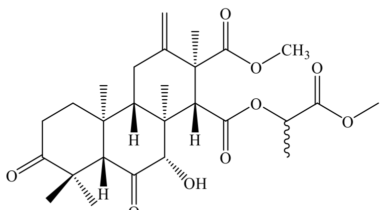

(44)

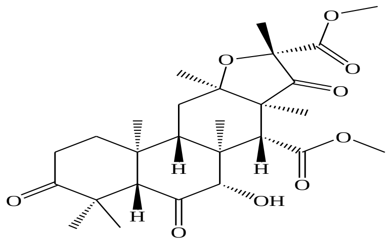

(46)

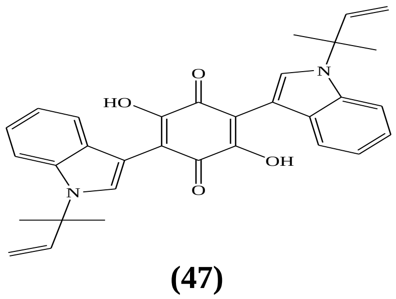

(47)

Figure S3 continue: Structure for tentatively identified compounds in the ethyl acetate extract of *A. terreus* by HPLC/ESI/MS analysis.

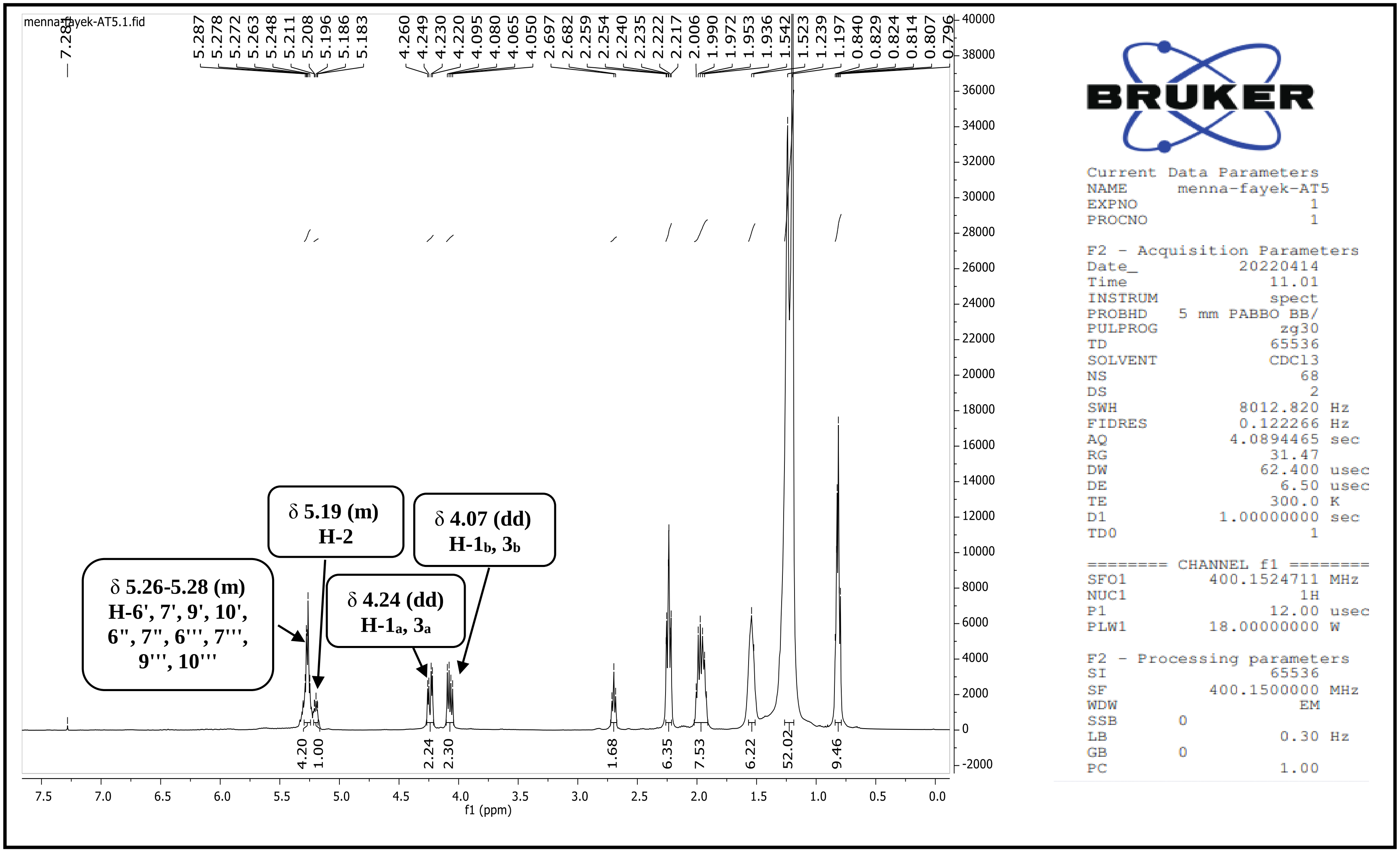

Figure S4. ^{1}HNMR spectrum of 1,3-di-(6*Z*,9*Z*)- trideca-6,9-dienoyl-2-(6Z) dodec-6-enoylglycerol ($CDCl_3$, 400 MHz)

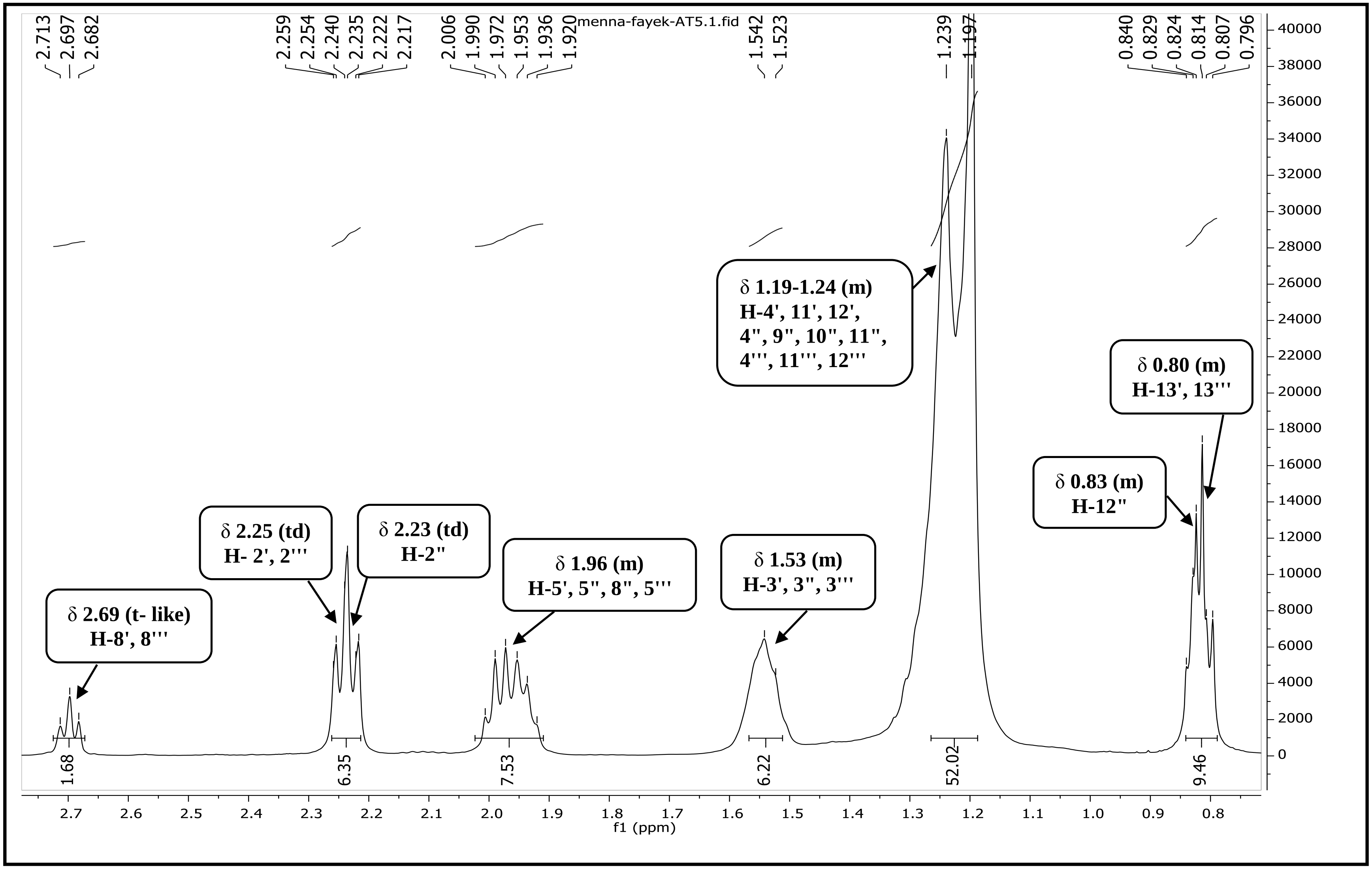

Figure S4 continue. **^{1}HNMR spectrum of 1,3-di-(6*Z*,9*Z*)- trideca-6,9-dienoyl-2-(6Z) dodec-6-enoylglycerol (CDCl₃, 400**

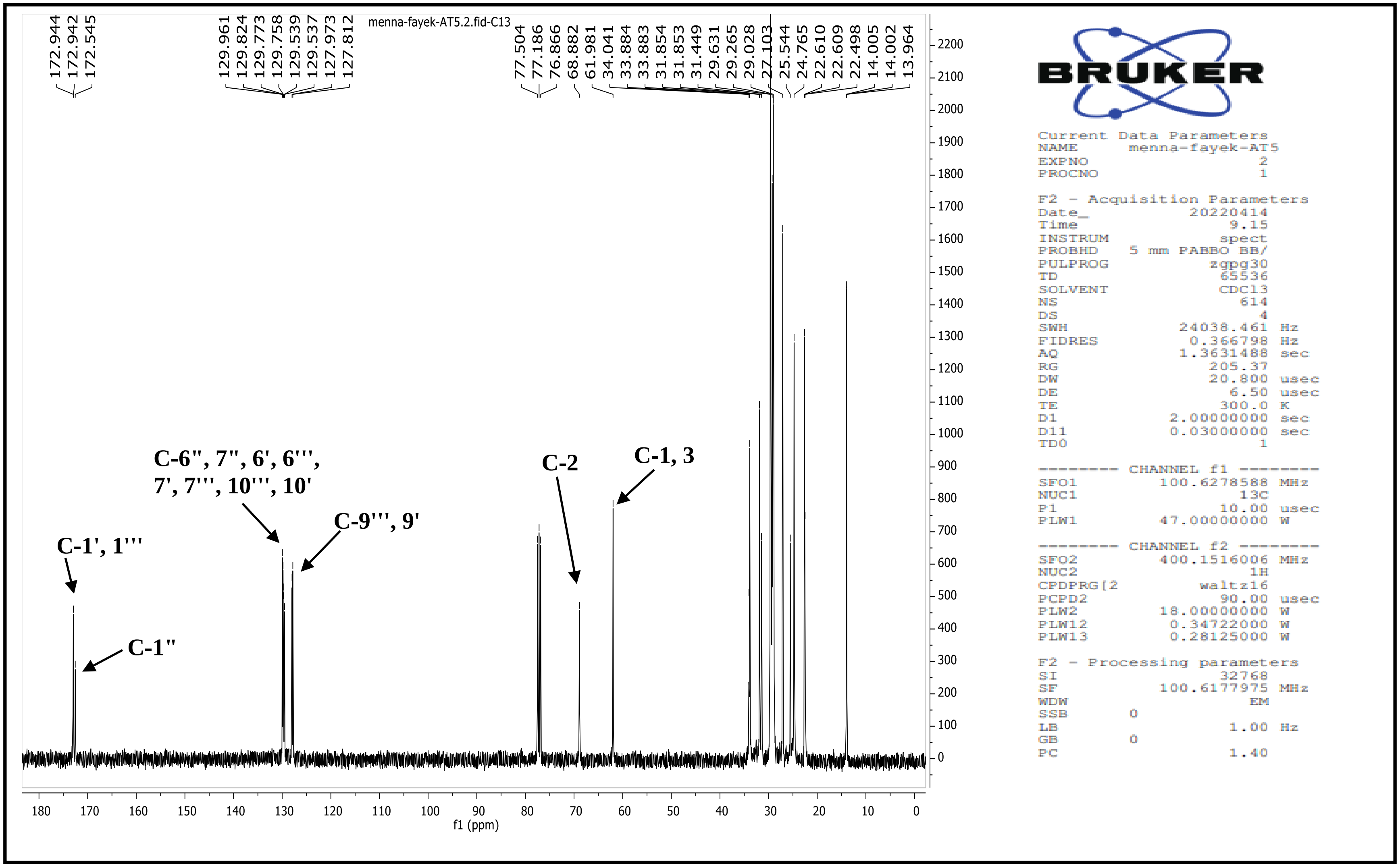

Figure S5. ^{13}**CNMR spectrum of 1,3-di-(6Z,9Z)- trideca-6,9-dienoyl-2-(6Z) dodec-6-enoylglycerol (CDCl$_3$, 100 MHz)**

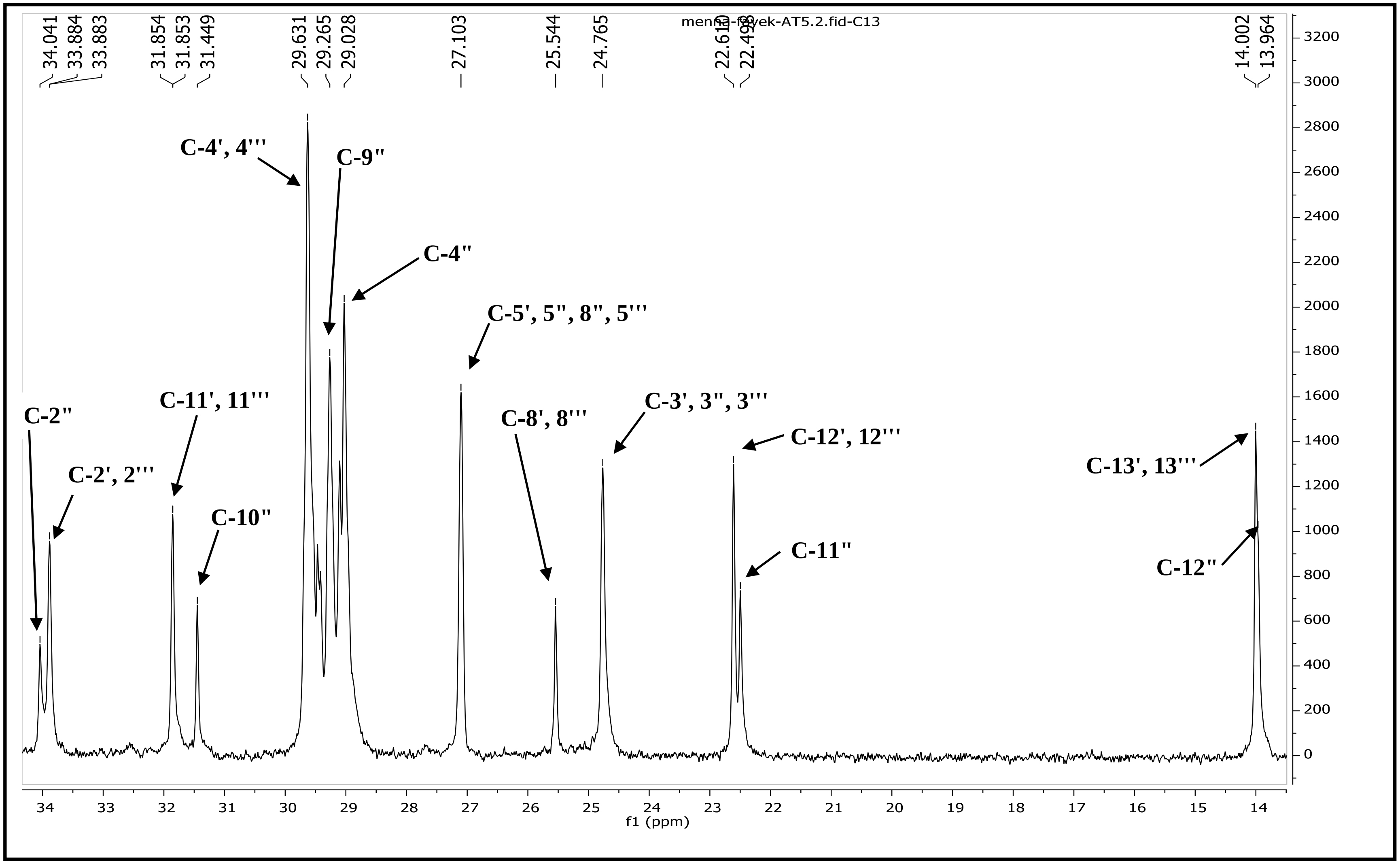

Figure S5 continue. **^{13}CNMR spectrum of 1,3-di-(6Z,9Z)- trideca-6,9-dienoyl-2-(6Z) dodec-6-enoylglycerol ($CDCl_3$, 100 MHz)**

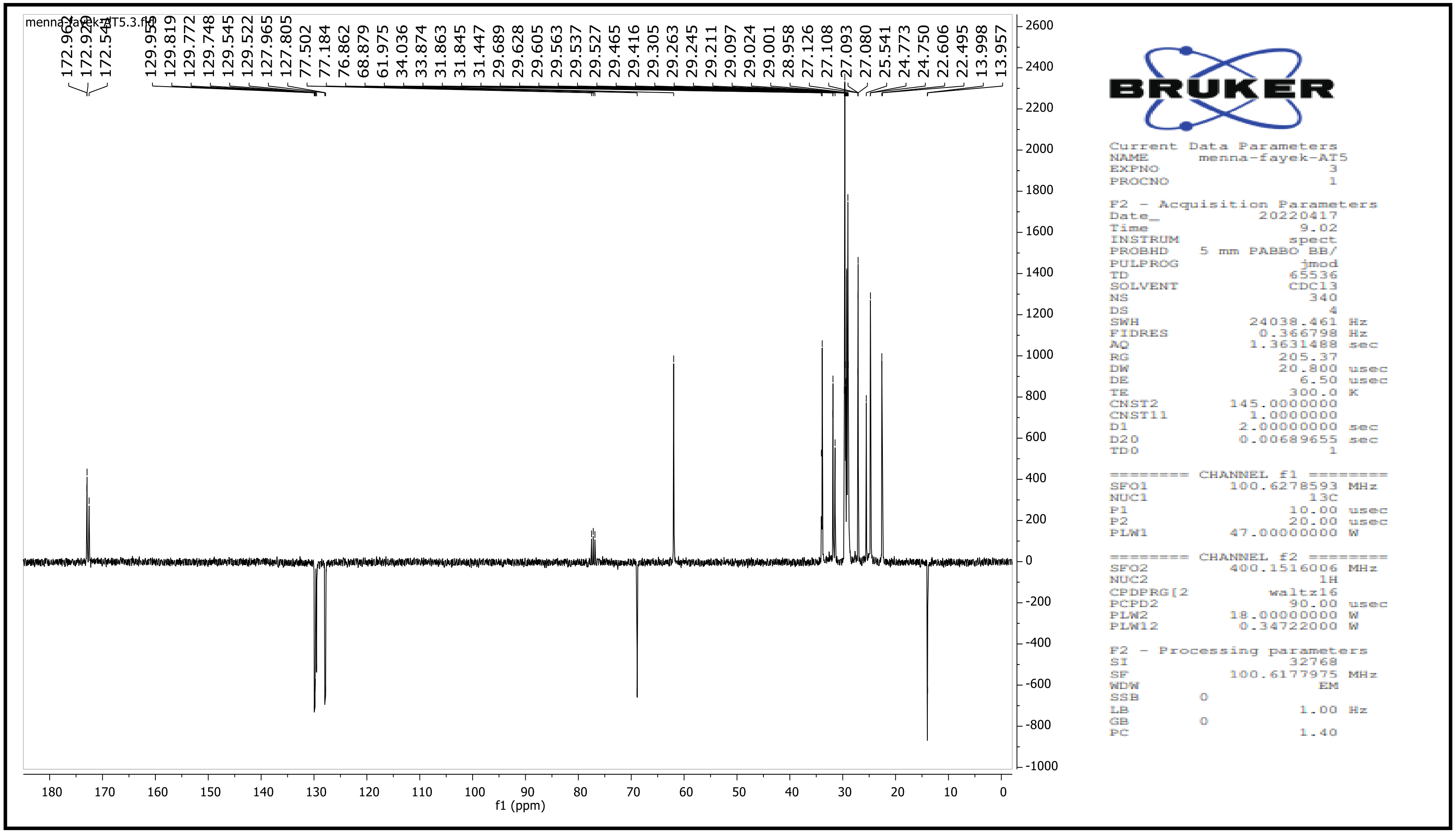

Figure S6. APT spectrum of 1,3-di-(6Z,9Z)- trideca-6,9-dienoyl-2-(6Z) dodec-6-enoylglycerol ($CDCl_3$, 100 MHz)

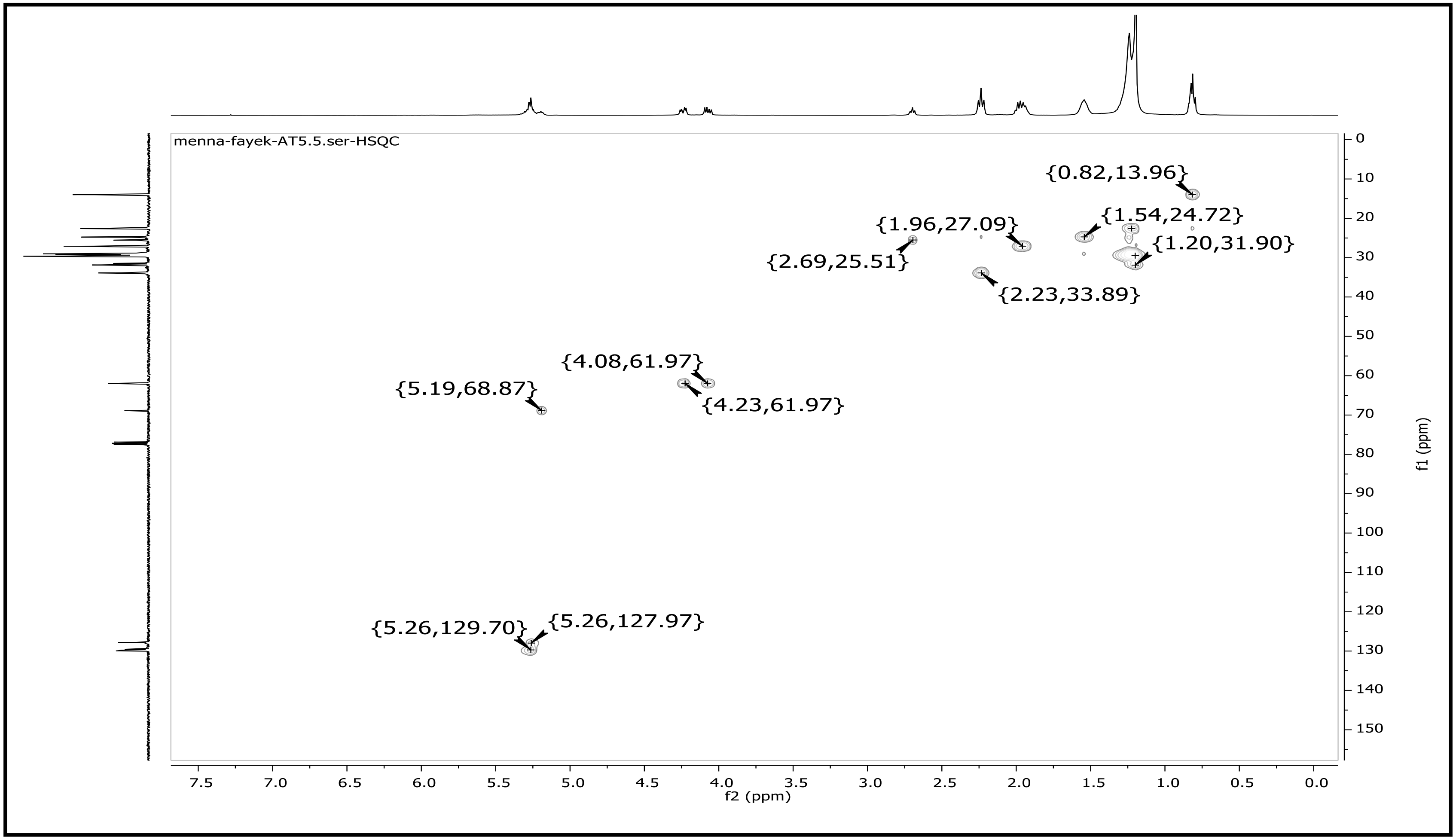

Figure S7. HSQC spectrum of 1,3-di-(6Z,9Z)- trideca-6,9-dienoyl-2-(6Z) dodec-6-enoylglycerol ($CDCl_3$, 400 MHz)

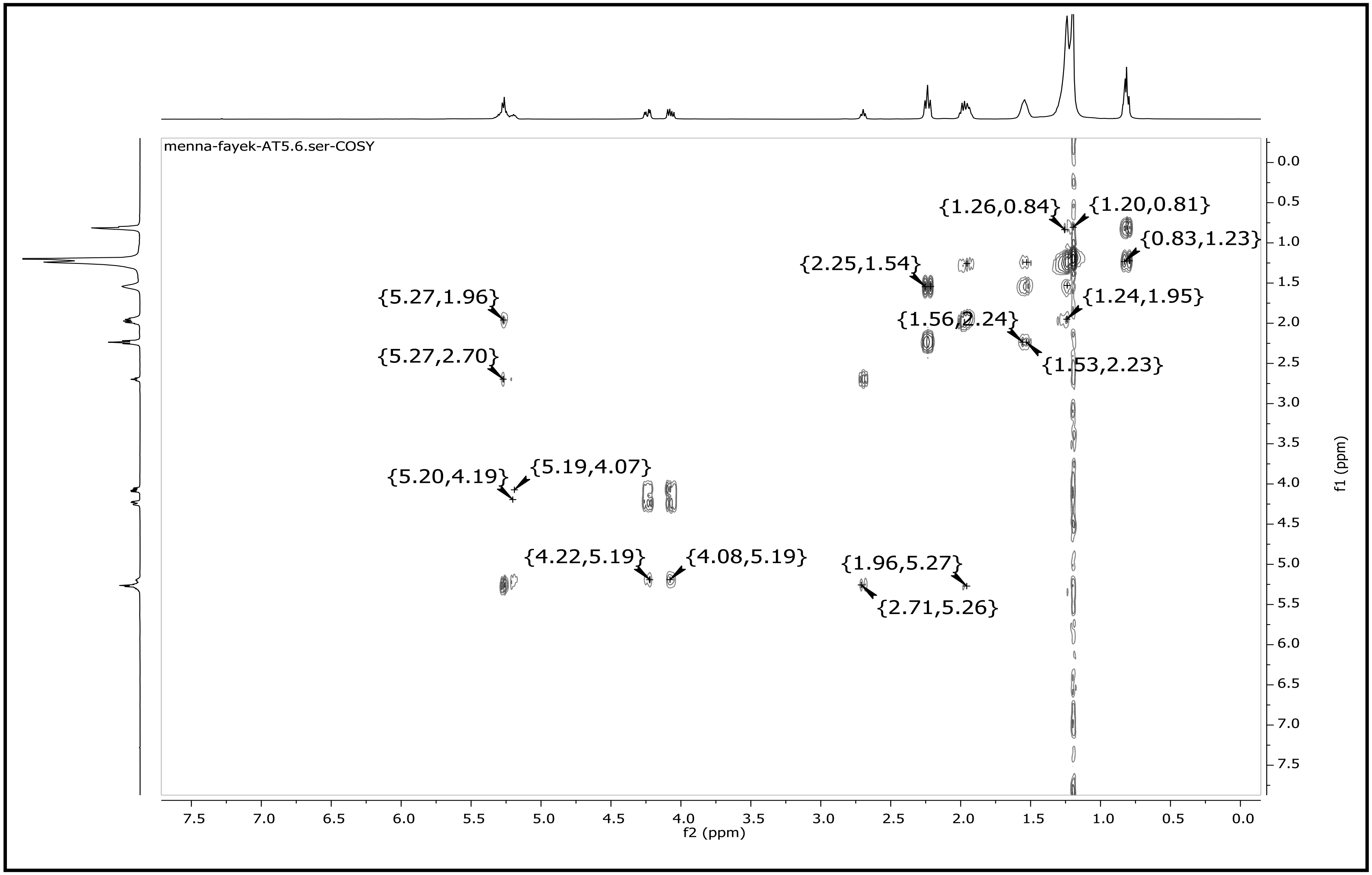

Figure S8. ^1H-^1H **COSY spectrum of 1,3-di-(6Z,9Z)- trideca-6,9-dienoyl-2-(6Z) dodec-6-enoylglycerol (** $CDCl_3$ **, 400 MHz)**

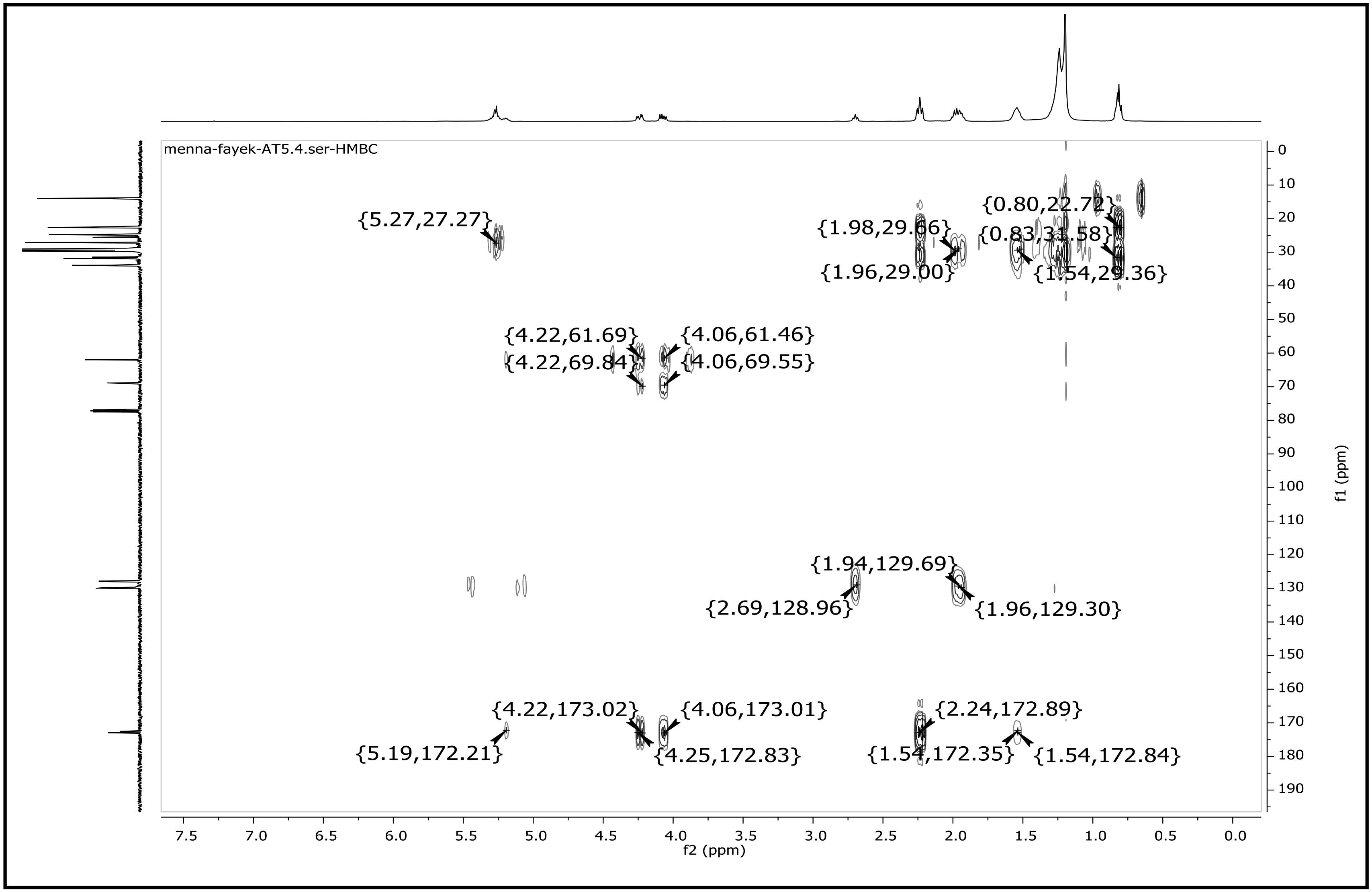

Figure S9. HMBC spectrum of 1,3-di-(6Z,9Z)- trideca-6,9-dienoyl-2-(6Z) dodec-6-enoylglycerol (CDCl$_3$, 400 MHz)

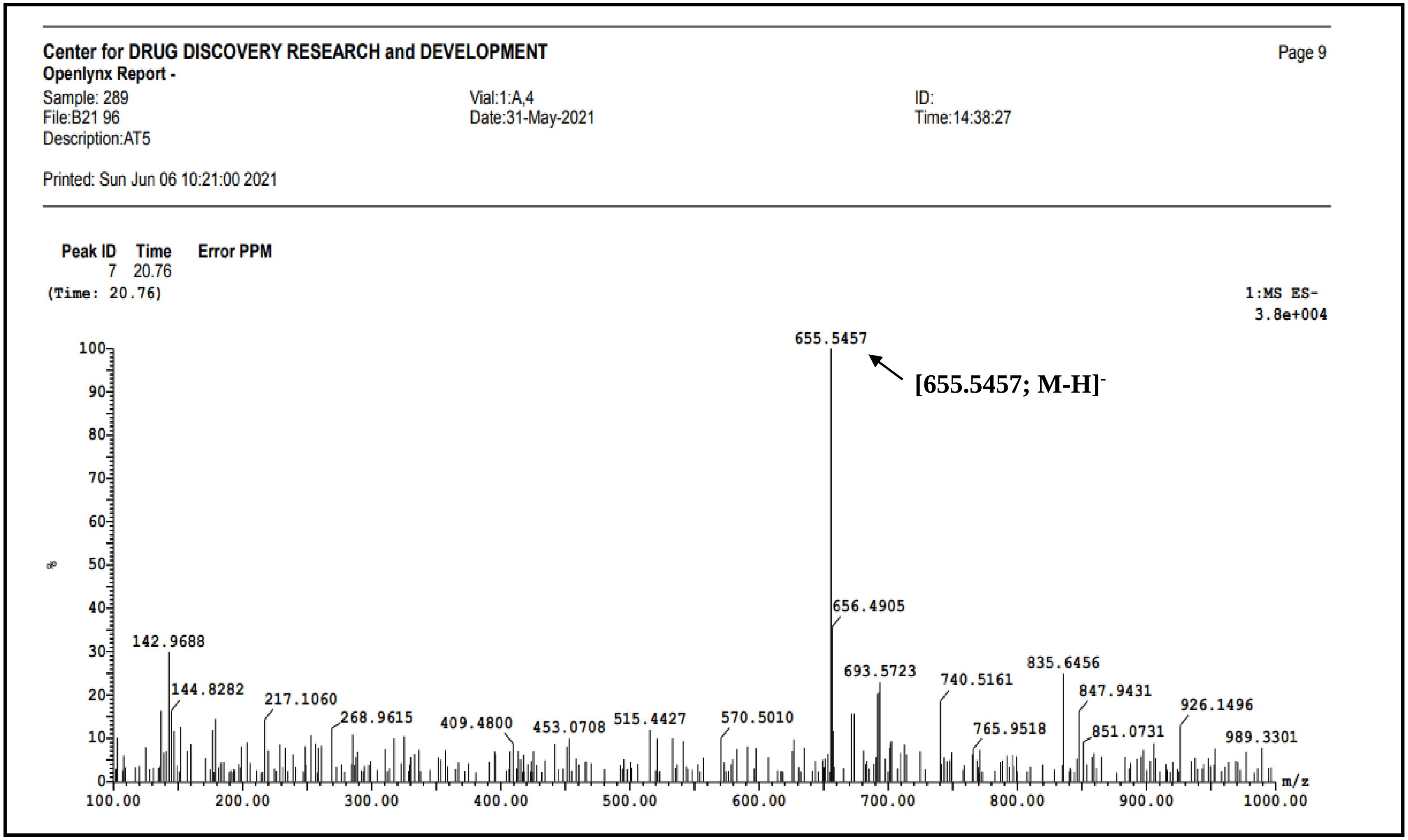

Figure S10. Negative ESI/MS spectrum of 1,3-di-(6Z,9Z)- trideca-6,9-dienoyl-2-(6Z) dodec-6-enoylglycero

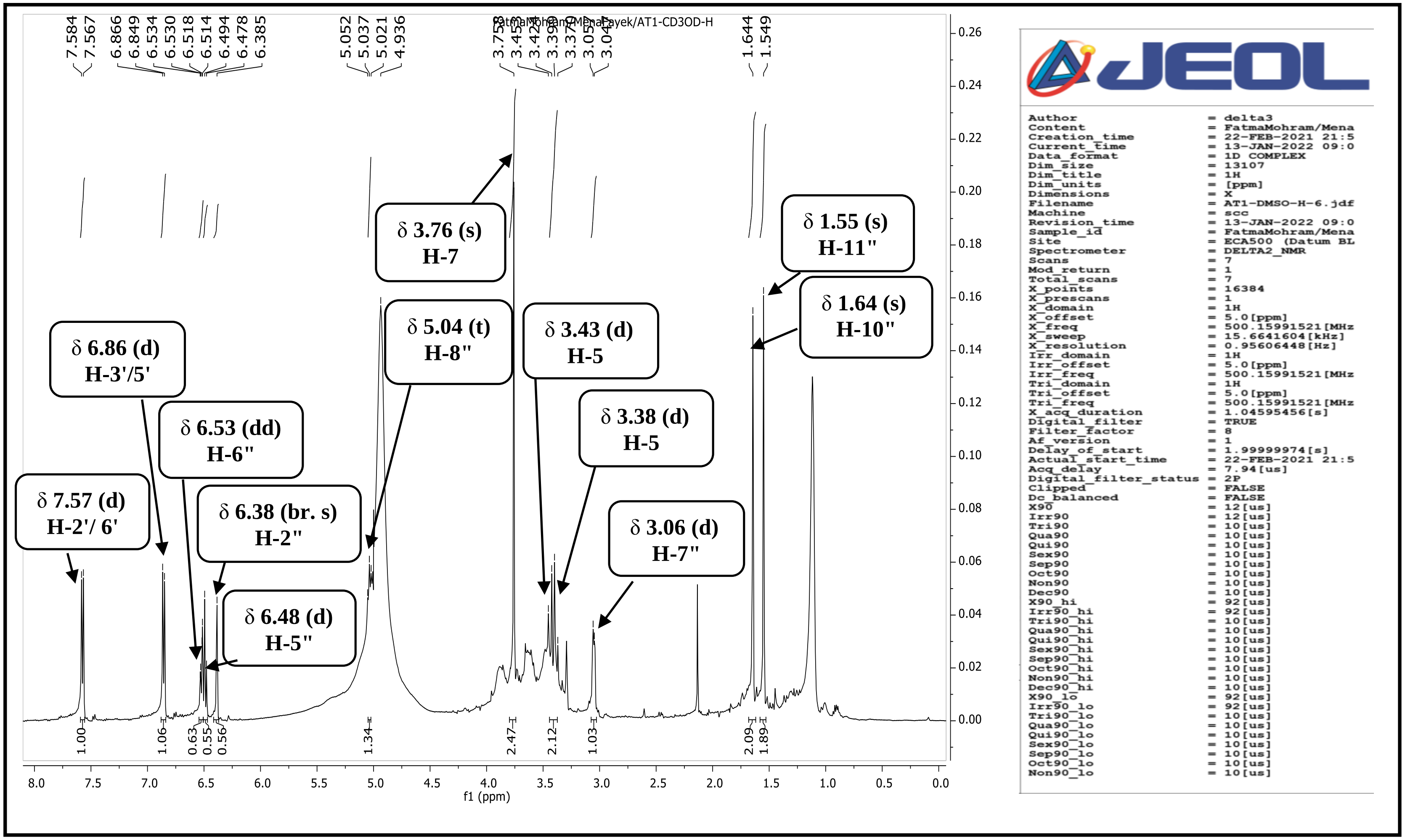

Figure S11. ^{1}**HNMR spectrum of compound 2 (CD$_3$OD-*d*, 500 MHz)**

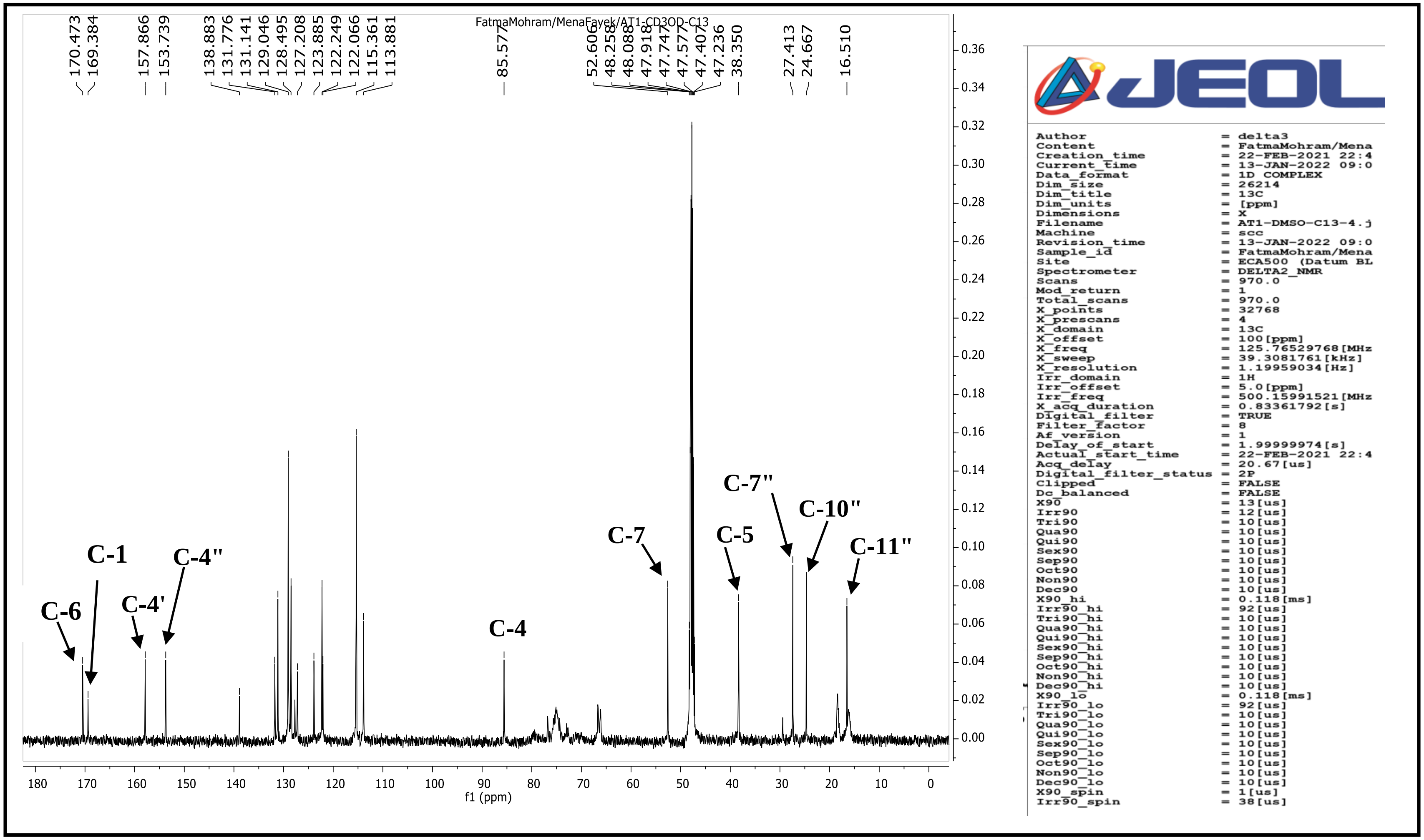

Figure S12. **^{13}CNMR spectrum of compound 2 (CD_3OD-*d*, 125 MHz)**

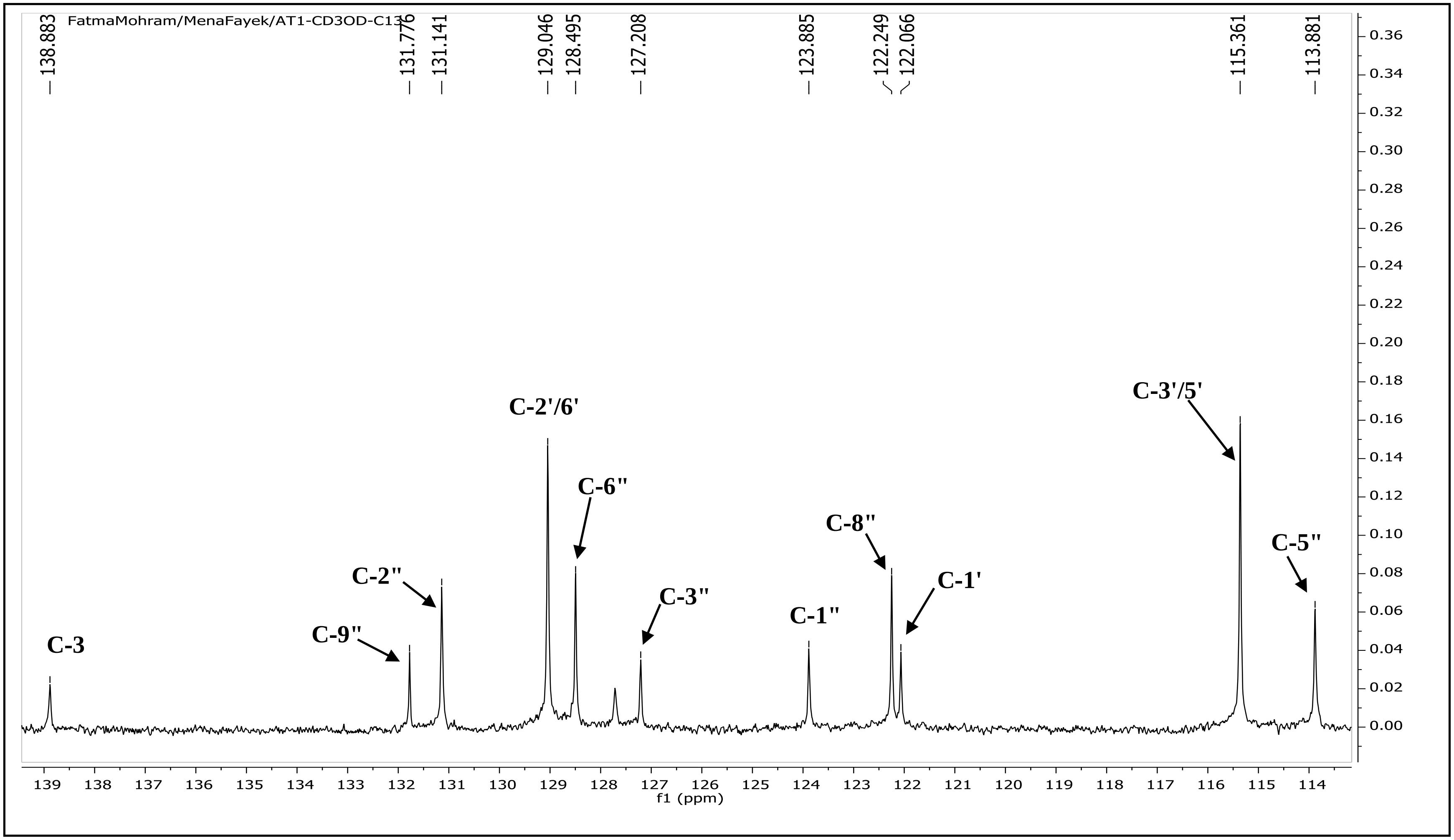

Figure S12 continue. ^{13}CNMR spectrum of compound 2 (CD_3OD-*d*, 125 MHz)

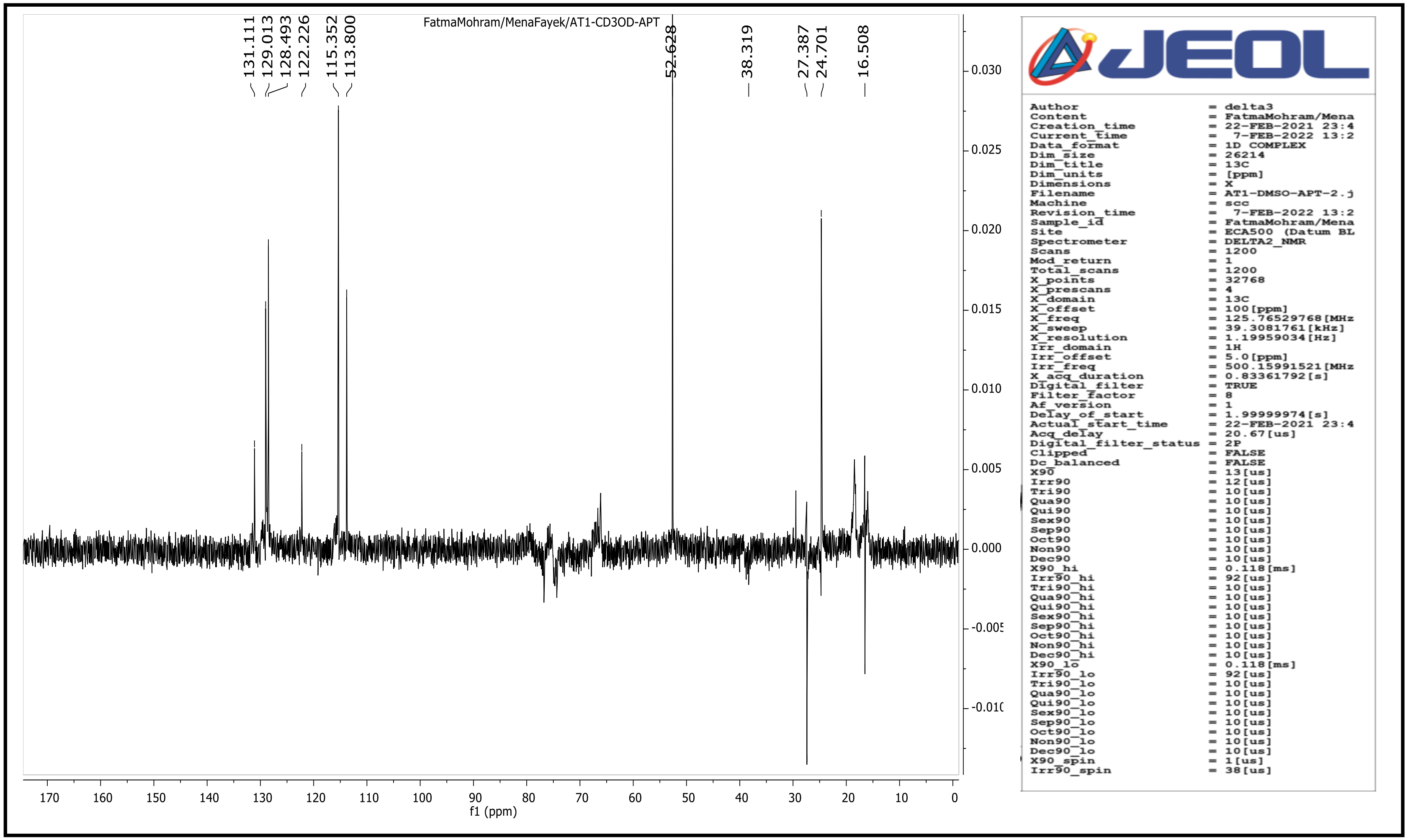

Figure S13. APT spectrum of compound 2 (CD_3OD-*d*, 125 MHz)

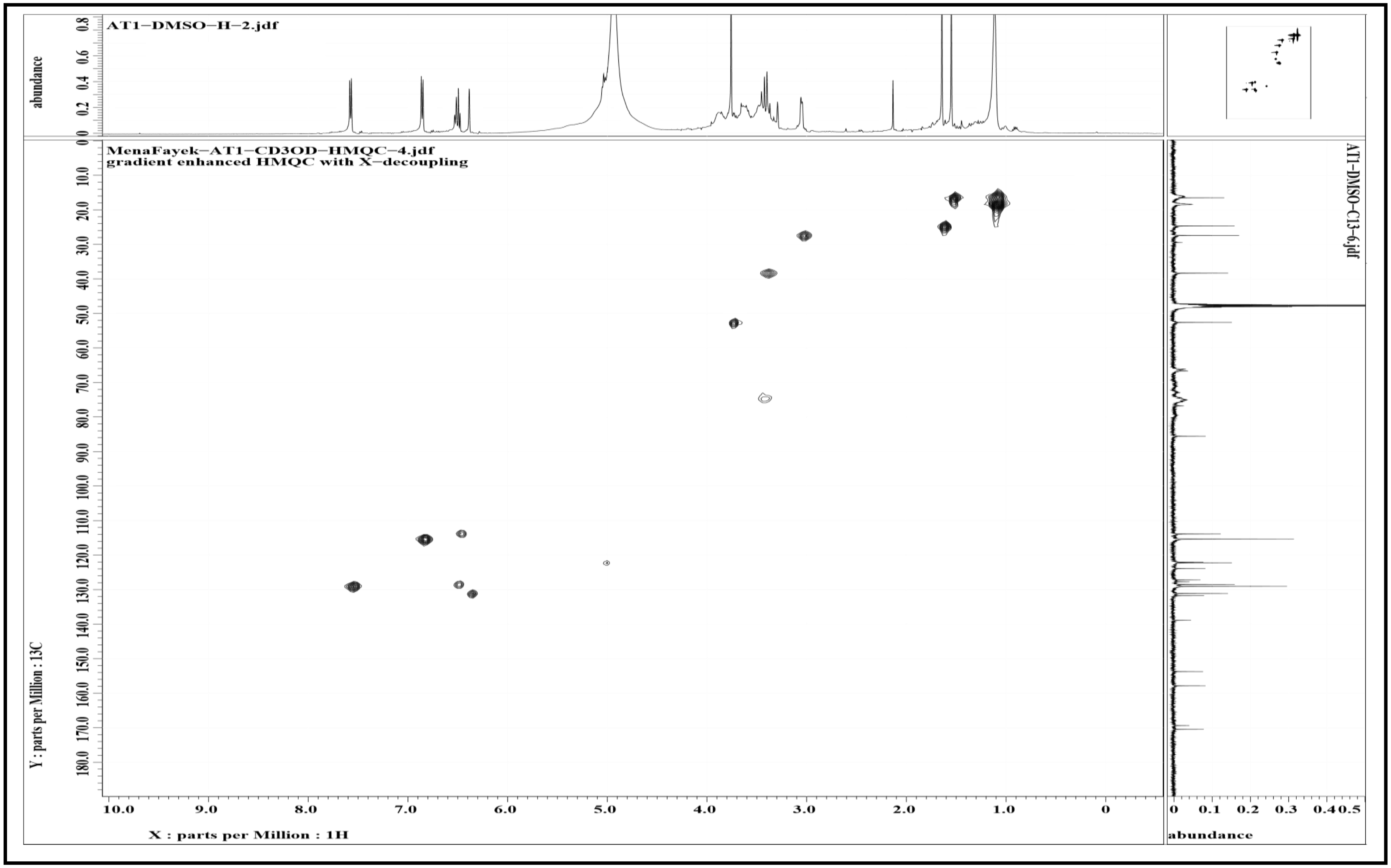

Figure S14. HSQC spectrum of compound 2 (CD$_3$OD-*d*, 500 MHz)

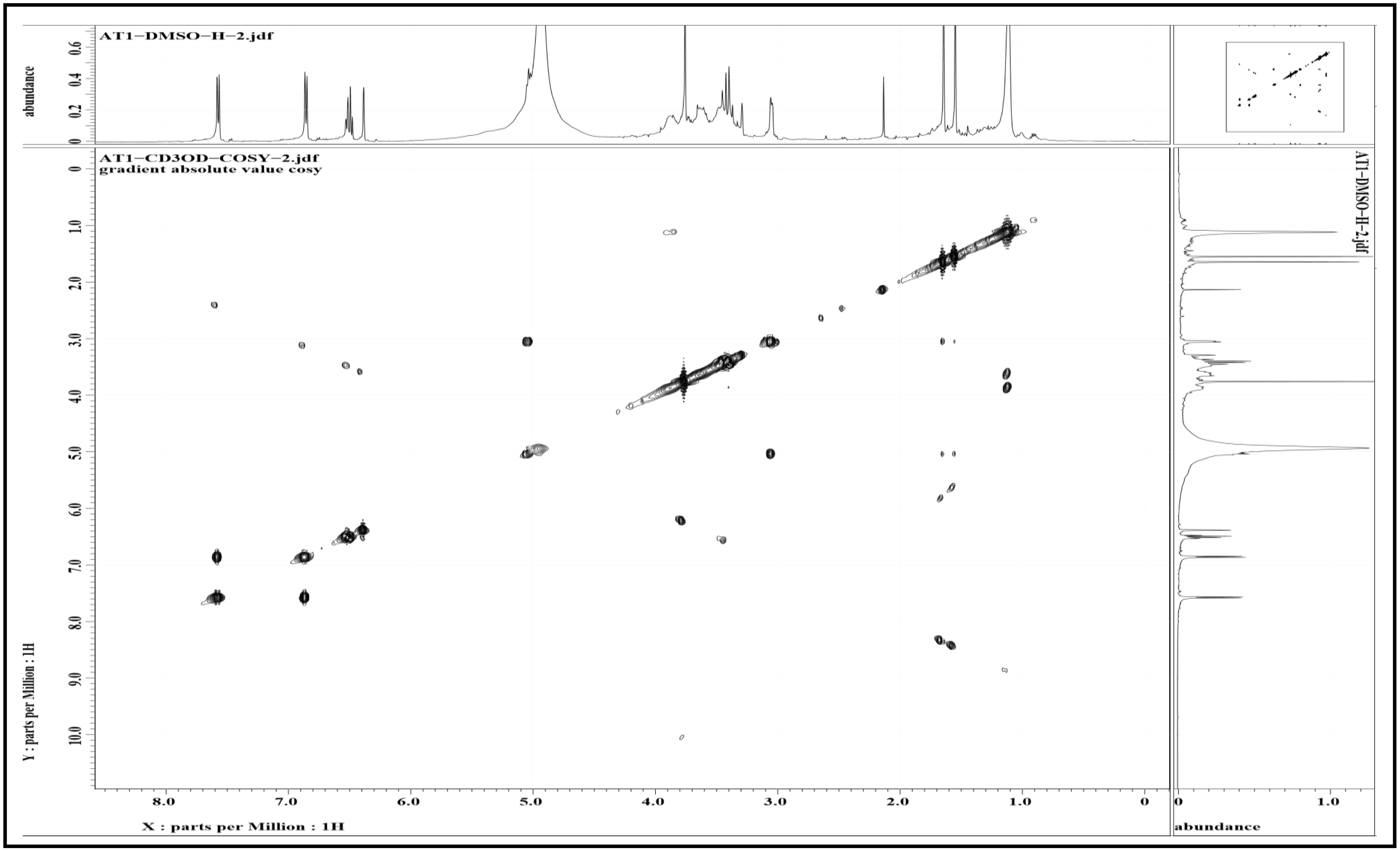

Figure S15. COSY spectrum of compound 2 (CD3OD-*d*, 500 MHz)

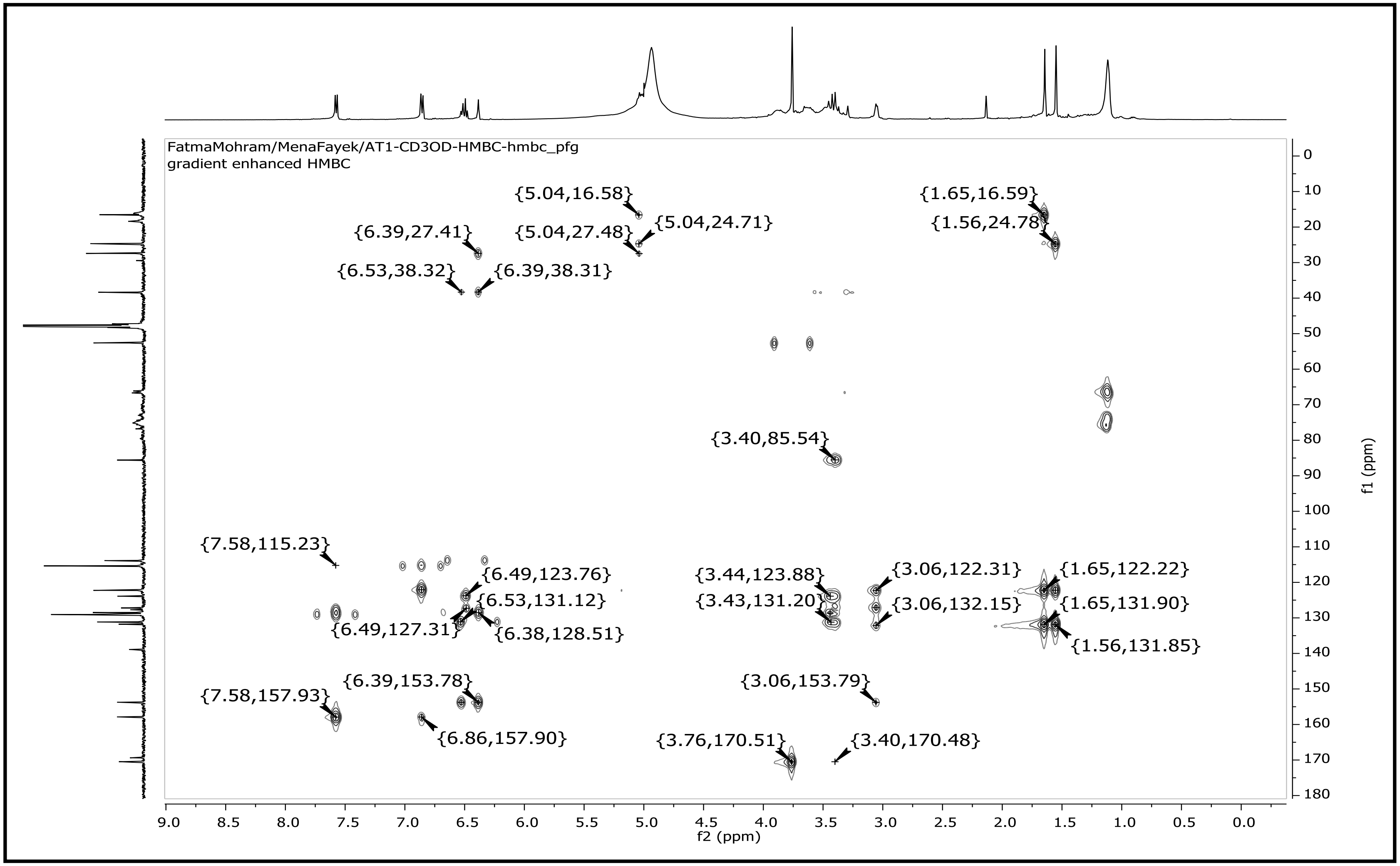

Figure S16. HMBC spectrum of compound 2 (CD_3OD-*d*, 500 MHz)

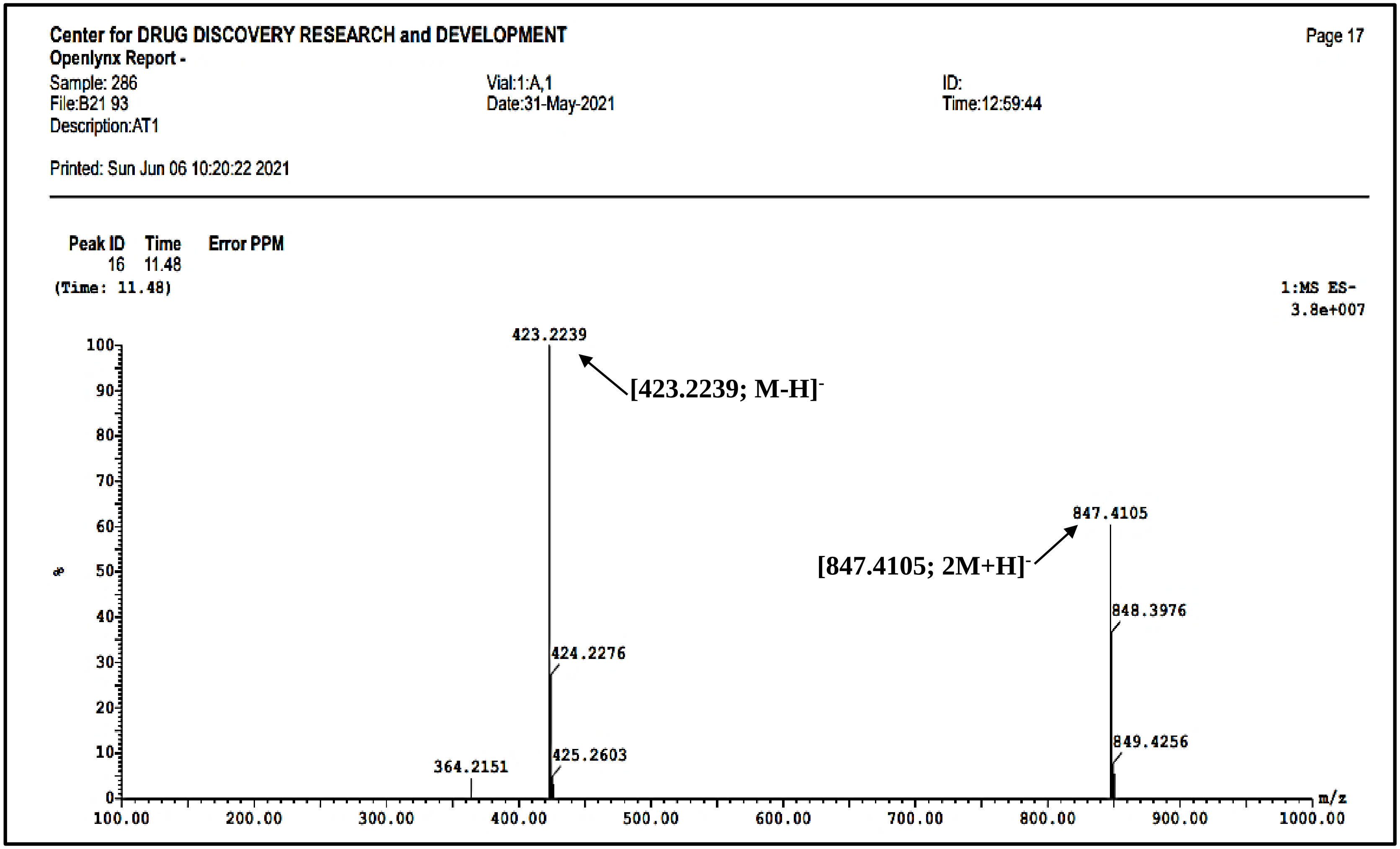

Figure S17. Negative ESI/MS spectrum of compound 2

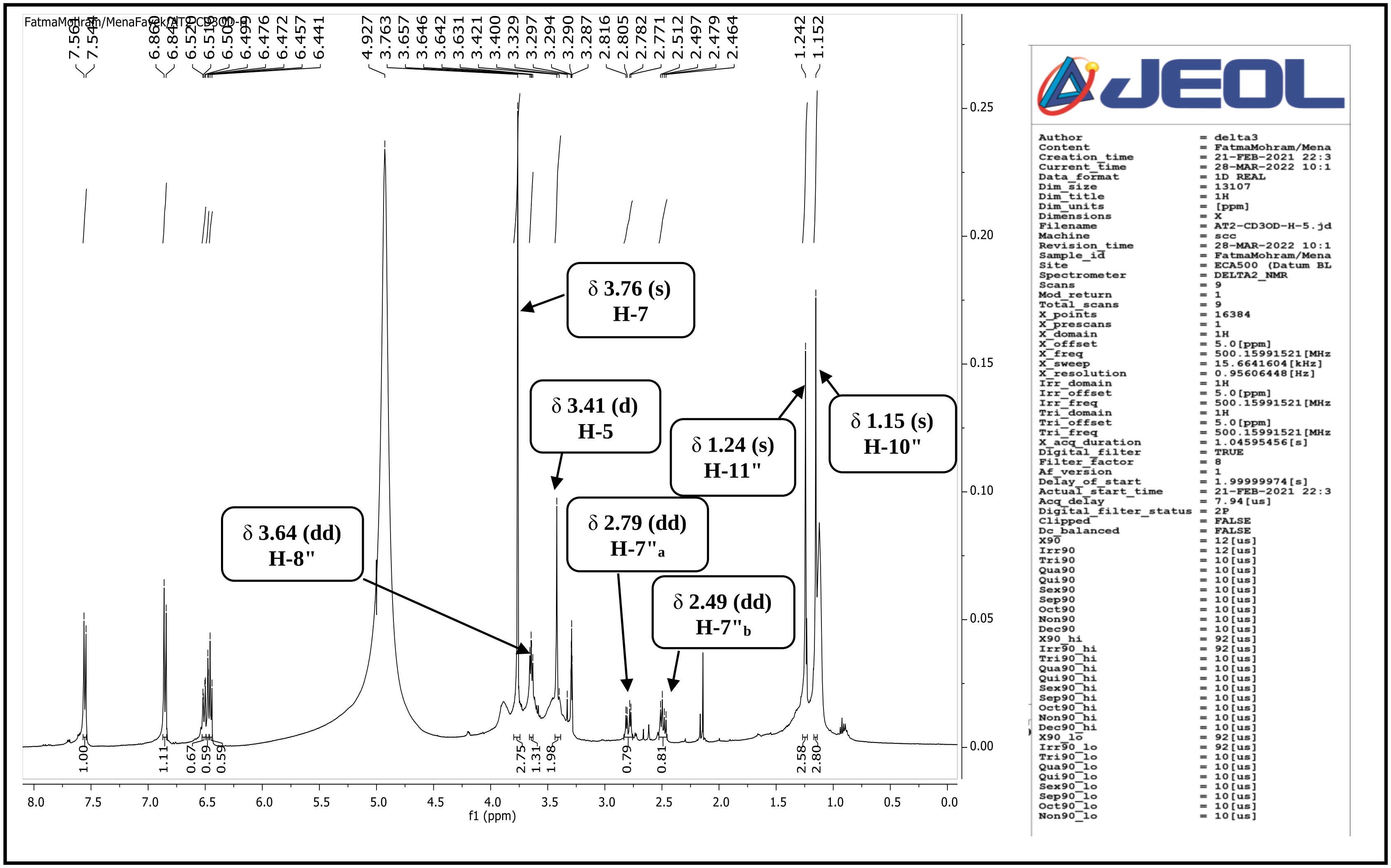

Figure S18. ^{1}**HNMR spectrum of compound 3 (CD$_3$OD-*d*, 500 MHz)**

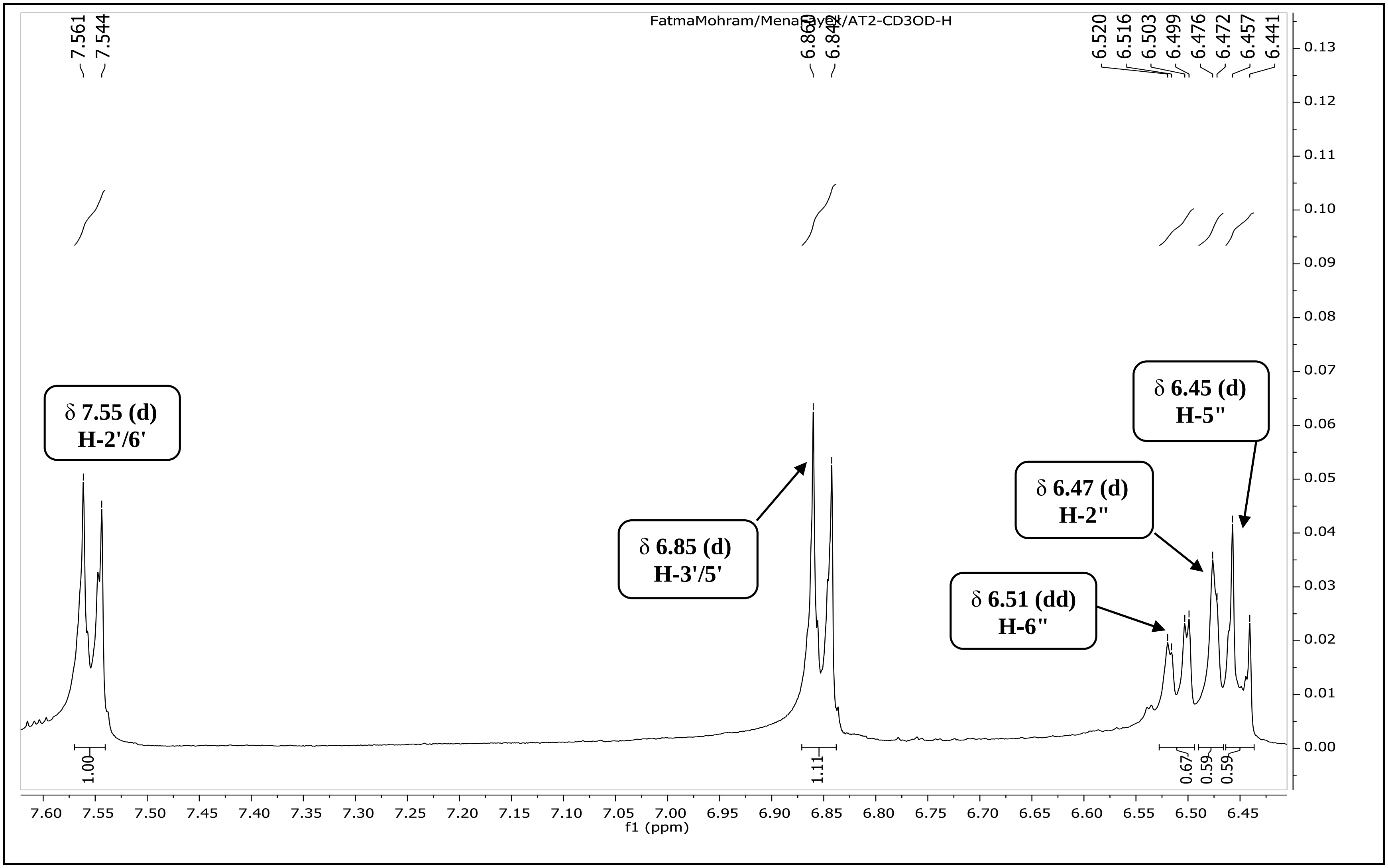

Figure S18 continue. ^{1}HNMR spectrum of compound 3 (CD_3OD-*d*, 500 MHz)

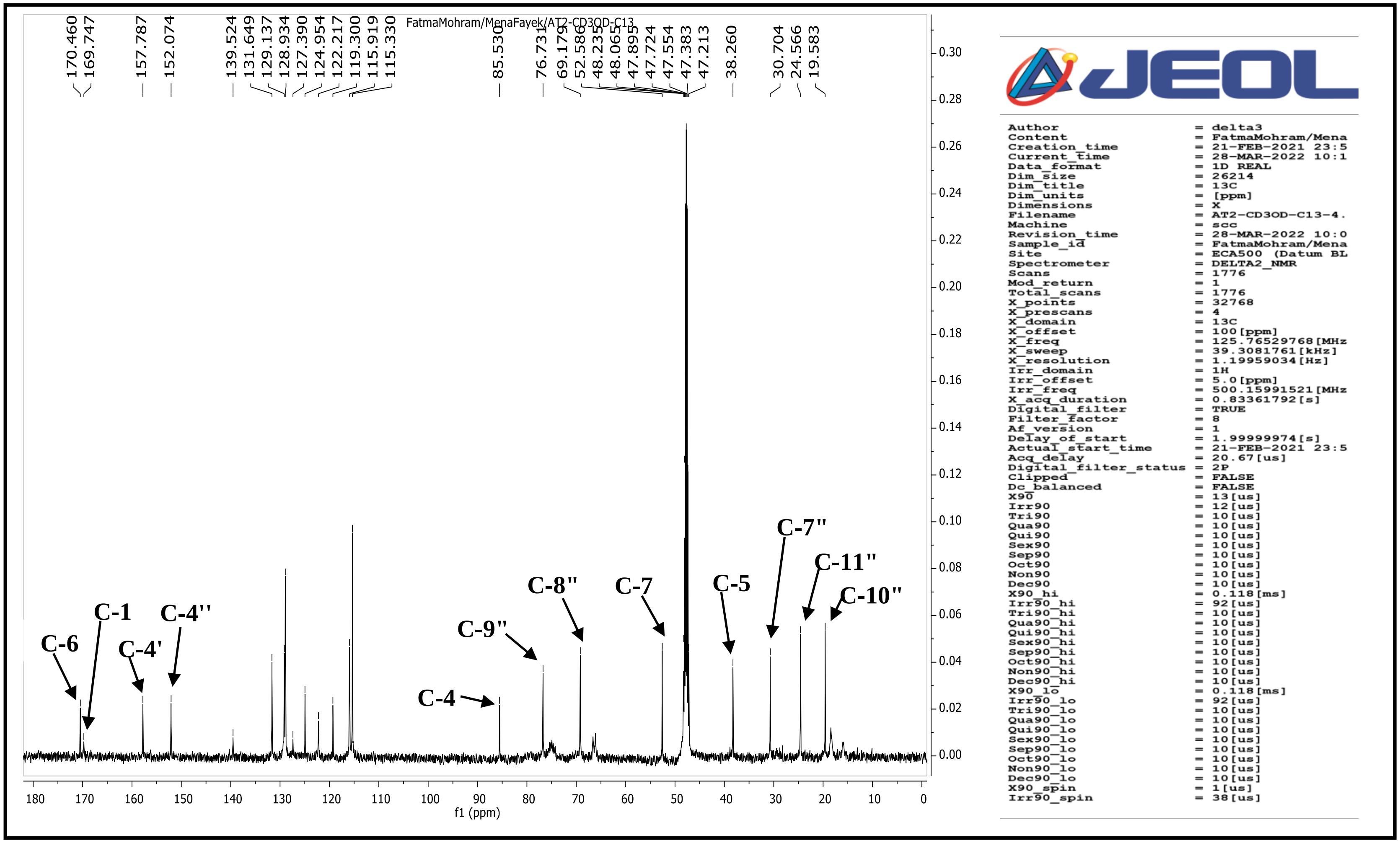

Figure S19. ^{13}**CNMR spectrum of compound 3 (**CD_3OD**-*d*, 125 MHz)**

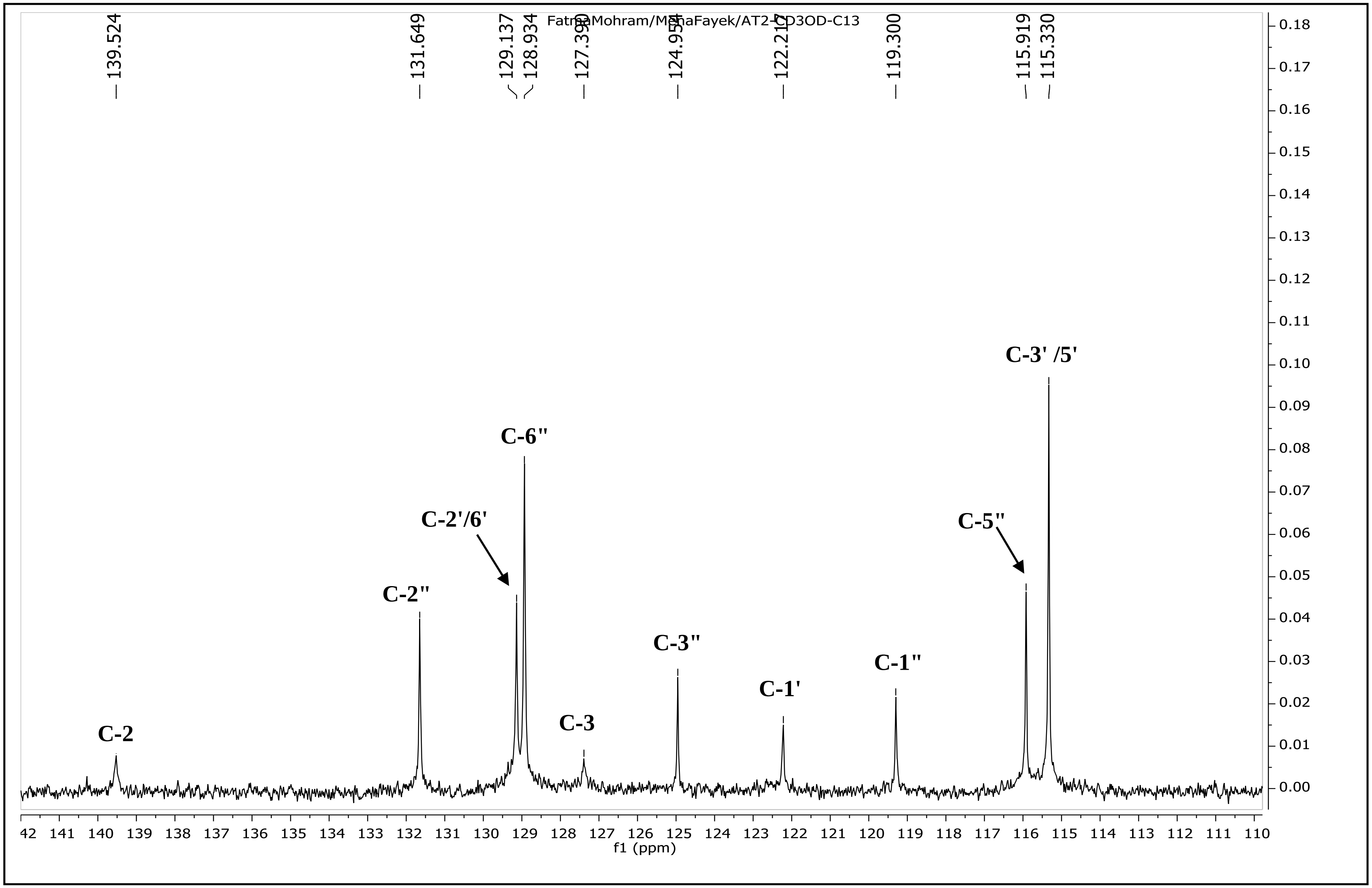

Figure S19 continue. ^{13}**CNMR spectrum of compound 3 (CD$_3$OD-*d*, 125 MHz)**

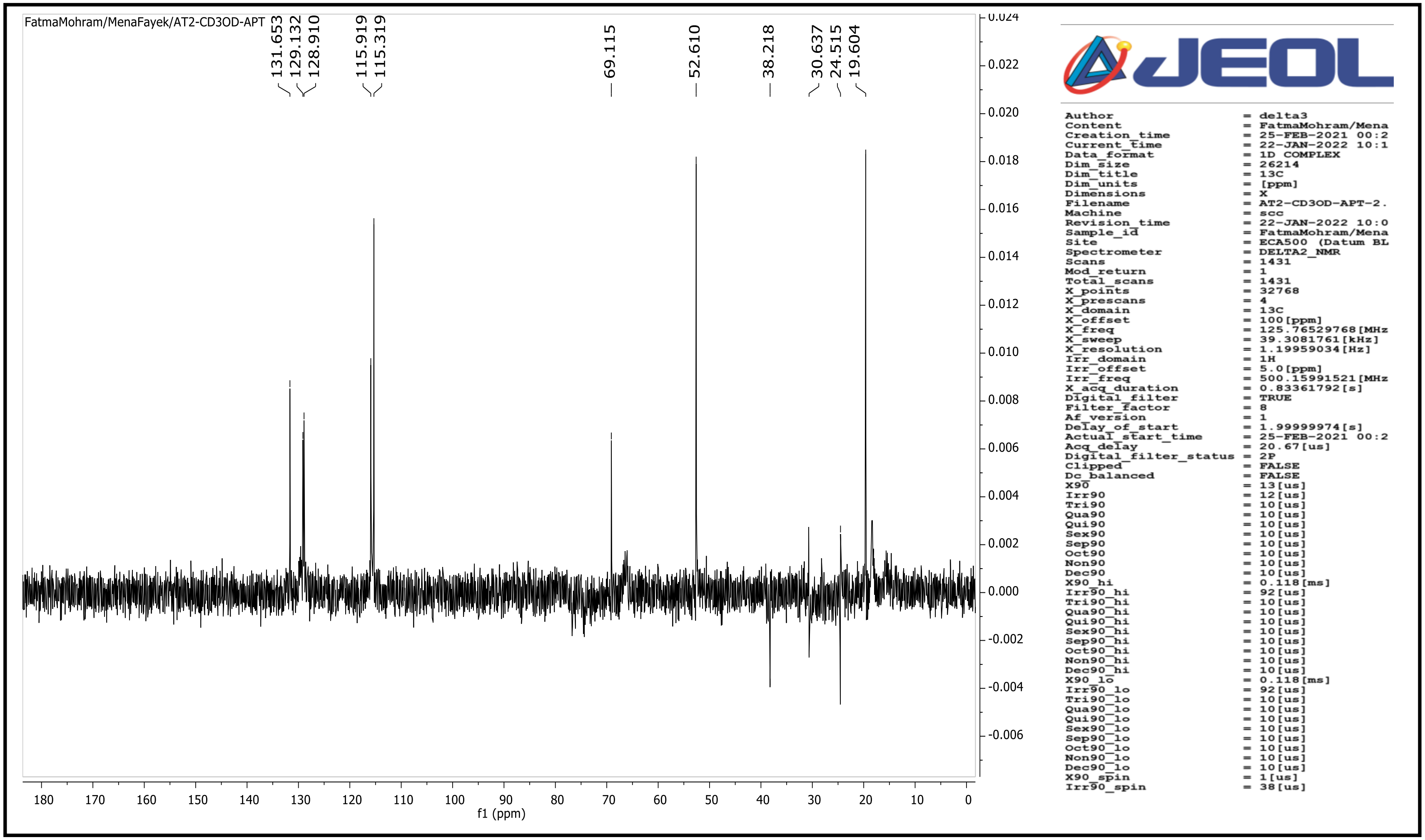

Figure S20. APT spectrum of compound 3 (CD_3OD-*d*, 125 MHz)

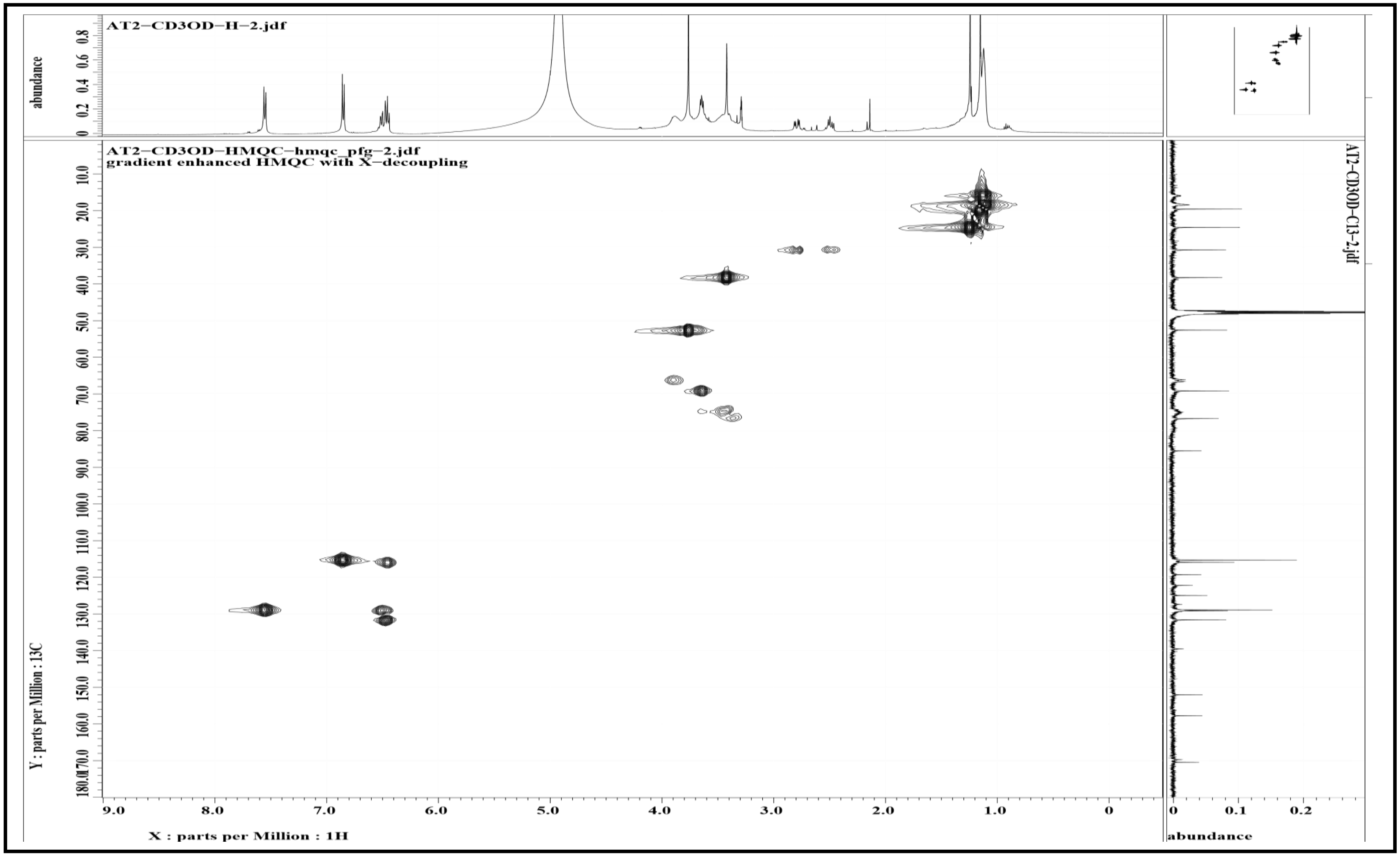

Figure S21. HSQC spectrum of compound 3 (CD_3OD-*d*, 500 MHz)

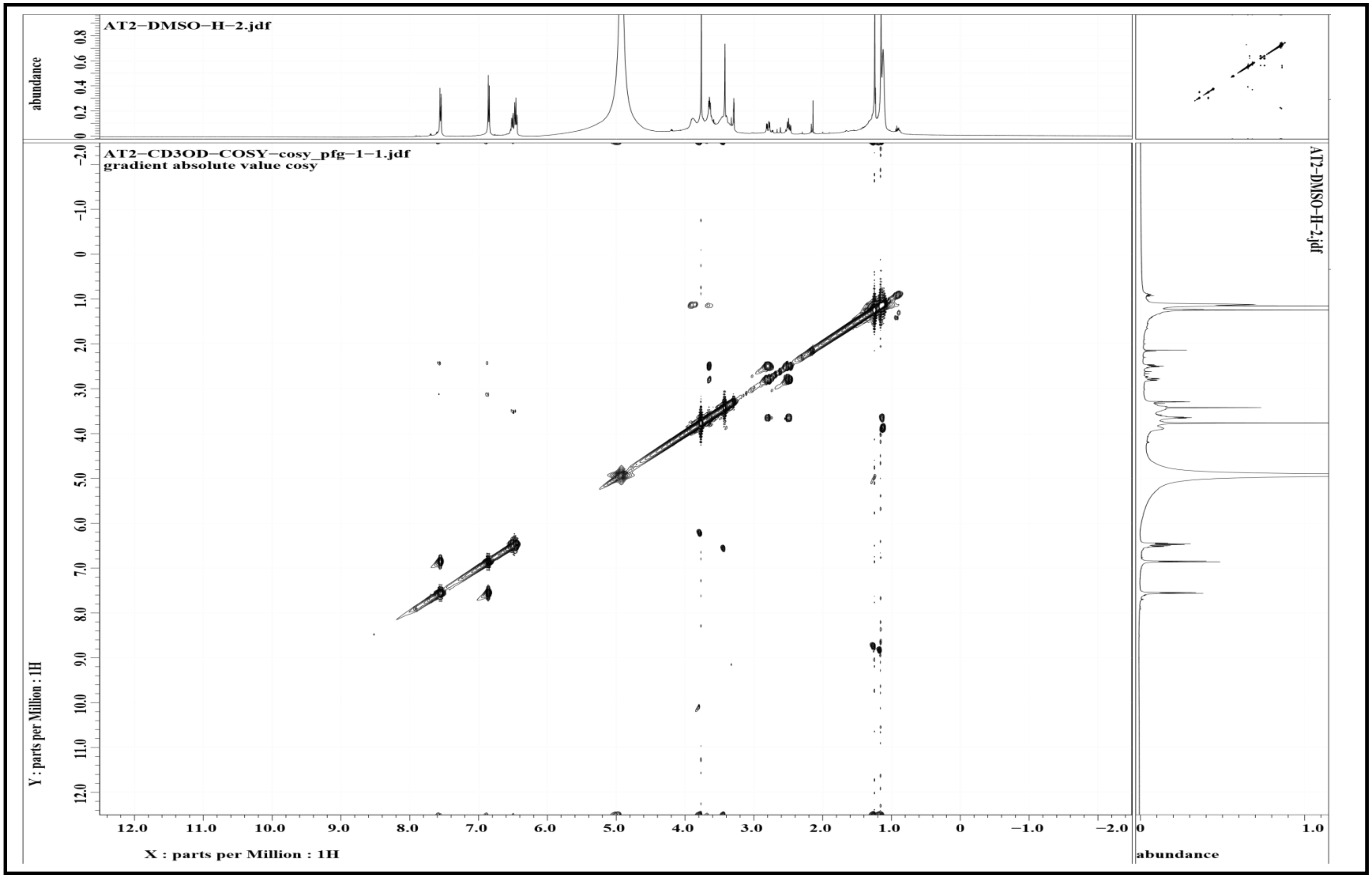

Figure S22. ^{1}H-^{1}H **COSY spectrum of compound 3 (CD_3OD-*d*, 500 MHz)**

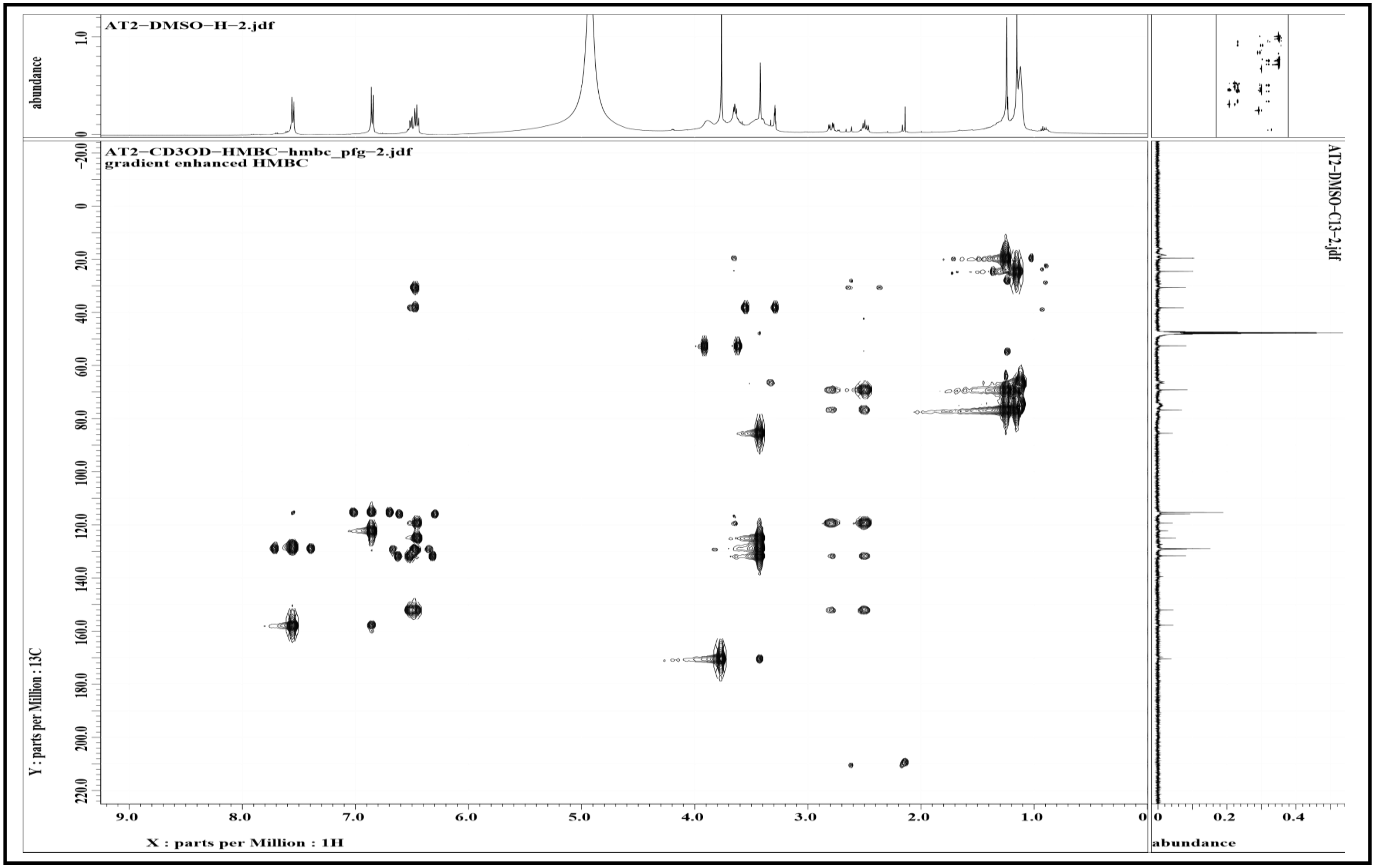

Figure S23. HMBC spectrum of compound 3 (CD_3OD-*d*, 500 MHz)

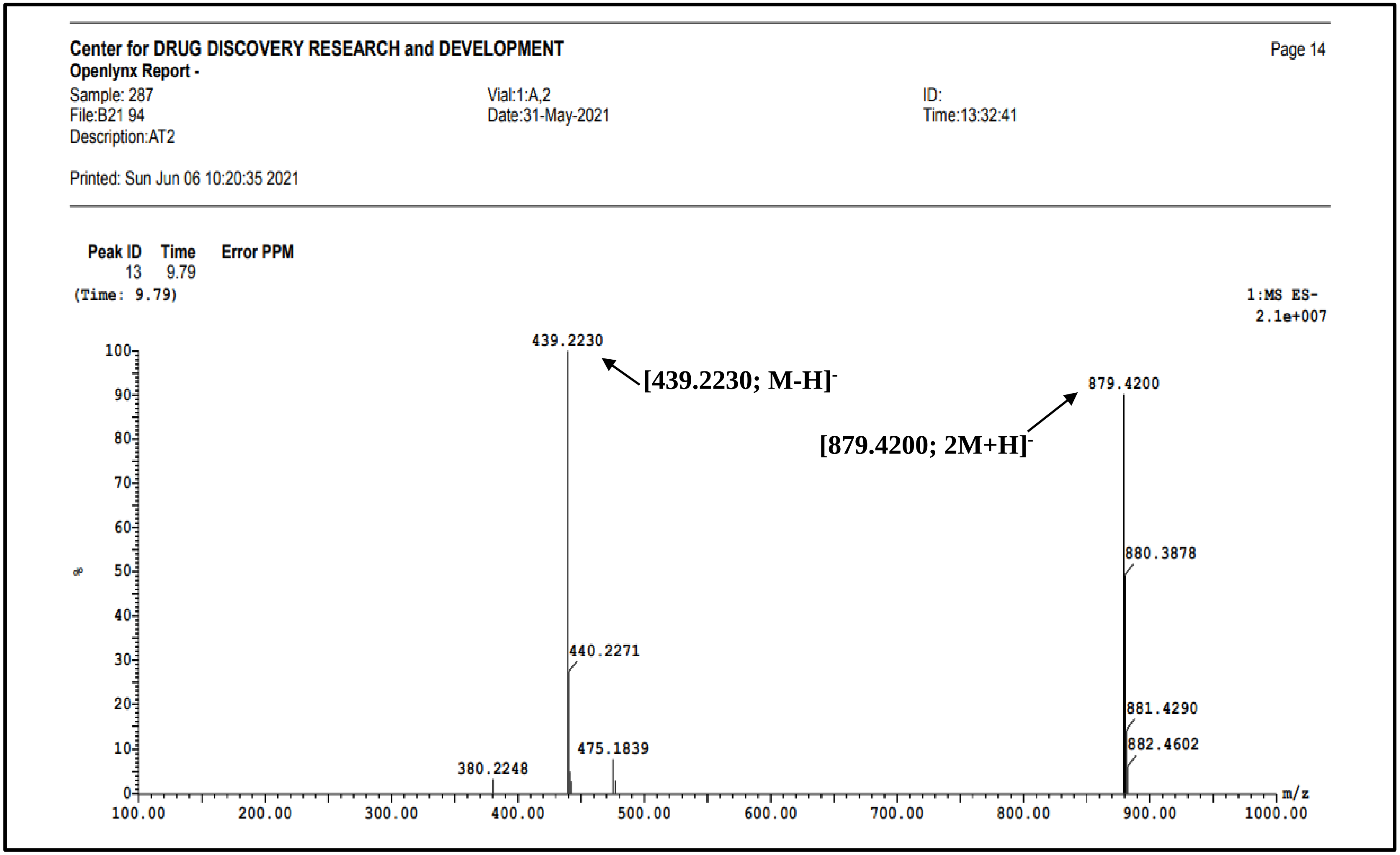

Figure S24. Negative ESI/MS spectrum of compound 3

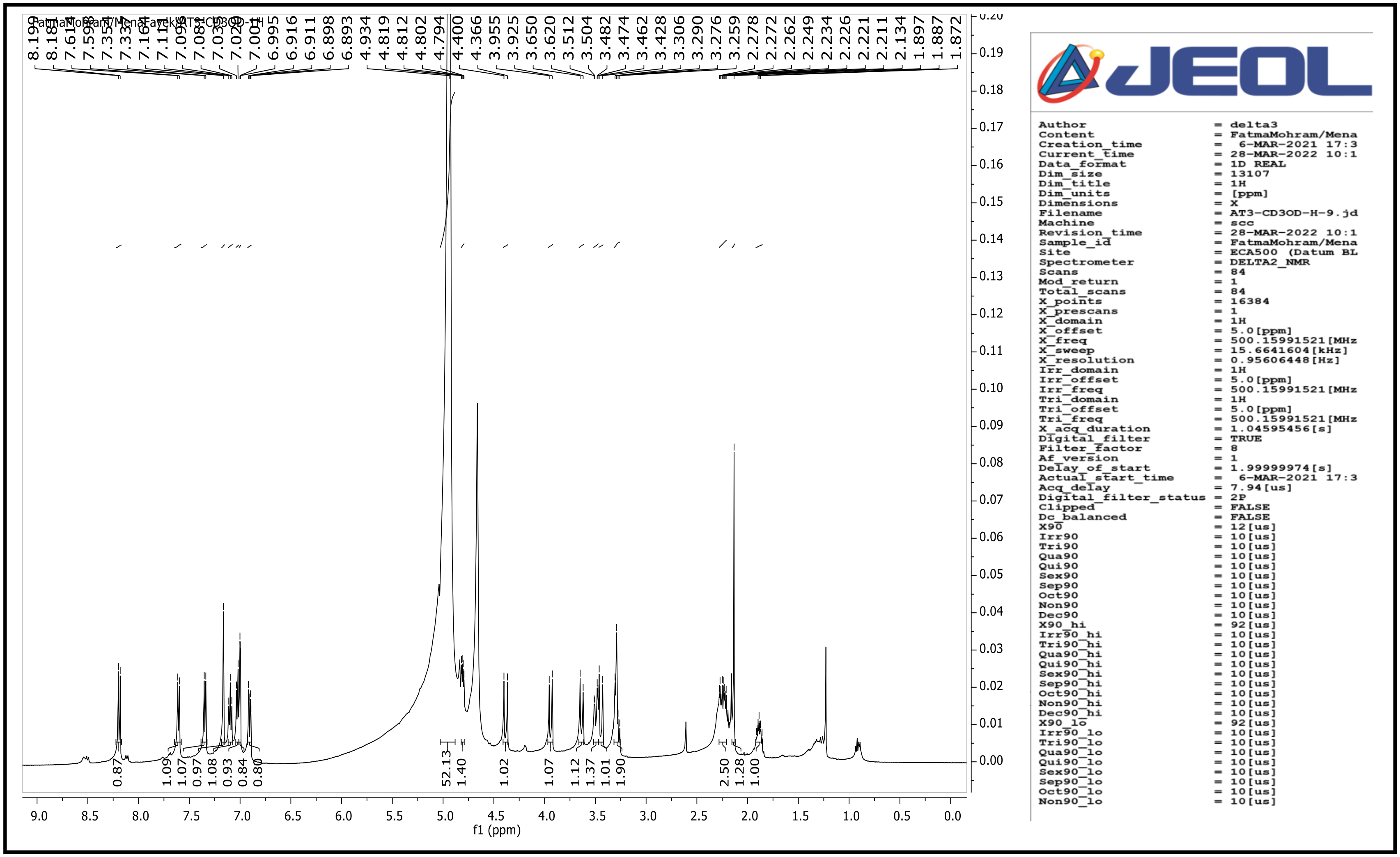

Figure S25. ^{1}**HNMR spectrum of compound 4 (CD$_3$OD-*d*, 500 MHz)**

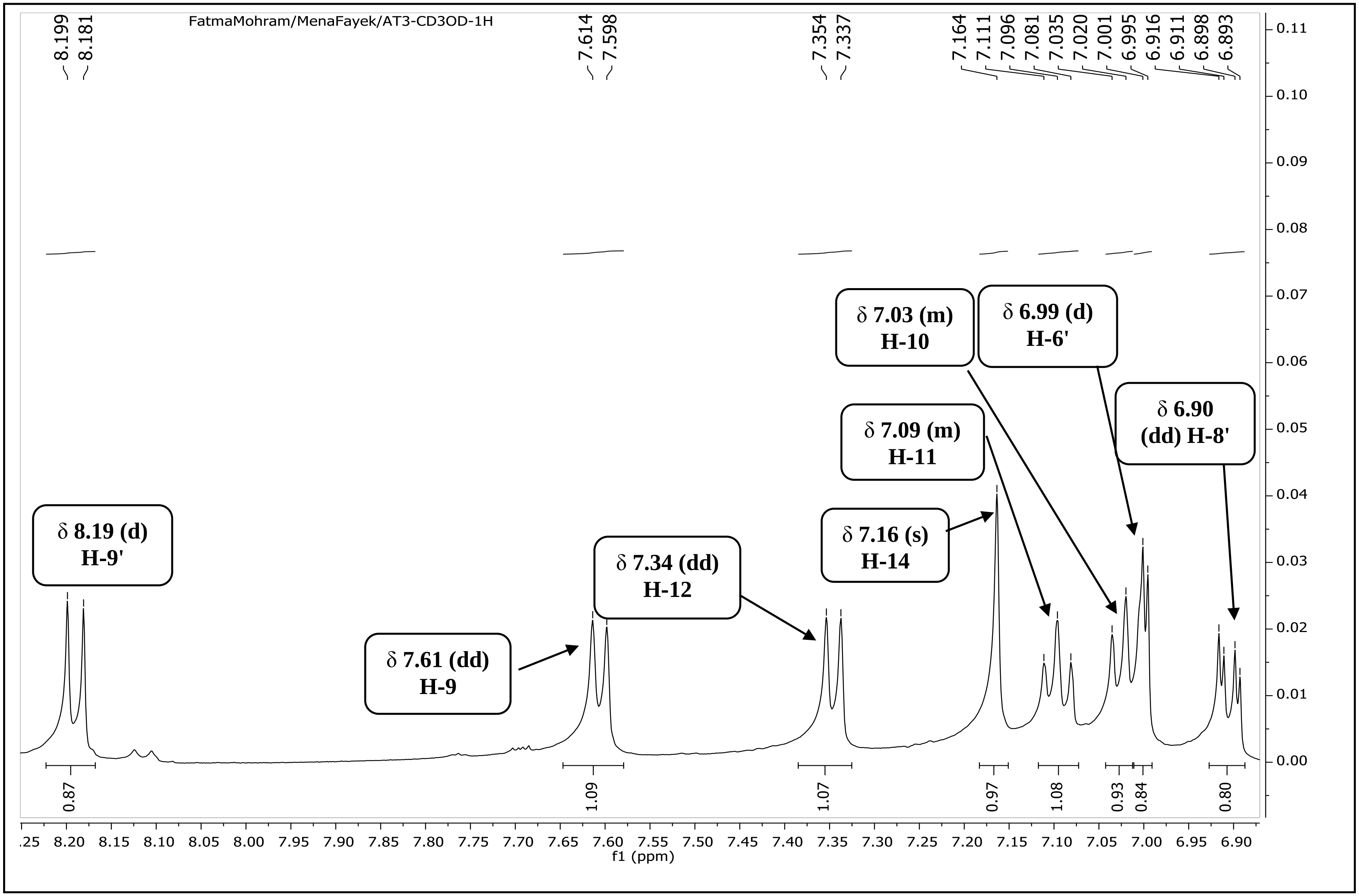

Figure S25 continue. ^{1}HNMR spectrum of compound 4 (CD_3OD-*d*, 500 MHz)

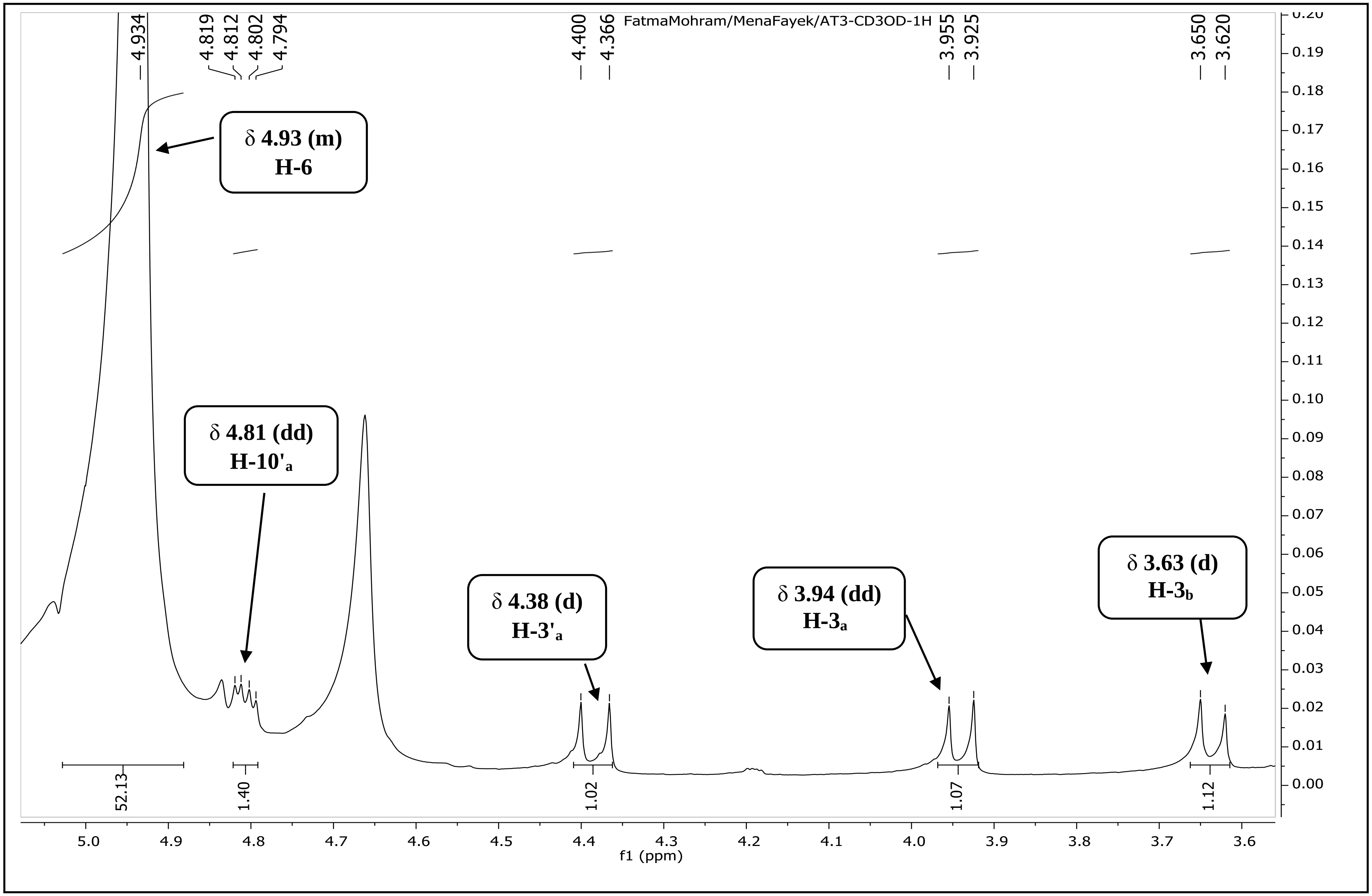

Figure S25 continue. ^{1}**HNMR spectrum of compound 4 (CD$_3$OD-*d*, 500 MHz)**

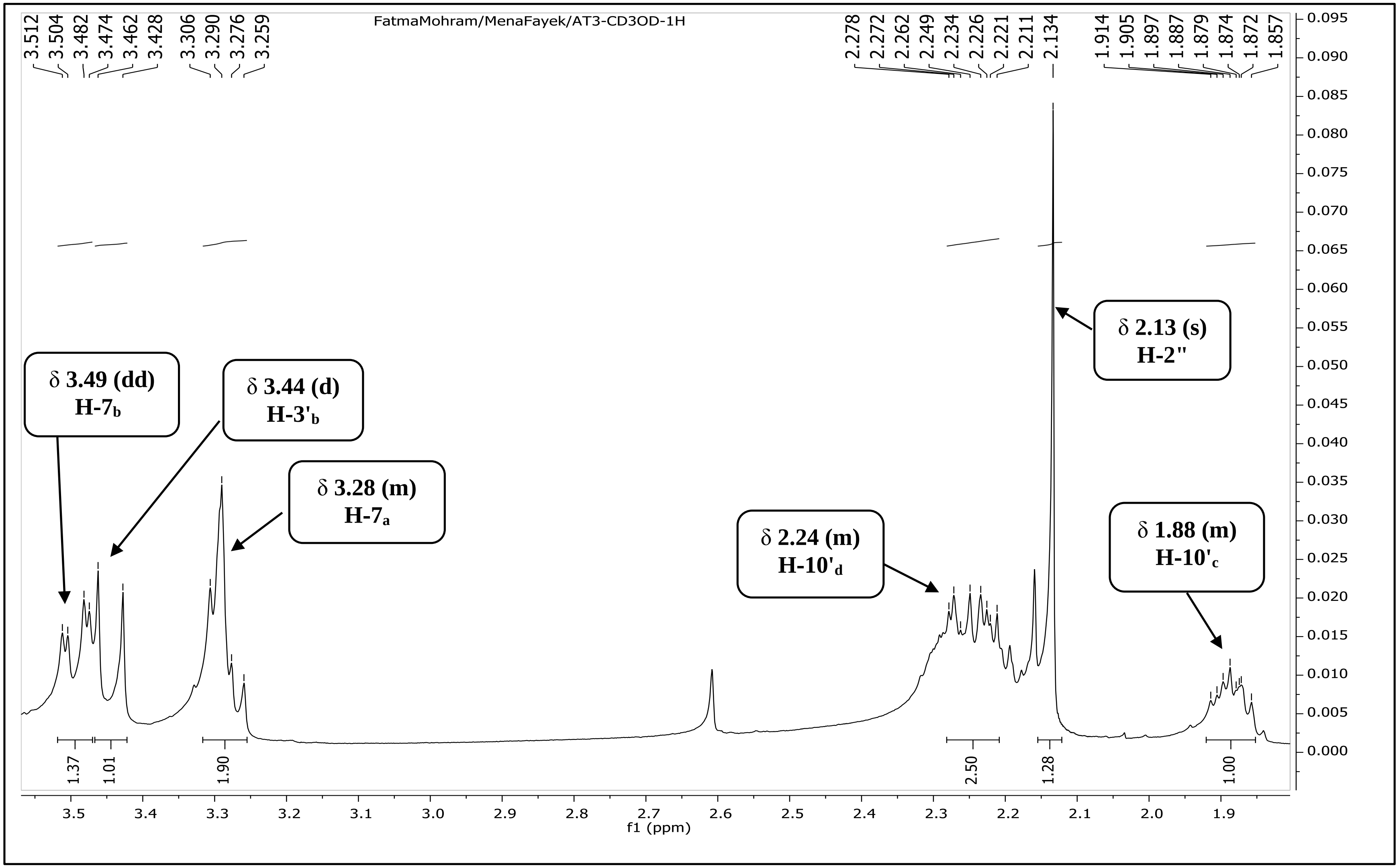

Figure S25 continue. ^{1}HNMR spectrum of compound 4 (CD_3OD-*d*, 500 MHz)

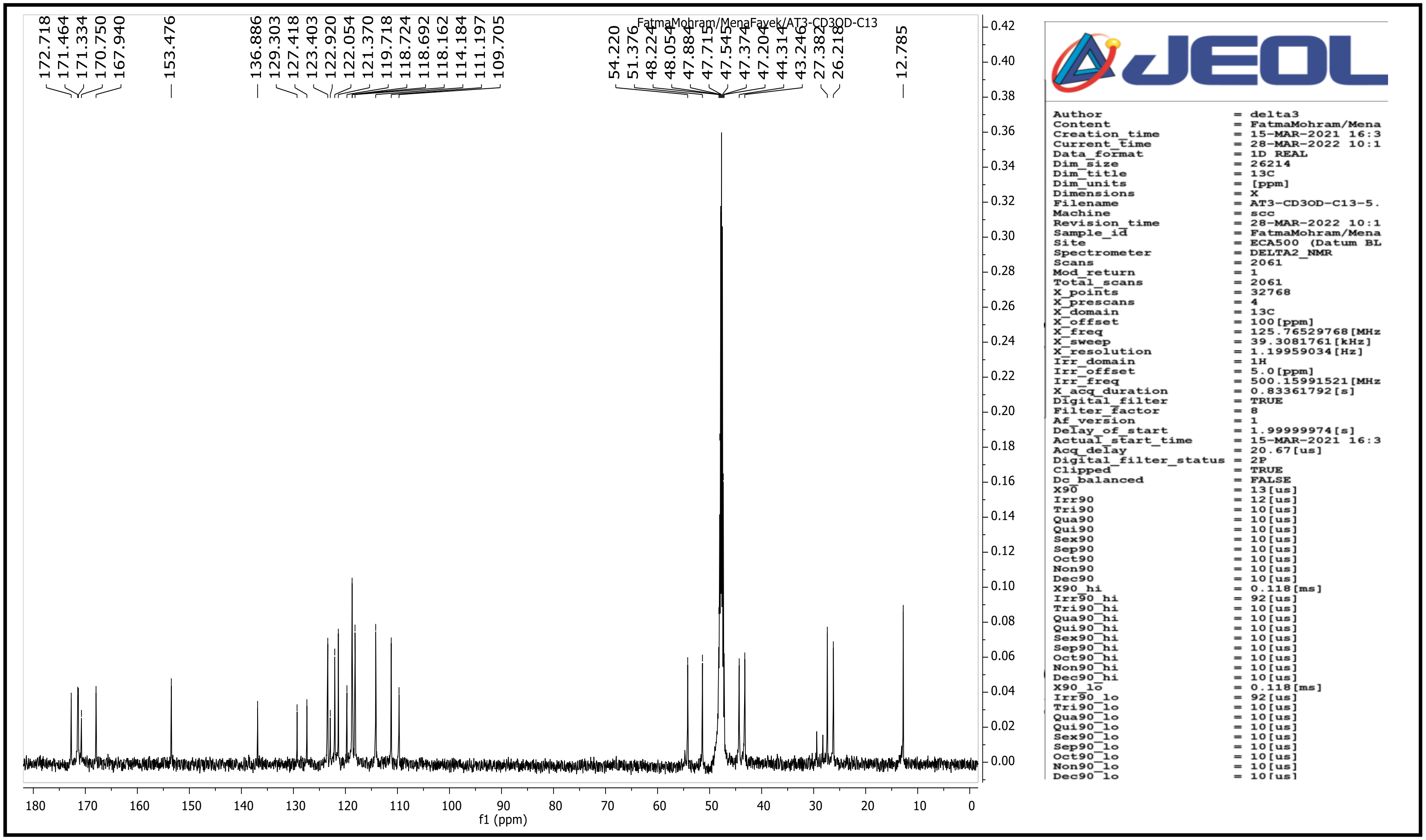

Figure S26. ^{13}CNMR spectrum of compound 4 (CD$_3$OD-*d*, 125 MHz)

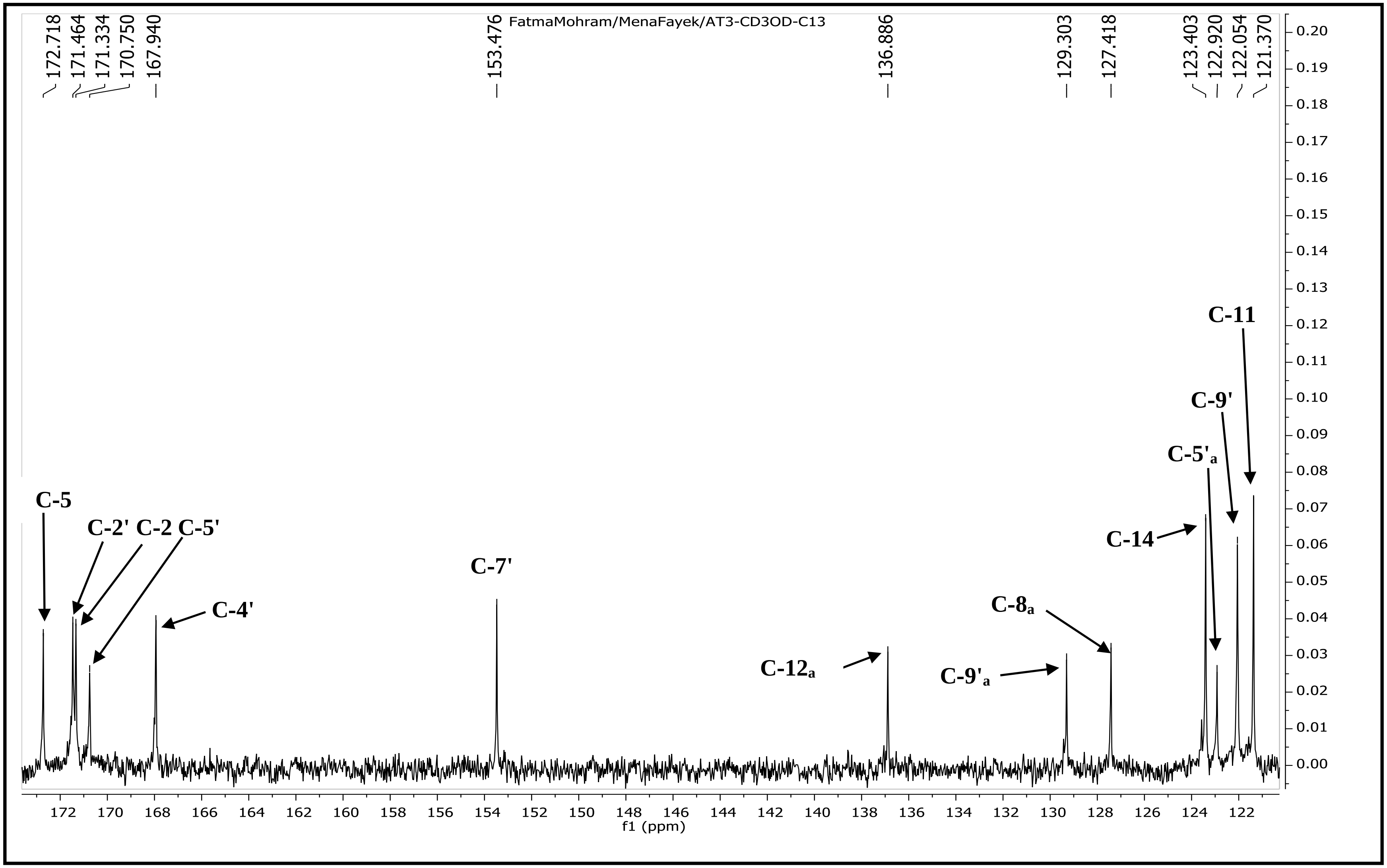

Figure S26 continue. ^{13}**CNMR spectrum of compound 4 (CD$_3$OD-*d*, 125 MHz)**

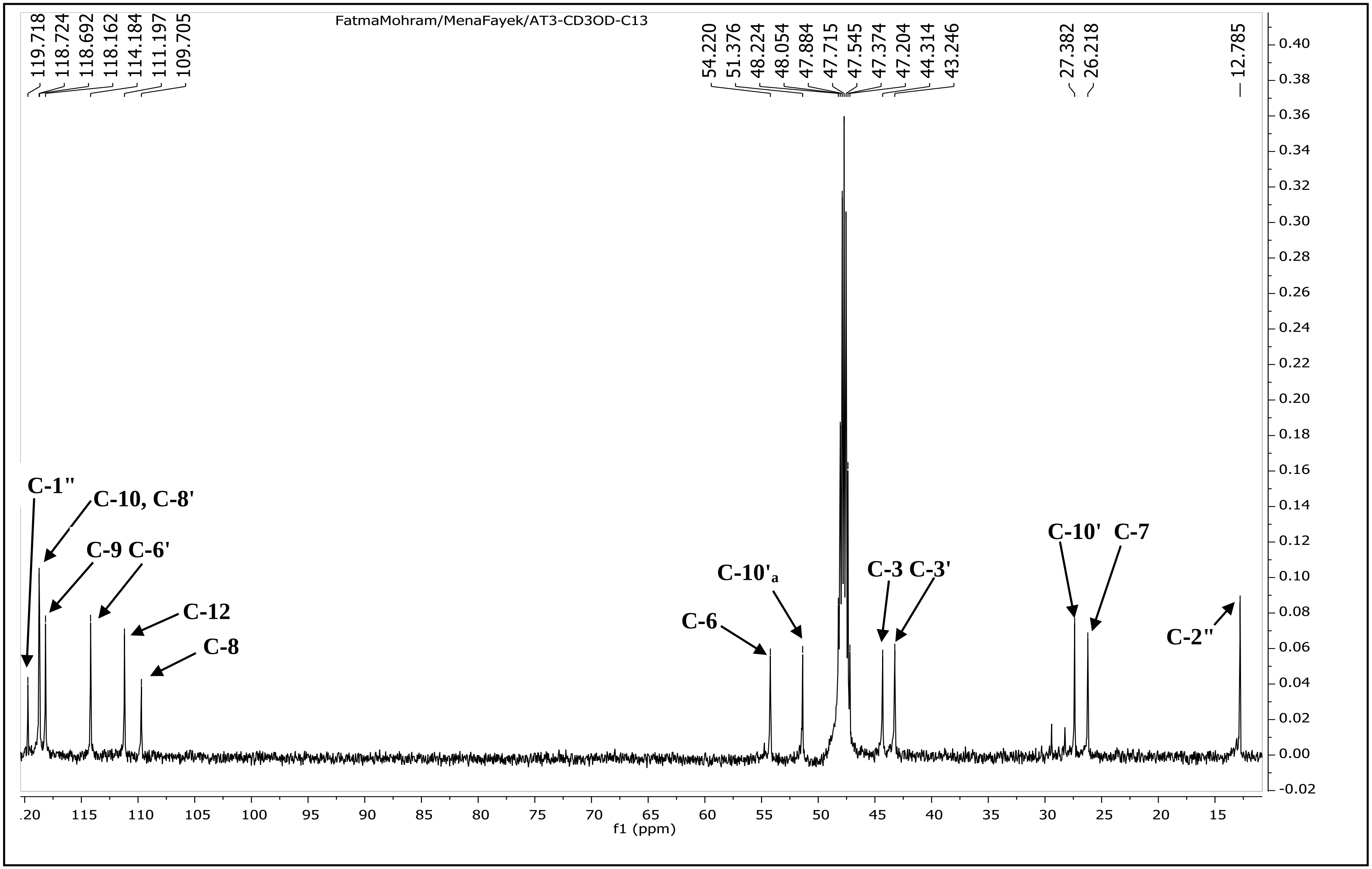

Figure S26 continue. ^{13}CNMR spectrum of compound 4 (CD_3OD-*d*, 125 MHz)

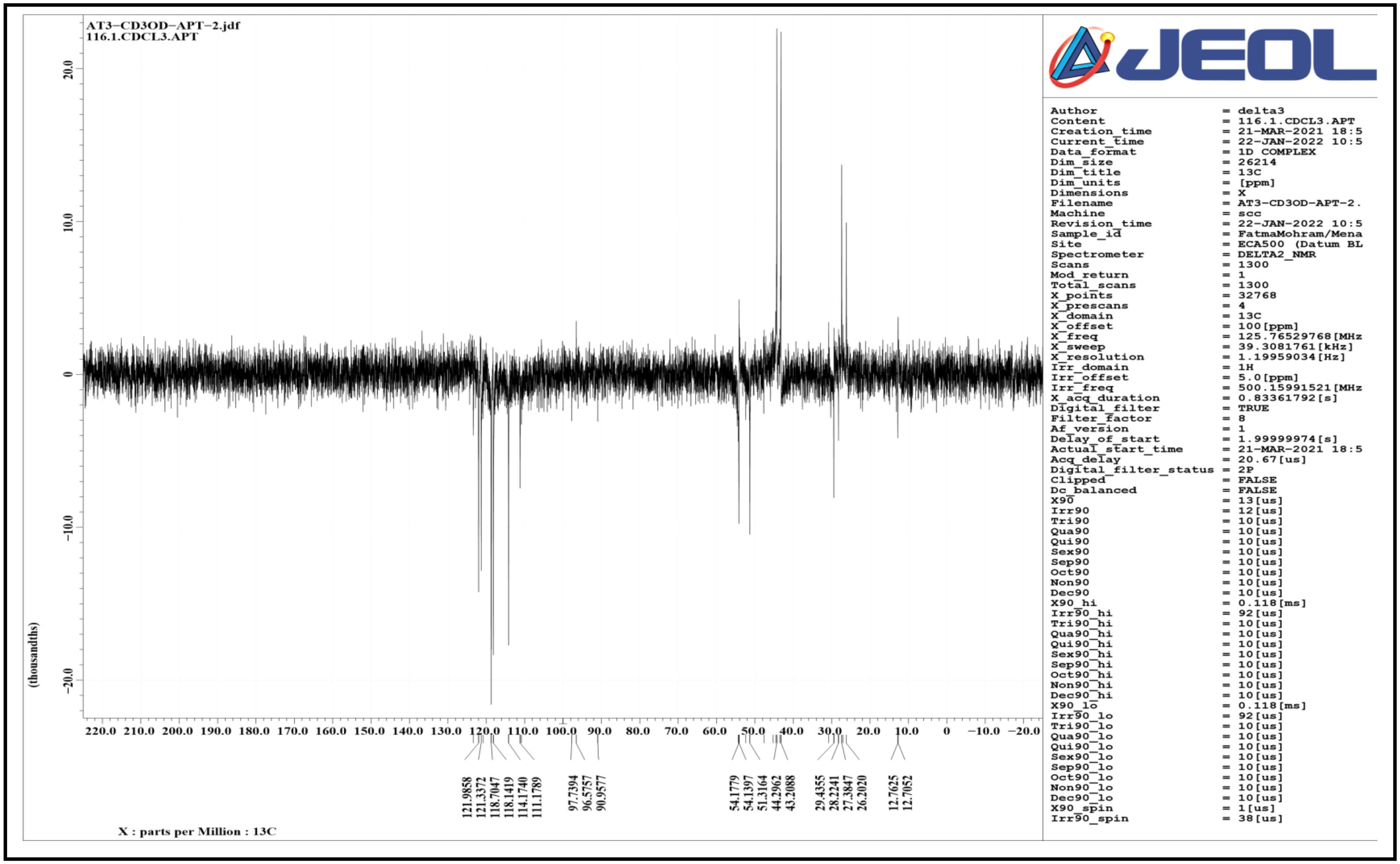

Figure S27. APT spectrum of compound 4 (CD_3OD-*d*, 125 MHz)

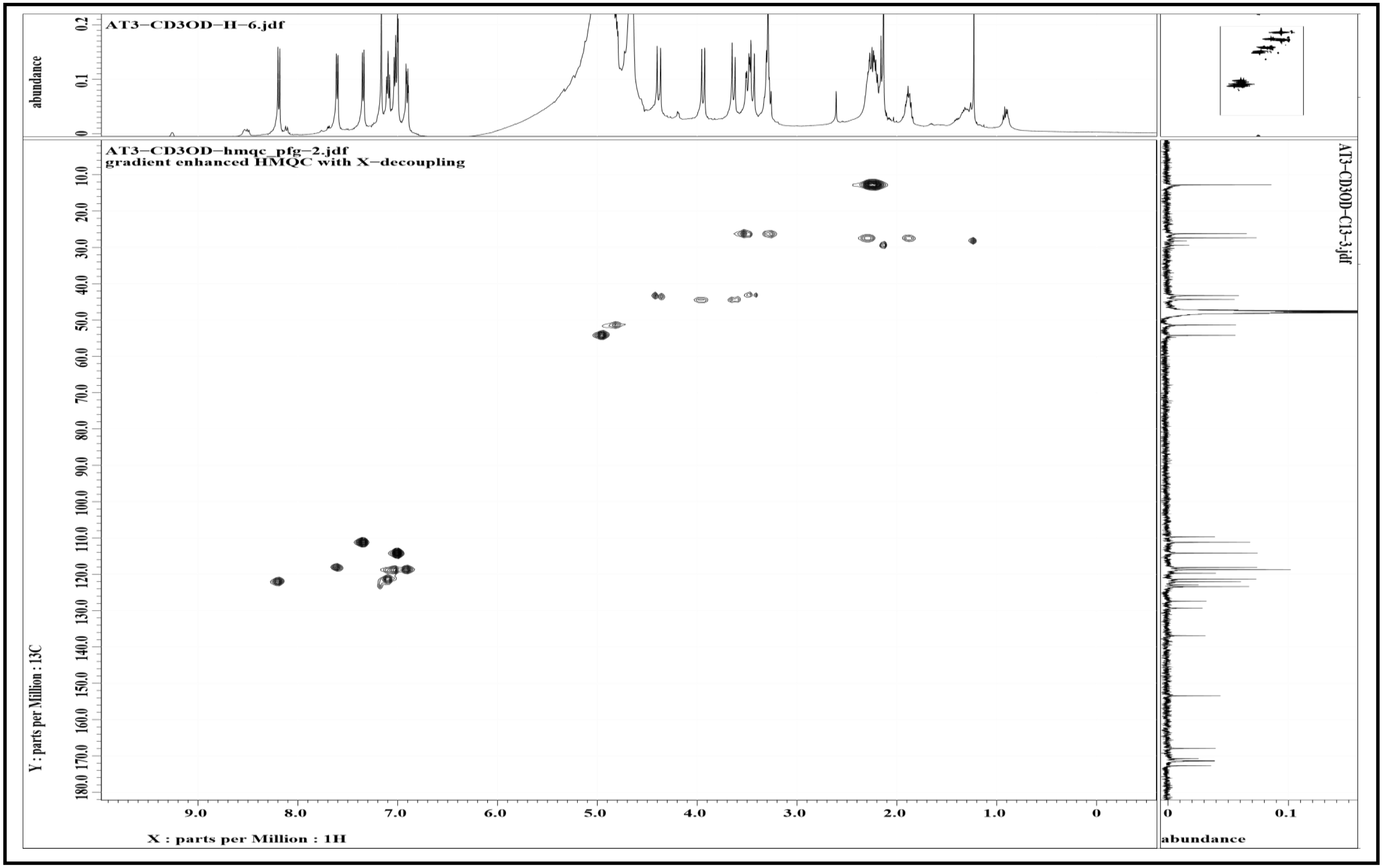

Figure S28. HSQC spectrum of compound 4 (CD_3OD-*d*, 500 MHz)

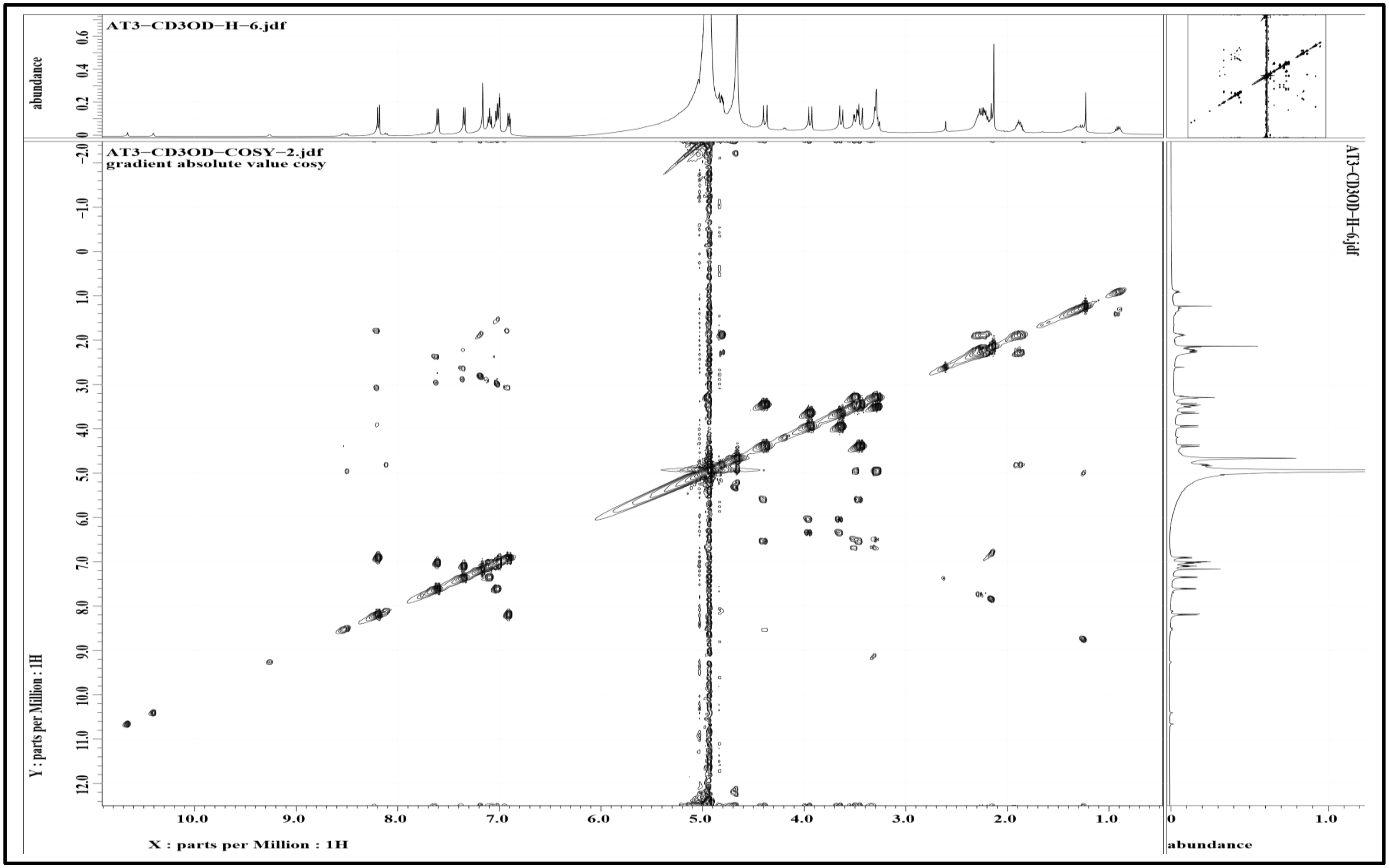

Figure S29. **^{1}H-^{1}H COSY spectrum of compound 4 (CD_3OD-*d*, 500 MHz)**

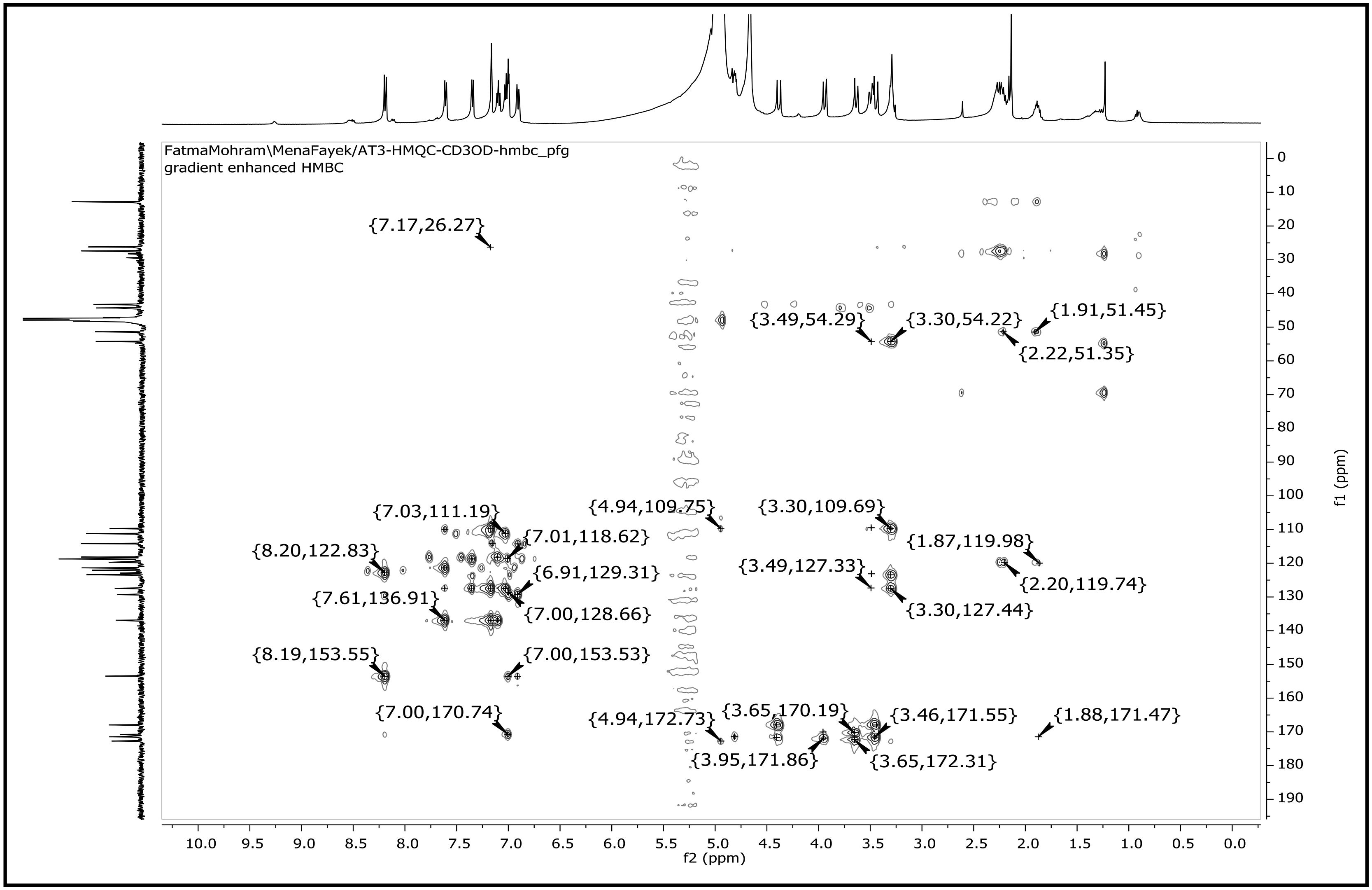

Figure S30. HMBC spectrum of compound 4 (CD_3OD-*d*, 500 MHz)

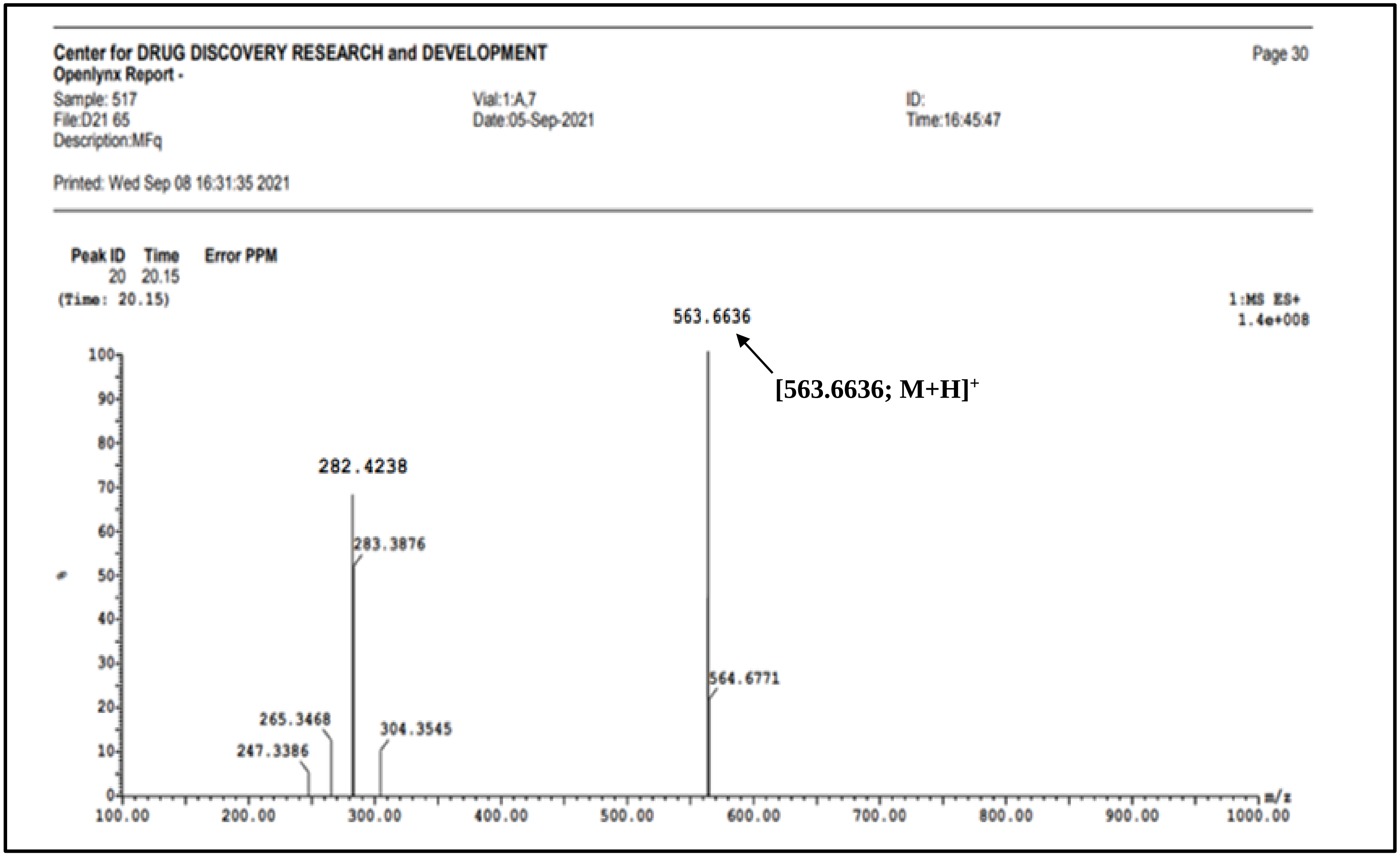

Figure S31. Positive ESI/MS spectrum of compound 4

BIO TECHNOLOGY ◻ CULTURE COLLECTION ◻ MANPOWER TRAINING
BIOFERTILIZERS ◻ NETWORK DEVELOPMENT

Microorganism Deposition Certificate

The Egypt Microbiological Culture Collection (EMCC, CAIRO MIRCEN) certifies that:

Prof./ Fatma Abd -Elkader Moharram

Dr./ Menna Fayek

have delivered one microbial isolate of: *Aspergillus terreus* MF-9 EGY MW035847 isolated from an agriculture field (10 cm depth, Dekernis, Dakahlia Egypt). The isolate has been deposited at the Department of: Microbial Chemistry, Genetic Engineering and Biotechnology Division, National Research Centre, Cairo 12622, Egypt, and the necessary identifications have been performed.

The isolate has been deposited at the Egypt Microbiological Culture Collection (EMCC) with an EMCC number 28559 on 29 -03-2023.

EMCC will preserve and maintain the mentioned isolate based on the depositors' request, with a fee of 2000 pounds (two thousand Egyptian pounds).

This is a certificate stating the strain of microorganisms' deposition procedures integrity.

Director of the CAIRO MIRCEN

Dr. Mustafa Abdullah Ahmed

29-03-2023

Mustafa A. Ahmed

Cairo MIRCEN Faculty Of Agriculture ASU
PO Box 68 Hadayek Shoubra , Cairo 11241 Egypt
Tel -02744454860 2344454962 Mobile 01028475714 01204868426

[illegible]

Figure S32. Microorganism deposition Certificate

Table S1: HPLC/ESI-MS tentative identification for metabolites in *A. terreus* ethyl acetate extract

Rt (min)	MS ion peak [M-H]$^-$	[M+H]$^+$	M.F	Potential metabolite(s)	Reference
6.99	-	405.3152	$C_{25}H_{40}O_4$	Aspterpenacid B **(1)**	Liu *et al*., 2016
	-	405.3152	$C_{25}H_{40}O_4$	Terretonin O (**2**)	Hamed *et al*., 2020
	-	405.3152	$C_{24}H_{36}O_5$	Lovastatin (**3**)	Wang *et al*., 2018
8.25	355.2175	357.1848	$C_{19}H_{16}O_7$	Butyrolactone II **(4)**	Hamed *et al*., 2020
9.38	-	432.2634	$C_{25}H_{25}N_3O_4$	Asterrelenin **(5)**	Shaaban *et al*., 2018
	-	432.2634	$C_{18}H_{16}Cl_2O_8$	Methyl dichloroasterrate **(6)**	Nong *et al*., 2015
9.66	439.2957	441.2694	$C_{24}H_{24}O_8$	Butyrolactone III **(7)**	Uras *et al*., 2021
	439.2957	441.2694	$C_{24}H_{24}O_8$	Butyrolactone IV **(8)**	Yang *et al*., 2018
	439.2957	441.2694	$C_{24}H_{24}O_8$	Butyrolactone V **(9)**	Cheng *et al*., 2019
	439.2957	441.2694	$C_{25}H_{28}O_7$	Versicolactone G **(10)**	Liu *et al*., 2018
10.84	189.2393	191.2361	$C_{12}H_{14}O_2$	4-hydroxy-3-(3-methyl-but-2-enyl)-benzaldehyde **(11)**	Uras *et al*., 2021
11.20	533.3345	-	$C_{24}H_{26}N_2O_8S_2$	Asterriquinone A1 **(12)**	Arai *et al*., 1981
	533.3345	-	$C_{34}H_{34}N_2O_4$	Asterriquinone B1 **(13)**	Arai *et al*., 1981
	533.3345	-	$C_{34}H_{34}N_2O_4$	Asterriquinone B2 **(14)**	Arai *et al*., 1981
	533.3345	-	$C_{34}H_{34}N_2O_4$	Asterriquinone A3 **(15)**	Girich *et al*., 2020
	533.3345	-	$C_{34}H_{34}N_2O_4$	Asterriquinone B4 **(16)**	Girich *et al*., 2020
11.21	-	489.3256	$C_{26}H_{32}O_9$	Terretonin **(17)**	Uras *et al*., 2021
	-	489.3256	$C_{27}H_{36}O_8$	Asperterpene H **(18)**	Qi *et al*., 2018a
	-	489.3256	$C_{28}H_{40}O_7$	Spiroterreusnoid C **(19)**	Qi *et al*., 2019
11.4	423.3059	425.3559	$C_{24}H_{24}O_7$	Butyrolactone I **(20)**	Uras *et al*., 2021
	423.3059	425.3559	$C_{24}H_{24}O_7$	Aspernolide N **(21)**	Cheng *et al*., 2019
	423.3059	425.3559	$C_{24}H_{24}O_7$	Aspernolide A **(22)**	Parvatkar *et al*., 2009
	423.3059	425.3559	$C_{24}H_{24}O_7$	Asperlide A **(23)**	Qi *et al*., 2018b
	423.3059	425.3559	$C_{24}H_{24}O_7$	Butyrolactone VIII **(24)**	Ma *et al*., 2014
11.86	429.1663	-	$C_{24}H_{30}O_7$	Terretonin C **(25)**	Sun *et al*., 2018
	431.1636	-	$C_{27}H_{28}O_5$	Aspulvinone B **(26)**	Ojima *et al*., 1975
	431.1636	-	$C_{27}H_{28}O_5$	Aspulvinone H **(27)**	Chen *et al*., 2018
12.18	473.3696	475.3733	$C_{28}H_{34}N_4O_3$	Aspergillamide A **(28)**	Xiao *et al*., 2019
	473.3696	475.3733	$C_{28}H_{34}N_4O_3$	Aspergillamide B **(29)**	Xiao *et al*., 2019
	473.3696	475.3733	$C_{26}H_{34}O_8$	Terretonin D **(30)**	Wu *et al*., 2019
	473.3696	475.3733	$C_{26}H_{34}O_8$	Terretonin D1 **(31)**	Wu *et al*., 2019
	473.3696	475.3733	$C_{26}H_{34}O_8$	Terreustoxin E **(32)**	Feng *et al*., 2019
	473.3696	475.3733	$C_{26}H_{34}O_8$	Terreustoxin F **(33)**	Feng *et al*., 2019
	473.3696	475.3733	$C_{26}H_{34}O_8$	Asperterpene G **(34)**	Qi *et al*., 2018a
	473.3696	475.3733	$C_{26}H_{34}O_8$	Austalide B **(35)**	Shan *et al*., 2015
13.10	-	473.3118	$C_{26}H_{32}O_8$	Terretonin A **(36)**	Li *et al*., 2019
16.58	445.4681	-	$C_{27}H_{42}O_5$	Aspterpenacid A **(37)**	Liu *et al*., 2016
	445.4681	-	$C_{26}H_{38}O_6$	Terretonin M **(38)**	Hamed *et al*., 2020
22.25	-	284.3968	$C_{16}H_{17}N_3O_2$	Brevianamide F **(39)**	Xiao *et al*., 2019
23.13	-	507.5596	$C_{27}H_{38}O_9$	Terretonin G **(40)**	Li *et al*., 2019
	-	507.5596	$C_{27}H_{38}O_9$	Terreustoxin J **(41)**	Feng *et al*., 2019
	-	507.5596	$C_{27}H_{38}O_9$	Asperterpene J **(42)**	Qi *et al*., 2018a
	-	507.5596	$C_{27}H_{38}O_9$	Terreusterpene A **(43)**	Qi *et al*., 2018c
	-	507.5596	$C_{32}H_{30}N_2O_4$	Asterriquinone **(44)**	Yamamoto *et al*., 1976

Table (S2): ^{1}H and ^{13}CNMR data of compound 2 and reference compound ($CDCl_3$, 500 MHz and 125 MHz)

C No	δ_C (ppm)		δ_H (ppm)		^{1}H-^{1}H COSY	HMBC
	2	Ref *	2	Ref *		
**1**	169.4	169.8				
**2**	-	144.9				
**3**	138.9	137.7				
**4**	85.6	86.2				
**5**	38.4	38.7	3.43 (d, 14.5)	3.53 (d, 15.0)		C-4, C-6, C-6", C-1", C-2"
			3.38 (d, 14.5)	3.47 (d, 15.0)		
**6**	170.5	170.1				
**7**	52.6	53.7	3.76 (s)	3.75 (s, 3H)		C-6
**1'**	122.1	121.8				
**2'/6'**	129.0	129.6	7.57 (d,8.5)	7.61 (d, 8.8)	H-3'/5'	C-3'/5', C-4'
**4'**	157.9	157.2				
**3'/5'**	115.4	116.1	6.86 (d,8.5)	6.90 (d, 8.8)	H-2'/6'	C-1', C-4'
**1"**	123.9	124.6				
**2"**	131.1	131.8	6.38 (br s)	6.51 (d, 2.0)		C-5, C-7", C-6", C-4"
**3"**	127.2	128.8				
**4"**	153.7	153.2				
**5"**	113.9	115.1	6.48 (d, 8.0)	6.52 (d, 8.1)		C-1", C-3"
**6"**	128.5	129.2	6.53(dd, 2.0, 8.0)	6.59 (dd, 8.1, 2.0)		C-5, C-2", C-4"
**7"**	27.4	28.7	3.06 (d, 5.0)	3.12 (d, 7.2, 2H)	H-8″	C-8", C-3", C-9", C-4"
**8"**	122.2	121.8	5.04 (td, 7.5, 8.0)	5.08 (td, 6.7, 5.7, 3.8)	H-7″	C-7", C-10", C-11"
**9"**	131.8	133.9				
**10"**	24.7	25.7	1.64 (s)	1.65 (s, 3H)		C-8", C-9", C-11"
**11"**	16.5	17.7	1.55 (s)	1.59 (s, 3H)		C-8", C-9", C-10"

Value between parenthesis represent the *J* value in Hz; * (Uras *et al*., 2021; $CDCl_3$-*d*, 400 MHZ).

Table (S3): ^{1}H and ^{13}CNMR data for compound 3 and reference compound ($CDCl_3$, 500 MHz and 125 MHz)

C. No	δ C (ppm)		δ H (ppm)		^{1}H-^{1}H COSY	HMBC
	3	Ref data*	3	Ref data*		
**1**	169.7	169.6				
**2**	139.5	137.6				
**3**	127.4	129.7				
**4**	85.5	86.1				
**5**	38.3	38.8	3.41, d (10.5)	3.51, d (14.7)<br>3.41, d (14.7)		C-4, C-6, C-1", C-2",<br>C-6"
**6**	170.5	169.9				
**7**	52.6	53.7	3.76, (s)	3.73, s (3H)		C-6
**1'**	122.2	122				
**2'/6'**	129.1	129.6	7.55, d (8.5)	7.56, d (9.0)	H-3'/5'	C-3, C-4' C-3'/5'
**4'**	157.8	157.3				
**3'/5'**	115.3	116.2	6.85, d (8.5)	6.90, d (9.0)	H-2'/6'	C-1', C-4', C-2'/6'
**1"**	119.3	118.4				
**2"**	131.6	132.1	6.47, d (2.0)	6.51, d (2.1)		C-5, C-4", C-6", C-7"
**3"**	124.9	124.8				
**4"**	152.1	152.0				
**5"**	115.9	116.7	6.45, d (8.5)	6.53, d (8.1)		C-1", C-3"
**6"**	128.9	128.9	6.51, dd (8.5, 2.0)	6.51, dd (8.1, 2.1)		C-5, C-1", C-2", C-4"
**7"$_a$**	30.7	30.9	2.79, dd (17.0, 7.5)	2.82, dd (17.0, 5.0)	H-7"$_b$, H-8"	C-2", C-4", C-8", C-9"
			2.49, dd (17.5, 7.5)		H-7"$_a$,	C-2", C-4", C-8", C-9"
**7"$_b$**				2.58, dd (17.0, 6.0)	H-8"	
**8"**	69.2	69.7	3.64, dd (7.5, 5.5)	3.74, m	H-7"$_{a/b}$	C-10", C-11"
**9"**	76.7	76.8				
**10"**	19.6	22.0	1.15, s (s)	1.24, s (3H)		C-8", C-9", C-11"
**11"**	24.6	24.8	1.24, s (s)	1.21, s (3H)		C-8", C-9", C-10"

Table S4: The primer sequences used for PCR amplification.

Gene	Primer	Sequence
**IL-6**	Forward	5’-AGACAGCCACTCACCTCTTCAG-3’
	Reverse	5'-TTCTGCCAGTGCCTCTTTGCTG-3'
**TNF-α**	Forward	5’-CTCTTCTGCCTGCTGCACTTTG-3’
	Reverse	5'-ATGGGCTACAGGCTTGTCACTC-3'
**β-actin**	Forward	5’-GCACCACACCTTCTACAATG-3’
	Reverse	5’-TGCTTGCTGATCCACATCTG-3’

Discussion of compound 2

It was purified as pale yellow powder; R_f = 0.37 **(S_4).** It gave violet color upon spraying and heating with anisaldehyde/ sulfuric acid reagent. Negative ESI-MS: *m/z*: 423. 2239 $[M-H]^-$; 847.4105 $[2M+H]^-$ ^{1}HNMR data showed in the aromatic region two *ortho* doublets at δ_H 7.57 and 6.86 for H-2'/6' and H-3'/5' respectively which confirm the presence of 1', 4'-disubstituted aromatic ring in addition to three signals at δ_H 6.53 (6"), 6.48 (5") 6.38 (2") which support the presence of another 1, 3, 4-trisubstituted aromatic ring. The presence of doublet signal at δ_H 3.06 (CH_2-7") integrated for two protons together with triplet one integrated for one proton at δ_H 5.04 (CH-8") and two singlet signals each integrated for three protons at δ_H 1.64 and 1.55 for two methyl groups at C-10" and C-11" respectively gave an evidence for the presence of isoprenyl group in the structure of **2**. Furthermore, the ^{1}HNMR spectrum showed singlet signal at δ_H 3.76 characteristic for one methoxy group (OCH_3-7) in addition to the presence of two doublet signals at δ_H 3.43 and 3.38 (14.5 Hz) support the presence of methylene group attached to sp^2 system (CH_2-5). The above finding was supported by ^{13}CNMR and APT spectrum which display twenty-four carbons among which twelve carbons signals at δ_C 115.4-157.9 in the aromatic region characteristic for the 1, 4- disubstituted and 1, 3, 4- trisubstituted aromatic rings. In addition, the presence of sp^3 methylene signal δ_C 27.4 (CH_2-7"), sp^3 methine signals δ_C 122.2 (CH-8"), quaternary carbon at δ_C 131.8 (C-9"), together with two methyl signals δ_C 24.7 (CH_3-10"), 16.5 (CH_3-11") confirm the presence of isoprenyl group in the structure. The presence of lactone ring in the structure of **2** was confirmed by the presence of carbonyl carbon at δ_C 169.4 (C-1) together with C-3 (δ_C 138.9) and C-4 (δ_C 85.6). Moreover, the carboxylic acid methyl ester group was established through the presence of carbonyl carbon δ_C 170.5 (C-6) with the methoxy group at δ_C 52.6. HMQC spectrum confirms that all non-exchangeable proton resonances were connected with the directly attached carbon atoms. ^{1}H-^{1}H-COSY spectrum

showed some correlation between protons of the aromatic ring as well as, that between protons of the isoprenyl group**.** Analysis of the HMBC spectrum establish the structure of **2** through the correlation of the proton with neighboring carbons**.** 1, 4-disubstituted aromatic ring was confirmed by the correlation of H-2'/6' (δ_H 7.57) with C-3'/5' (δ_C 115.4) and C-4' (δ_C 157.9) also that of H-3'/5' (δ_H 6.86) with C-1' (δ_C 122.1) and C-4' (δ_C 157.9). Moreover, 1, 3, 4- trisubstituted ring was established by the correlation of H-2'' (δ_H 6.38) with C-4'' (δ_C 153.7), C-6'' (δ_C 128.5), also between H-5'' (δ_H 6.48) with C-1'' (δ_C 123.9) and C-3'' (δ_C 127.2) in addition that from H-6'' (δ_H 6.53) to C-2'' (δ_C 131.1) and C-4'' (δ_C 153.7). The isoprenyl group was confirmed from the correlation of H-8'' (δ_H 5.04) with C-7'' (δ_C 27.4), C-10'' (δ_C 24.7) and C-11'' (δ_C 16.5) as well as correlation of H-10'' (δ_H 1.65) and H-11'' (δ_H 1.55) with C-8'' (δ_C 122.2) and C-9'' (δ_C 131.8) and that from H-7'' (δ_H 3.06) to C-8'' (δ_C 122.2) and C-9'' (δ_C 131.8). Moreover, its position attached at C-3'' was afforded form the correlation between H-7'' (δ_H 3.06) with C-3'' (δ_C 127.2) and C-4'' (δ_C 153.7). Furthermore, the attachment between lactone ring and trisubstituted ring at C-1'' was confirmed from the correlation between H-5 (δ_H 3.43 and 3.38) and C-1'' (δ_C 123.9), C-2'' (δ_C 131.1), C-6'' (δ_C 128.5) and C-4 (δ_C 85.6). Finally, the molecular weight of compound **2** was established from its negative ESI/MS which displayed a molecular ion peak at *m/z* 423.2239 $[M-H]^-$ corresponding to molecular formula $C_{24}H_{24}O_7$ and thirteen degrees of unsaturation. Therefore, on the basis of the above analysis for 1D and 2D NMR data as well as comparison with previously published data (Uras *et al.*, 2021) the structure of 2 was conventional as butyrolactone I which was isolated before from different *Aspergillus* species.

Discussion of compound 3

It was isolated in pure form as pale yellow powder; $R_f = 0.31$ (S_4). As in case of compound **2**, it gave violet color after spraying with anisaldehyde/ sulfuric acid reagent. Its negative ESI-MS is *m/z*: 439. 2230 $[M-H]^-$; 879.4200 $[2M+H]^-$

As shown from the molecular weight of **3**, there is an excess of 16 mn indicating the presences of additional oxygen atom compared with butyrolactone I **(2)** and also it possesses 13 degrees of unsaturation and corresponding to the molecular formula $C_{24}H_{24}O_8$. The ^{1}HNMR spectra of 3 are more or less the same of **2** except the up filed shift of H-7" at δ_H 2.79- 2.49 (ca 3.06 in compound **2**) and H-8" at δ_H 3.64 (ca 5.04 in compound **2**). Moreover, in case of ^{13}CNMR spectra**,** the signals of isoprenyl unit in case of **3** was replaced by two oxygenated sp^3 carbons distinguished as one methine carbon C-8" (δ_C 69.2) and the other is aliphatic quaternary carbon C-9" (δ_C 76.7) together with two singlet characteristic for methyl groups at δ_C 19.6 (δ_H 1.15) and δ_C 24.6 (δ_H 1.24), which confirm the presence of epoxy group in the structure of **3.** Moreover, the presence of the epoxy group was further established from ^{1}H-^{1}H-COSY which display a correlation between H-7" **(**δ_H 2.79, 2.49) with H-8" (δ_H 3.64), as well as the HMBC correlation between the two singlet methyl groups (δ_H 1.15 and 1.24) to C-8" (δ_C 69.2) and C-9" (δ_C 76.7). The assignment of ^{1}HNMR, ^{13}CNMR, ^{1}H-^{1}H-COSY and HMBC were represented in. Based on the analysis of the above data and comparison with previously reported literature, compound **3** was confirmed as butyrolactone III (Uras *et al*., 2021) which isolated before from *Aspergillus* species.

References for table S1

Arai K, Masuda K, Kiriyama N, NItta K, Yamamoto Y, Shimizu S. **(1981).** Metabolic products of *Aspergillus terreus*. IV. Metabolites of the strain ifo 8835. (2). The isolation and chemical structure of indolyl benzoquinone pigments. Chem Pharm Bull 29 (4), 961-969doi: 10.1186/1471-2393-5-14

Chen G, Ruan BH, Yang Y B, Wang Q, Li X Z, Luo N, Yang X Q, Zhao L X. (2018). Secondary metabolites of the fungus *Aspergillus terreus*. Chem Nat Compd 54 (2), 415-418. DOI:10.1007/s10600-018-2366-3

Cheng Z, Li Y, Liu W, Liu L, Liu J, Yuan W, et al (2019). Butenolide derivatives with α-glucosidase inhibitions from the deep-sea-derived fungus *Aspergillus terreus* YPGA10. Marine drugs 17 (6), 332. https://doi.org/10.3390/md17060332.

Feng W, Chen C, Mo S, Qi C, Gong J, et al., (2019). Highly oxygenated meroterpenoids from the Antarctic Fungus *Aspergillus terreus*. *Phytochemistry* 164, 184-191. doi: 10.1016/j.phytochem.2019.05.015. Epub 2019 May 31.

Girich EV, Yurchenko AN, Smetanina OF, Trinh PTH, Ngoc NTD, et al., (2020). Neuroprotective metabolites from vietnamese marine derived fungi of *Aspergillus* and *Penicillium* Genera. Marine drugs, 18 (12), 608. doi.org/10.3390/md18120608

Hamed A, Abdel-Razek AS, Omran DA, El-Metwally MM, El-Hosari DG, et al., (2020). Terretonin O: A new meroterpenoid from *Aspergillus terreus*. *Nat prod Res* 34 (7), 965-974. doi.org/10.1080/14786419.2018.1544977

Li HL, Li XM, Li X, Yang S, Wang BG. (2019). Structure, absolute configuration and biological evaluation of polyoxygenated meroterpenoids from the marine algal-derived *Aspergillus terreus* EN-539. *Phytoch Lett* 32, 138-142.

Liu M, Sun W, Wang J, He Y, Zhang J, et al., (2018). Bioactive secondary metabolites from the marine-associated fungus *Aspergillus terreus*. Bioorg Chem 80, 525-530. doi.org/10.1016/j.bioorg.2018.06.029.

Liu Z, Chen Y, Chen S, Liu Y, Lu Y et al., (2016). Aspterpenacids A and B, two sesterterpenoids from a mangrove endophytic fungus *Aspergillus terreus* H010. Org Lett 18(6), 1406-1409. https://doi.org/10.1021/acs.orglett.6b00336

Ma X, Zhu T, Gu Q, Xi R, Wang W, Li D. (2014). Structures and antiviral activities of butyrolactone derivatives isolated from *Aspergillus terreus* MXH-23. J. Ocean Univ. China, 13(6), 1067-1070. doi10.1007/s11802-014-2324-z.

Nong XH, Zhang XY, Xu XY, Qi SH. (2015). Antifouling Compounds from the Marine-Derived Fungus *Aspergillus terreus* SCSGAF0162. Nat Prod Commun 10(6):1033-4.

Ojima N, Takenaka S, Seto, S. (1975). Structures of pulvinone derivatives from *Aspergillus terreus*. *Phytochemistry* 14(2), 573-576. doi.org/10.1016/0031-9422(75)85131-4

Parvatkar RR, D'Souza C, Tripathi A, Naik CG. (2009). Aspernolides A and B, butenolides from a marine-derived fungus *Aspergillus terreus*. *Phytochemistry* 70(1), 128-132. doi: 10.1016/j.phytochem.2008.10.017. Epub 2008 Dec 10.

Qi C, Gao W, Guan D, Wang J, Liu M, et al., (2018 b). Butenolides from a marine-derived fungus *Aspergillus terreus* with antitumor activities against pancreatic ductal adenocarcinoma cells. Bioorg Med Chem, 26(22), 5903-5910. doi: 10.1016/j.bmc.2018.10.040. Epub 2018 Oct 29.

Qi C, Liu M, Zhou Q, Gao W, Chen C, et al., (2018 a). BACE1 inhibitory meroterpenoids from *Aspergillus terreus*. *J Nat prod* 81(9), 1937-1945. doi.org/10.1021/acs.jnatprod.7b01050

Qi C, Qiao Y, Gao W, Liu M, Zhou Q et al., (2018 c)**.** New 3, 5-dimethylorsellinic acid-based meroterpenoids with BACE1 and AchE inhibitory activities from *Aspergillus terreus*. Org Biomol Chem , 16(46), 9046-9052. doi.org/10.1039/c8ob02741b.

Qi C, Zhou Q, Gao W, Liu M, Chen C, et al., (2019). Anti-BACE1 and anti-AchE activities of undescribed spiro-dioxolane-containing meroterpenoids from the endophytic fungus *Aspergillus terreus* Thom. *Phytochemistry*, 165, 112041. doi.org/10.1016/j.phytochem.2019.05.014

Shaaban M, El-Metwally M M, Abdel-Razek A A, Laatsch H. (2018). Terretonin M: A new meroterpenoid from the thermophilic *Aspergillus terreus* TM8 and revision of the absolute configuration of penisimplicins. Nat prod Res 32(20), 2437-2446. doi: 10.1080/14786419.2017.1419230. Epub 2017 Dec 27

Shan WG, Wu ZY, Pang WW, Ma LF, Ying YM, et al., (2015). α-Glucosidase inhibitors from the fungus *Aspergillus terreus* 3.05358. Chem Biodivers, 12(11), 1718-1724. DOI: 10.1002/cbdv.201500027

Sun K, Zhu G, Hao J, Wang Y, Zhu W. (2018). Chemical-epigenetic method to enhance the chemodiversity of the marine algicolous fungus, *Aspergillus terreus* OUCMDZ-2739. Tetrahedron, **74**(1), 83-87. doi.org/10.1016/j.tet.2017.11.039.

Uras S, Ebada SS, Korinek M, Albohy A, Abdulrazik BS, et al., (2021). Anti-Inflammatory, antiallergic, and COVID-19 main protease (Mpro) inhibitory activities of butenolides from a marine-derived fungus *Aspergillus terreus*. Molecules, 26(11), 3354. doi.org/10.3390/molecules26113354.

Wang Q, Yang YB, Yang XQ, Miao CP, Li YQ, et al., (2018). Lovastatin analogues and other metabolites from soil-derived *Aspergillus terreus* YIM PH30711. *Phytochemistry*, 145, 146-152. doi.org/10.1016/j.phytochem.2017.11.006.

Wu CJ, Cui X, Xiong B, Yang MS, Zhang YX, et al., (2019). Terretonin D1, a new meroterpenoid from marine-derived *Aspergillus terreus* ML-44. Nat Prod Res, 33(15), 2262-2265. doi.org/10.1080/14786419.2018.1493583

Xiao-Wei LUO, Yun LIN, Yong-Jun LU, Xue-Feng ZHOU, Yong-Hong LIU. (2019). Peptides and polyketides isolated from the marine sponge-derived fungus *Aspergillus terreus* SCSIO 41008. Chin. J. Nat. Med 17(2), 149-154. https://doi.org/10.1016/S1875-5364(19)30017-2

Yamamoto Y, Nishimura K Kiriyama N (1976). Studies on the metabolic products of Aspergillus terreus. I. Metabolites of the strain IFO 6123. Chem. Pharm. Bull 24(8), 1853-1859. DOI:10.1248/CPB.25.2593

Yang LH, Ou-Yang H, Yan X, Tang BW, Fang MJ, et al., (2018). Open-ring butenolides from a marine-derived anti-neuroinflammatory fungus *Aspergillus terreus* Y10. Marine drugs, 16(11), 428. doi.org/10.3390/md16110428
